# *Bacteroidetes* promote hepatocellular carcinoma progression and resistance to immunotherapy

**DOI:** 10.1097/HC9.0000000000001086

**Published:** 2026-11-11

**Authors:** Marina Barcena-Varela, Joan Shang, Anthony Lozano, Ilaria Mogno, Ian Liebling, Kai Mead, Zhihua Li, Lauren T. Grinspan, Katherine E. Lindblad, Marina Ruiz de Galarreta, Romain Donne, Sacha Gnjatic, Miriam Merad, Tomi Jun, Celina Ang, Thomas U. Marron, Jeremiah J. Faith, Amaia Lujambio

**Affiliations:** 1 Department of Oncological Sciences, Icahn School of Medicine at Mount Sinai, New York, New York, USA; 2 Marc and Jennifer Lipshultz Precision Immunology Institute, Icahn School of Medicine at Mount Sinai, New York, New York, USA; 3 Liver Cancer Program, Division of Liver Diseases, Department of Medicine, Tisch Cancer Institute, Icahn School of Medicine at Mount Sinai, New York, New York, USA; 4 Grupo de Fisiopatología e Historia Natural de las Enfermedades Hepáticas y Gastroenterológicas, Instituto de Investigación Sanitaria Hospital Gregorio Marañón, Comunidad de Madrid, Spain; 5 Department of Immunology and Immunotherapy, Icahn School of Medicine at Mount Sinai, New York, New York, USA; 6 Recanati/Miller Transplantation Institute, Mount Sinai Hospital, New York, New York, USA; 7 Division of Hematology and Medical Oncology, Icahn School of Medicine at Mount Sinai, New York, New York, USA; 8 Early Phase Trials Unit, The Tisch Cancer Institute at Mount Sinai, New York, New York, USA

**Keywords:** antibiotics (ABXs), fecal microbiome transplant (FMT), gut microbiome, hepatocellular carcinoma (HCC), immune checkpoint blockade (ICB)

## Abstract

**Background and Aims:**

Growing evidence highlights the gut microbiome’s critical role in tumorigenesis and immunotherapy response. However, the impact of gut microbes on hepatocellular carcinoma (HCC) progression and response to immune checkpoint blockade (ICB) remains unclear due to the lack of combined preclinical and clinical studies.

**Approach and Results:**

We performed 16S rRNA sequencing of cross-cohort stool samples from 10 HCC responders (R) and 40 non-responders (NR) to ICB at baseline and on-treatment time points. We identified *Bacteroidetes* enrichment in NR. To study the microbiome’s role in cancer immune response, we generated an immunogenic mouse model of HCC via hydrodynamic tail-vein injection (HDTVI) of DNA plasmids mimicking common HCC alterations and immunogenicity by expressing OVA model antigens (*MYC-lucOS;CTNNB1* tumors). We found that antibiotic (ABX)-induced dysbiosis promoted a pro-tumorigenic effect in the *MYC-lucOS;CTNNB1* HCC and reshaped the tumor immune microenvironment by reducing dendritic cells (DCs) through the expansion of the *Bacteroidetes* species, *Parabacteroides distasonis*. *P. distasonis* colonization in mice harboring *MYC-lucOS;CTNNB1* liver tumors confirmed its pro-tumorigenic effect in vivo. Furthermore, fecal matter transplant of patient microbiotas into murine recipients demonstrated that microbiota from an NR donor enriched in *Bacteroidetes* promoted faster tumorigenesis compared with microbiota from an R donor with reduced *Bacteroidetes*. We isolated 6 *Bacteroidetes* species from the NR donor into a bacterial cocktail. After colonization with the *Bacteroidetes* cocktail, mice harboring *MYC-lucOS;CTNNB1* HCC exhibited increased tumorigenesis, reduced survival, and impaired response to ICB.

**Conclusions:**

This study identified *Bacteroidetes* enrichment as a potential biomarker of ICB resistance in HCC and, by using immunogenic mouse models, established that *Bacteroidetes* abundance influences tumor development and response to ICB.

## INTRODUCTION

Hepatocellular carcinoma (HCC) is the most common type of primary liver cancer and the third leading cause of cancer-related mortality worldwide, affecting nearly 900,000 patients annually.^1^ Although the introduction of immune checkpoint blockade (ICB) in the treatment of HCC has improved clinical outcomes, most patients fail to achieve a clinical response, highlighting the need for predictive biomarkers. Anti-PD-1 inhibitors were the first ICB strategy tested in patients with HCC, showing unprecedented responses in approximately one-quarter of patients.^2^,^3^ However, the low overall response rate (ORR) of 15%–20% highlights the existence of mechanisms driving resistance to ICB.^2^,^3^ The combination of atezolizumab (anti-PD-L1) with bevacizumab (anti-VEGF) became the new standard of care (SOC) for first-line unresectable advanced HCC in 2020 after demonstrating improved ORR (~30%) and overall survival (OS) compared with sorafenib.^4^,^5^ However, most patients (~70%) still do not respond to any available treatment.

Several studies have focused on understanding different genetic and molecular mechanisms that can trigger sensitivity to ICB. To date, the only approved tumor-agnostic biomarkers for PD-1 blockade include microsatellite instability (MSI) or mismatch repair deficiency (dMMR), and tumor mutational burden (TMB). However, these markers are very limited in HCC, where MSI-H/dMMR have a prevalence of 0%–2.9%,^6^ and high TMB accounts for <1% of patients with HCC.^7^ High membrane expression of PD-L1 in tumor or immune cells has been associated with better OS in patients after treatment with anti-PD(L)-1; however, this biomarker is not used in clinical practice.^8^ Interferon signaling and major histocompatibility complex-related genes have also been identified as molecular patterns of HCC lesions that respond to anti-PD-1.^9^ Reduced clinical benefit of atezolizumab/bevacizumab in HCC has been associated with a high regulatory T cell (Treg) to effector T-cell (Teff) ratio, and with the expression of oncofetal genes (*GPC3*, *AFP*), while better outcomes from the combination therapy have been correlated with high expression of *VEGF* receptor gene, and specific inflammation signatures for myeloid cells.^10^ β-catenin activation in HCC can confer resistance to anti-PD-1 monotherapy by T-cell exclusion.^11^,^12^ Of note, β-catenin-related resistance may be overcome by combination with atezolizumab/bevacizumab,^10^ suggesting plasticity in the β-catenin-related HCC resistance to anti-PD-(L)1 when combined with other treatments. Nevertheless, these correlations have not been confirmed as HCC biomarkers for patient stratification to increase therapeutic response.

The influence of the gut microbiome on ICB response has been most studied in melanoma^13^,^14^,^15^,^16^,^17^,^18^,^19^ with fewer explorations in liver cancer.^20^,^21^,^22^,^23^,^24^,^25^,^26^,^27^,^28^ There is still limited consensus on the specific microbiome signatures linked to resistance or sensitivity,^18^,^19^ and some have recently suggested metabolomic signatures as a better prognostic tool than gut microbiota composition.^28^ Poorer ICB responses have been observed in cancer patients treated with antibiotics (ABXs),^13^,^29^,^30^ but these associations are not as clear for patients with HCC yet. Worse ICB therapy outcomes of patients treated with ABXs have been reported,^31^,^32^,^33^ while other studies have reported no significant impact of ABXs.^34^,^35^ Diverse mechanisms of modulation of the tumor immune microenvironment and response to ICB by commensal microbes and microbiome-derived products have been described,^13^,^16^,^36^,^37^,^38^,^39^ with early efforts at translation in human fecal microbiome transplant (FMT) trials to overcome ICB resistance in melanoma.^40^,^41^,^42^ Ongoing clinical trials in patients with HCC (NCT05690048 and NCT05750030) aim to explore the clinical effects of FMT in patients with poor response to ICB. As research on the gut microbiome’s role in melanoma and its clinical relevance progresses, these findings cannot be directly translated to HCC. Melanoma and liver cancer differ considerably in terms of risk factors, tumor microenvironment, and their physiological and anatomical connections with the gut microbiome. To advance microbiome-based clinical approaches for HCC, we need effective models that mimic the complex interaction between liver cancer and the gut microbiome and utilize them to integrate clinical findings from patients.

Here, we performed 16S rRNA sequencing of cross-cohort stool samples from 10 patients with HCC who were responders (R) and 40 non-responders (NR) to ICB. A total of 81 microbiomes were used for analysis, which included stool samples from baseline (n=47) and on-treatment time points (n=34), with mostly paired samples. Minimal microbiome changes occurred upon ICB treatment. We found enrichment of *Bacteroidetes* in the gut microbiome of patients who were NR compared with R. Using optimized in vivo modeling tools, we observed that our immunogenic mouse model of HCC presented worse outcomes following ABX-induced dysbiosis and experienced changes in the tumor immune microenvironment composition. The pro-tumorigenic influence of *Bacteroidetes* was confirmed by FMT with microbiomes enriched in *Bacteroidetes* or with specific *Bacteroidetes* species. Finally, we demonstrate that FMT with *Bacteroidetes* from a human donor was sufficient to impair the response to anti-PD-L1 in our immunogenic mouse model of HCC.

## METHODS

### Patient’s samples

Fecal samples were obtained from patients with HCC receiving ICB treatment at Mount Sinai Hospital (New York, NY) as SOC or in the context of a perioperative clinical trial, after obtaining informed consent in accordance with a protocol reviewed and approved by the Institutional Review Board at the Icahn School of Medicine at Mount Sinai. Additional information regarding patient samples can be found in Supplemental Material, https://links.lww.com/HC9/C515.

### Mice

Specific pathogen-free (SPF) C57BL/6 wild-type mice of 4–6-week-old were purchased from Envigo. Germ-free (GF) C57BL/6 wild-type mice aged 4–6 weeks were bred in our germ-free facility at Icahn School of Medicine at Mount Sinai Hospital. Hydrodynamic tail-vein injections (HDTVI) were performed in 6-week-old mice. We used females for all the SPF mouse experiments, while both sexes (male/females) were used in our in-breed GF experiments. All mouse experiments were approved by the ISMMS Animal Care and Use Committee (protocol number IACUC-2014-0229). Mice were maintained under SPF conditions or in a GF environment, respectively. Food (Chow diet from PicoLab Rodent Diet 20 #5053, LabDiet) and water were provided ad libitum, and all animals were examined before the initiation of the experiments to ensure that they were healthy. For survival experiments, 120 days and 200 days post-HDTVI were defined as the experiment endpoint for SPF and GF mice, respectively. For time-point experiments, mice were sacrificed 3 weeks post-HDTVI. Upon sacrifice of the mice with liver tumors, stool and liver were collected for either fast freeze, formalin-fixed and paraffin-embedded, or flow cytometry.

### Human and mouse stool samples and single-strain preparation for oral gavage

Approximately 400 mg of pulverized stool was blended into a fecal slurry under anerobic conditions. Stool was passed twice through sterile 100 μm strainers for debris removal and diluted 1:20 in LYBHIV4 media with a final concentration of 15% glycerol, as previously described.^43^ Fecal slurries were stored at −80 °C until needed for oral gavage. Individual species were isolated on agar plates and subsequently grown in LYHBHIv4 media as previously described.^44^ Individual species were sequenced as described.^43^

### Fecal microbiota transplant

Fecal microbiota transplant (FMT) was performed in 4–5-week-old GF mice or SPF mice. In the case of SPF mice, pre-depletion with ABXs ad libitum for 1 week was performed when mice were around 4 weeks old. The concentration used was 1 g/L ampicillin (Sigma-Aldrich), 0.5 g/L neomycin (Sigma-Aldrich), 0.5 g/L vancomycin (Chem-Impex), and 1 g/L metronidazole (Sigma-Aldrich). Mice were then colonized with 0.2 mL of the corresponding blended stool (Ctrl-stool; Vanco-stool, 1014-stool, or 1025-stool) by oral gavage. One to two weeks post-FMT, mice were injected with the plasmids by HDTVI for HCC generation. All FMT experiments were performed for survival experiments.

### Statistical analysis

Descriptive statistics were indicated as counts (n) and proportions (%). Data are represented in the graphs either as median ± interquartile range (IQR) (human analyses) or as mean ± standard deviation (SD) (in vivo studies). Group size was determined based on previous experiments. Statistical significance was analyzed using the Mann–Whitney *U* test (when n<10 or non-normal distribution), the Student's *t* test (when n>10 and normal distribution), or the Wilcoxon test (for paired comparisons). Statistics of multi-group comparisons (more than 2 groups) were established using the ANOVA test. For multiple comparisons, Benjamini–Hochberg (Student *t* test) or Bonferroni (Mantel–Cox test) corrections were used. For microbiome analysis, the unpaired, nonparametric Mann–Whitney U test was used to compare ranks. For β-diversity analysis (Bray–Curtis dissimilarity and PCoA plots), adonis was utilized to perform analysis of variance using distance matrices [ANOVA analysis of variance, Pr(>F)]. For α-diversity analysis, the Shannon diversity index method was used. “MaAsLin2 (Microbiome Multivariable Association with Linear Models)” R package was used to determine multivariable association between clinical metadata and microbial meta-omics features. Differences in survival were calculated using the Kaplan–Meier method and calculated using the log-rank Mantle–Cox test. R 4.0.5. (R Foundation for Statistical Computing) and Prism 10 (GraphPad Software Inc., CA, USA) were used to create the graphs and to perform the statistical analysis. Significance values were set at *p<0.05, **p<0.01, ***p<0.001, and ****p<0.0001; ns, not significant.

Remaining methods can be found in Supplemental Material, https://links.lww.com/HC9/C515.

## RESULTS

### Distinct gut microbiome signatures are associated with response to ICB in patients with HCC

To better understand the gut microbiome’s role in the response of HCC to ICB, we analyzed 16S rRNA amplicon sequencing of 81 stool samples from 50 patients with HCC across 3 cohorts that were treated with anti-PD-1 or anti-PD-L1, alone or in combination with other therapeutics. Cohort A included 19 subjects and 30 samples from a phase 2a (ph2a) clinical trial of perioperative cemiplimab (anti-PD-1) in patients with early-stage resectable HCC, while cohort B included 14 subjects and 20 samples from a ph2a clinical trial of perioperative nivolumab alone (n=5) or in combination with a CCR2/5 inhibitor (n=4) or anti-IL8 (n=5). Cohort C included 17 subjects and 31 samples from a prospective observational study of patients with HCC with advanced disease being treated with SOC ICB including nivolumab (n=6), pembrolizumab (n=1), atezolizumab/bevacizumab (n=9), or lenvatinib/pembrolizumab (n=1) (Table 1). While cohorts A and B involved patients with early-stage resectable HCC (stages Ib, II, and IIIb), patients in cohort C presented with advanced-stage, unresectable HCC. Patient enrollment and stool sample collection diagram (pre-treatment “V1”, on-treatment “V2”) are summarized in Figure 1A. Paired pre-treatment and on-treatment (V1/V2) samples from 31 patients, 16 unpaired pre-treatment (V1) samples, and 3 unpaired on-treatment (V2) samples were collected. Tumor response assessment was performed by radiographic assay (MRI or CT scan) per RECIST 1.1 criteria, comparing on-ICB treatment to pre-treatment imaging.^45^ For microbiome analyses, patients with partial or complete response were classified as “responders=R” while patients that showed progressive or stable disease were classified as “non-responders=NR.” Similar to larger studies in patients with advanced HCC, there was a 20% ORR with a total of 10 R and 40 NR to ICB (5 R + 14 NR from cohort A, 0 R + 14 NR from cohort B, and 5 R + 12 NR from cohort C). β-diversity, which represents differences in microbial community composition between samples, of the 3 cohorts’ baseline microbiomes (V1) demonstrated non-significant cohort variations in overall microbiome composition (*p*=0.071) (Figure 1B). To gain deeper insights into the temporal dynamics of microbiome changes in patients with HCC with ICB, we conducted a longitudinal analysis across our 31 paired pre-treatment and on-treatment samples (V1/V2). MaAsLin2 analysis showed significant enrichment of only 3 bacterial species on-treatment compared with pre-treatment: *Lactobacillus mucoseae* and 2 “uncultured bacteria” from the *Ruminococcaceae* family (Figure 1C). This indicates minimal gut microbiome changes in patients with HCC upon ICB regimen. We then explored differences in microbiome composition associated with the response to ICB. Analysis of α-diversity, which represents the richness and evenness of microbial species within each sample, showed no significant differences between R and NR when comparing baseline samples from combined cohorts (Figure 2A) or independently from A (Supplemental Figure S1A, https://links.lww.com/HC9/C517) and C (Supplemental Figure S1B, https://links.lww.com/HC9/C517) cohorts (cohort B was excluded as it only included NR patients). No significant differences in β-diversity were found in overall composition when comparing R and NR across the cohorts (*p*=0.0942) (Figure 2B), with cohort differences explaining only 5.18% of variance, which was not significant (*p*=0.139). These results reveal no major differences in overall gut microbiome composition associated with response to ICB.

**TABLE 1:** Patient age, sex, demographics, and response to immunotherapy

	Cohort A	Cohort B	Cohort C
**Age (years),** median (min–max)	68 (46–79)	65 (47–84)	64 (45–77)
Sex			
Female	4 (21.1%)	1 (7.1%)	1 (5.9%)
Male	15 (78.9%)	13 (92.9%)	16 (94.1%)
Race			
African American	1 (5.3%)	2 (14.3%)	1 (5.9%)
Asian	8 (42.1%)	9 (64.3%)	12 (70.6%)
Caucasian	5 (26.3%)	1 (7.1%)	3 (17.6%)
Hispanic/Latino	3 (15.8%)	1 (7.1%)	1 (5.9%)
Other	2 (10.5%)	1 (7.1%)	0 (0%)
Response to ICB			
Responder	5 (26.3%)	0 (0%)	5 (29.4%)
Nonresponder	14 (73.7%)	14 (100%)	12 (70.6%)

Abbreviation: ICB, immune checkpoint blockade.

**FIGURE 1:**
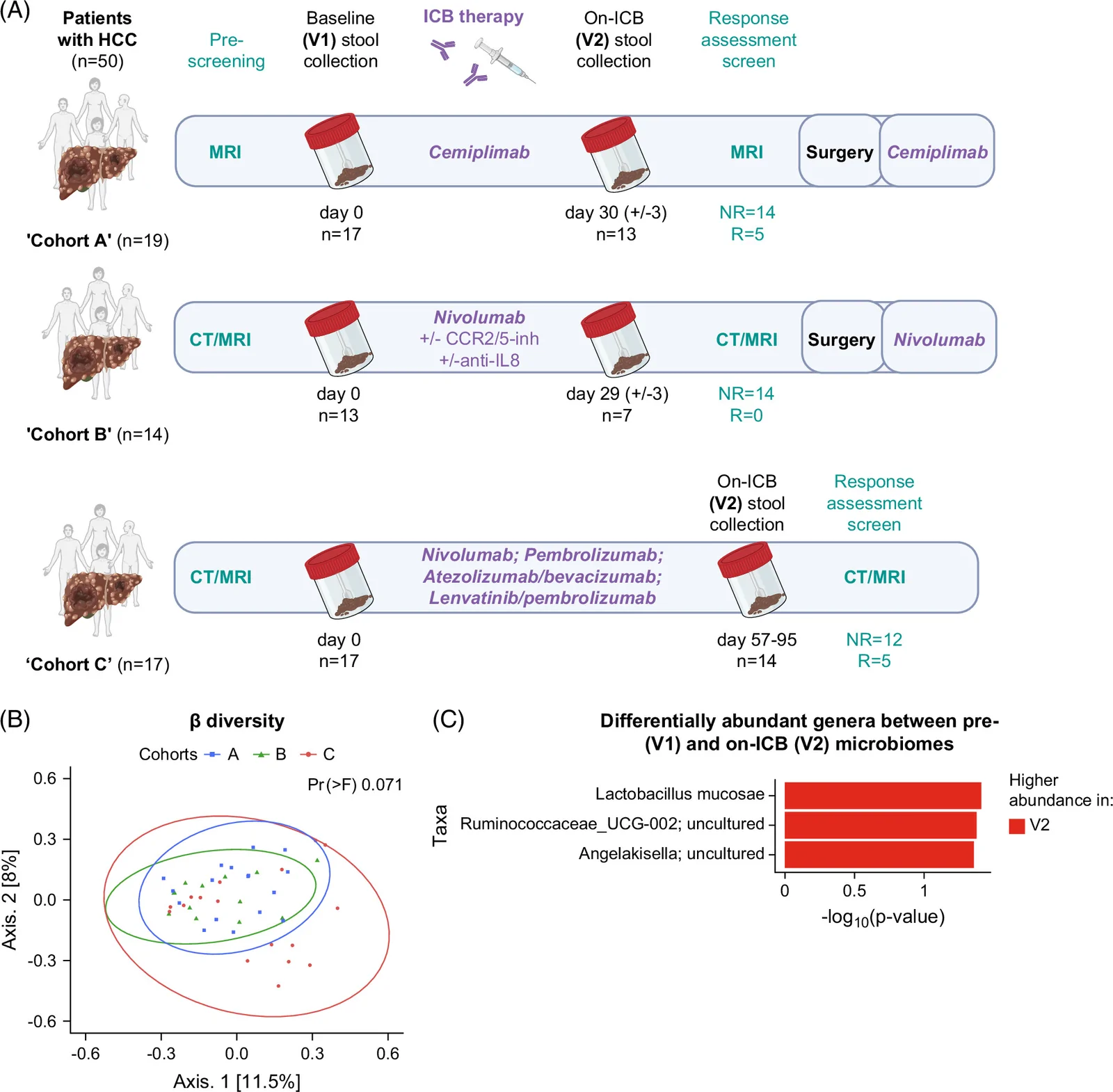
Cross-cohort analysis of patients with HCC reveals minimal longitudinal gut microbiome changes upon ICB treatment initiation. (A) Schematic diagram of the HCC cross-cohort studies from which stool samples were collected. Generated by BioRender software. (B) Bray–Curtis dissimilarity PCoA plot of β-diversity comparing the baseline microbiomes (V1) from (A) cohorts A (blue), B (green), and C (red). (C) Differentially abundant species (MaAsLin2 *p*<0.05) between baseline (V1) and on-treatment (V2) from paired samples of all 3 cohorts combined. Abbreviations: CT, computed tomography; HCC, hepatocellular carcinoma; ICB, immune checkpoint blockade; MRI, magnetic resonance imaging.

**FIGURE 2:**
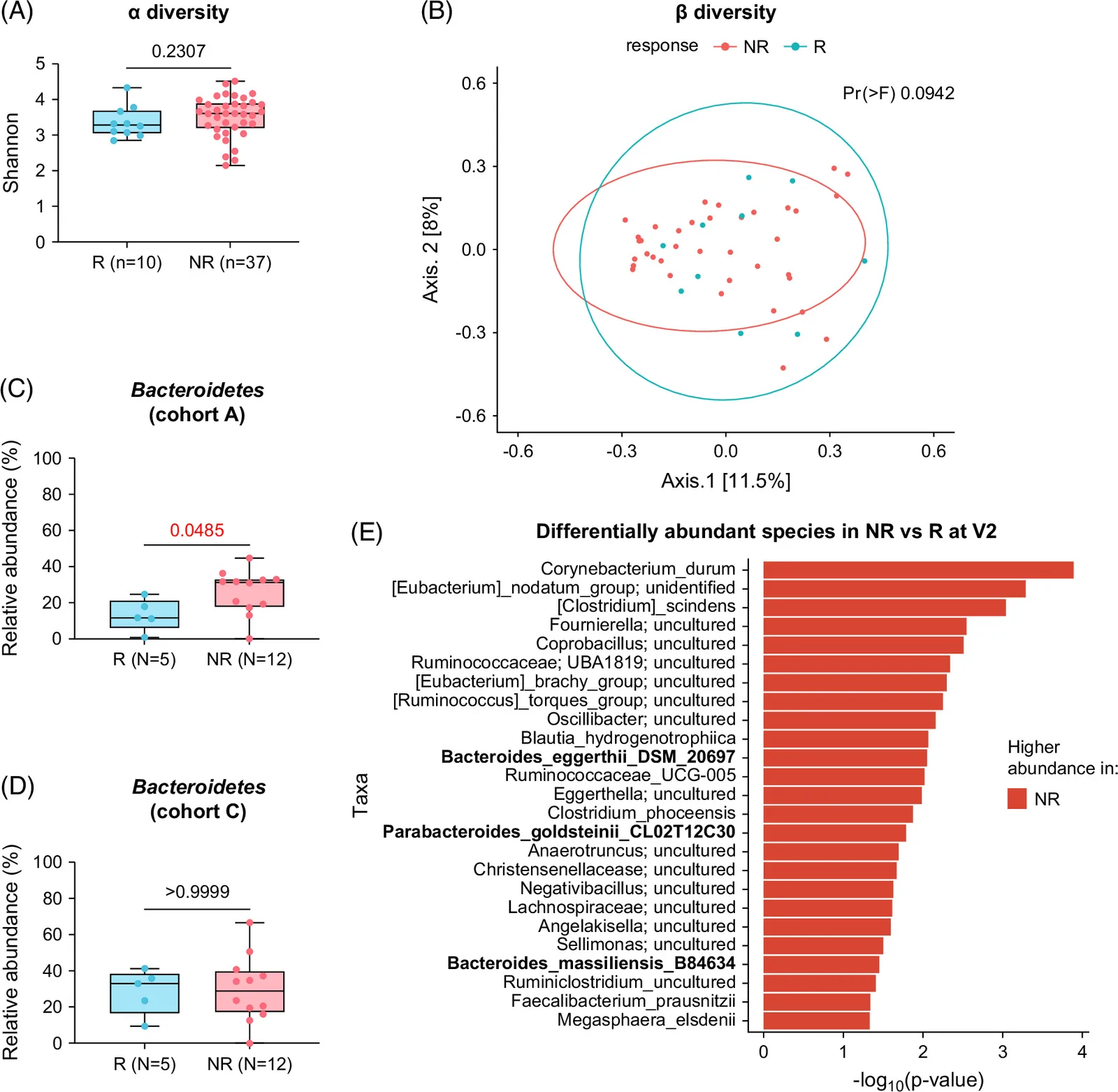
Patients with HCC treated with ICB show differences in gut microbiome composition, with *Bacteroidetes* enrichment in non-responders. (A) α-diversity Shannon plot comparing baseline (V1) stools from R= responders (turquoise) and NR= non-responders (pink) across cohorts A–C. (B) Bray–Curtis dissimilarity PCoA plot of β-diversity comparing the baseline microbiomes (V1) from R and NR across the 3 cohorts. Relative abundance (%) plots of *Bacteroidetes* in R versus NR baseline (V1) stools from (C) “cohort A” and (D) “cohort C.” (E) MaAsLin2 plot of significant species enrichment (*p*<0.05) from on-treatment V2 samples of R versus NR across cohorts A–C. Abbreviations: HCC, hepatocellular carcinoma; ICB, immune checkpoint blockade; NR, nonresponder; R, responder.

To further explore potential differences in gut microbiome composition at baseline (V1), we compared the taxonomic differences between R and NR from cohorts A and C. *Bacteroidetes* phylum was significantly enriched in the NR group compared with R in cohort A (Figure 2C) (*p=0.0485), but not in cohort C (*p*>0.9999) (Figure 2D), suggesting a cohort-dependent NR association of *Bacteroidetes* enrichment at baseline that might be associated with early-stage versus advanced-stage HCC. *Firmicutes*, *Actinobacteria*, *Fusobacteria*, and *Proteobacteria* phyla, which are the other major phyla in humans, showed no difference between R and NR in any of the cohorts (Supplemental Figures S1C–J, https://links.lww.com/HC9/C517). *Verrucomicrobia* and *Lentisphaerae* phyla, which are often detected in human gut microbiotas, were mostly undetectable. To achieve a more comprehensive understanding of the cross-cohort taxonomic differences between R and NR, we performed a MaAsLin2 study of the 3 combined cohorts. We only observed 3 significantly altered species-level differences between R and NR samples pre-ICB (V1) (Supplemental Figure S1K, https://links.lww.com/HC9/C517), whereas we observed differences between NR and R during on-ICB (V2), with 25 species significantly higher in the NR relative to the R group, including 3 species from the *Bacteroidetes* phylum (*Bacteroides eggerthii, Parabacteroides goldsteinii*, and *Bacteroides massiliensis*) (Figure 2E). Together, these results indicate *Bacteroidetes* enrichment as a potential candidate biomarker of poor response to ICB in HCC, with several species enriched in NR after ICB treatment initiation.

### Antibiotics can affect tumor progression and reshape the tumor immune microenvironment in a preclinical model of HCC

To explore the causal influence of the microbiome on HCC, we used a mouse model based on HDTVI of a modified transposon vector expressing the oncogene *MYC* and a highly immunogenic version of luciferase linked with the model antigens SIYRYYGL, SIINFEKL, and OVA323-339 (*pT3-Ef1a-MYC-lucOS*), together with a transposon vector expressing a constitutively active β-catenin (*CTNNB1-N90*) and a vector expressing SB13 transposase (*CMV-SB13*). The resulting *MYC-lucOS;CTNNB1* liver tumors have shown sensitivity to anti-PD-L1 combined with anti-VEGF,^10^ representing an HCC model where differences in survival can be associated with the extent of anti-tumor immune responses promoted by the co-expression of OVA antigens.

To initially address whether the gut microbiome can directly impact HCC tumor progression in our immunogenic mouse model of HCC, we tested the effect of 4 different ABXs (ampicillin, vancomycin, neomycin, and metronidazole) on *MYC-lucOS;CTNNB1* tumor-bearing mice. ABX-derived effects on tumorigenesis were assessed after 3 weeks of treatment post-HDTVI (Figure 3A). Counts of macroscopic tumors and histological analysis of livers revealed a significant increase in the number and size of tumors in mice treated with ampicillin or vancomycin, while no differences in tumor burden/size were observed in mice treated with neomycin or metronidazole (Figure 3B and Supplemental Figure S2A, https://links.lww.com/HC9/C517). The liver-to-body weight ratio showed significantly enlarged livers in the vancomycin-treated mice, while no significant differences were found with the rest of the ABXs (Figure 3C). We were particularly intrigued by the vancomycin-induced pro-tumorigenic effect, as this ABX has been previously associated with anti-tumorigenic effects in liver cancer mouse models.^36^ In a survival experiment, mice harboring *MYC-lucOS;CTNNB1* tumors showed significantly reduced survival when treated with vancomycin (Figure 3D), while there was no effect on survival when treated with neomycin (Supplemental Figure S2B, https://links.lww.com/HC9/C517), confirming that vancomycin has a pro-tumorigenic effect and supporting the detrimental effects of some ABXs observed in patients with HCC.^31^,^32^,^33^ To further explore the mechanism of vancomycin tumor promotion, we performed bulk RNA sequencing of vancomycin-treated and control-treated *MYC-lucOS;CTNNB1* tumors collected at sacrifice from the survival experiment. We illustrated in a volcano plot the top differentially expressed genes, including 76 genes significantly upregulated (FD<1.5, *p*<0.05) and 141 genes significantly downregulated (FD<−1.5, *p*<0.05) in the vancomycin-treated mice (Supplemental Figure S2C, https://links.lww.com/HC9/C517). We performed gene set enrichment analysis (GSEA) of differentially expressed genes in vancomycin-treated mice (Supplemental Table S1, https://links.lww.com/HC9/C516, filtered by FDR *q*-value <0.25). This analysis revealed significant enrichment of pathways related to DNA replication/repair, cell-cycle progression, cholesterol biosynthesis, and metabolic reprogramming in vancomycin-treated tumors, together with significant alterations in immunologic signature gene sets (GSE datasets), suggesting that vancomycin promotes tumor proliferation while reshaping the tumor immune microenvironment.

**FIGURE 3:**
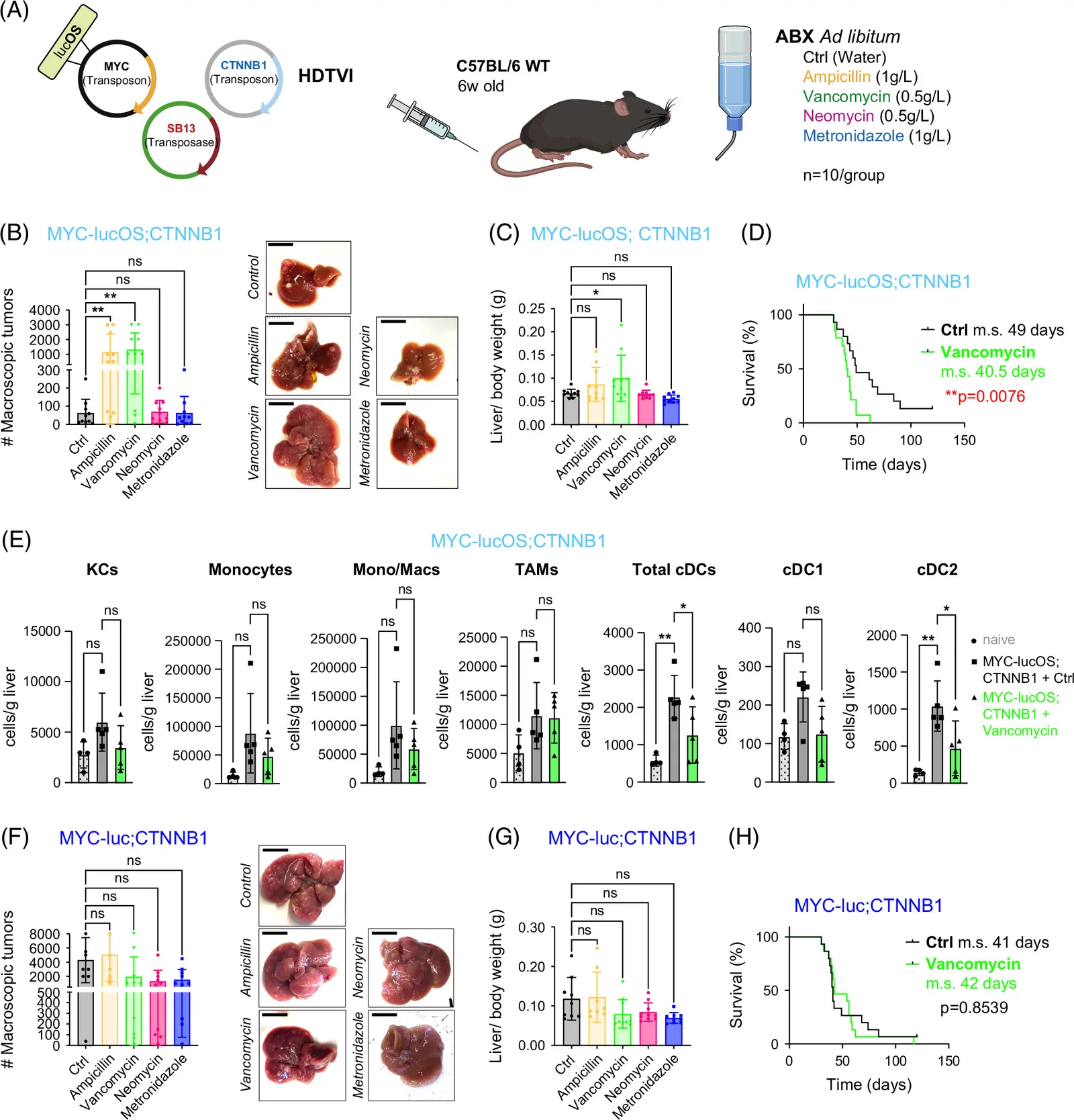
ABXs can promote HCC in vivo and reshape the myeloid liver tumor immune microenvironment in tumors presenting OVA model antigens. (A) Schematic of the experimental design of in vivo ABXs treatment experiments performed in C57BL/6 WT mice. Generated by BioRender. (B) Quantification of *MYC-lucOS;CTNNB1* macroscopic tumors in the livers of the 5 indicated treatment groups at 3 weeks. In the right panel, representative pictures of the mouse livers. Scale bars, 1 cm. (C) Liver-to-body weight ratio of the 5 treatment groups. (D) Survival curve in mice harboring *MYC-lucOS;CTNNB1* tumors treated with vancomycin or without treatment (Ctrl). (E) Quantification of the number of myeloid subpopulations including Kupffer cells (KCs), monocytes, Mono/Macs, tumor-associated macrophages (TAMs), total dendritic cells (DCs), cDC1, and cDC2 in the livers of the corresponding mice (n=4–5 per group). (F) Quantification of *MYC-luc;CTNNB1* macroscopic tumors in the livers of the 5 indicated treatment groups at 3 weeks. In the right panel, representative pictures of the mouse livers. Scale bars, 1 cm. (G) Liver-to-body weight ratio of the 5 treatment groups. (H) Survival curve in mice harboring *MYC-luc;CTNNB1* tumors treated with vancomycin or without treatment (Ctrl). Abbreviations: ABXs, antibiotics; DCs, dendritic cells; HCC, hepatocellular carcinoma; HDTVI, hydrodynamic tail-vein injection; KCs, Kupffer cells; Mono/Mac, monocyte/macrophage; OVA, ovalbumin; TAMS, tumor-associated macrophages; WT, wild type.

These observations suggested that remodeling of the tumor immune microenvironment may represent a key mechanism through which the gut microbiota influences HCC progression in our model. To investigate this possibility, we performed flow cytometric profiling of *MYC-lucOS;CTNNB1* tumor-bearing mice following 3 weeks of vancomycin treatment. Antibiotic-induced dysbiosis resulted in relatively modest changes in neutrophils and the lymphoid compartment (Supplemental Figures S2D, E, https://links.lww.com/HC9/C517) but significantly reduced the abundance of dendritic cells (DCs), particularly cDC2s, within the tumor microenvironment (Figure 3E). In addition, we observed trends toward reduced frequencies of Kupffer cells (KCs), monocytes, and monocyte/macrophage populations, although these differences did not reach statistical significance (Figure 3E). Given the central role of DCs in antigen presentation and the initiation of anti-tumor immune responses, we hypothesized that microbiota-dependent tumor control may be mediated, at least in part, through impaired myeloid cell–driven immune surveillance and T-cell priming.

To further evaluate whether this phenotype depended on anti-tumor immune recognition, we examined the effects of antibiotic treatment in other genetically identical HCC models lacking OVA antigens, *MYC-luc;CTNNB1*. In contrast to the high immunogenic model, antibiotic-induced dysbiosis did not affect tumor progression in OVA-negative tumors (Figures 4F–H), supporting the concept that microbiota-mediated tumor influence requires effective immune recognition of tumor antigens.

**FIGURE 4:**
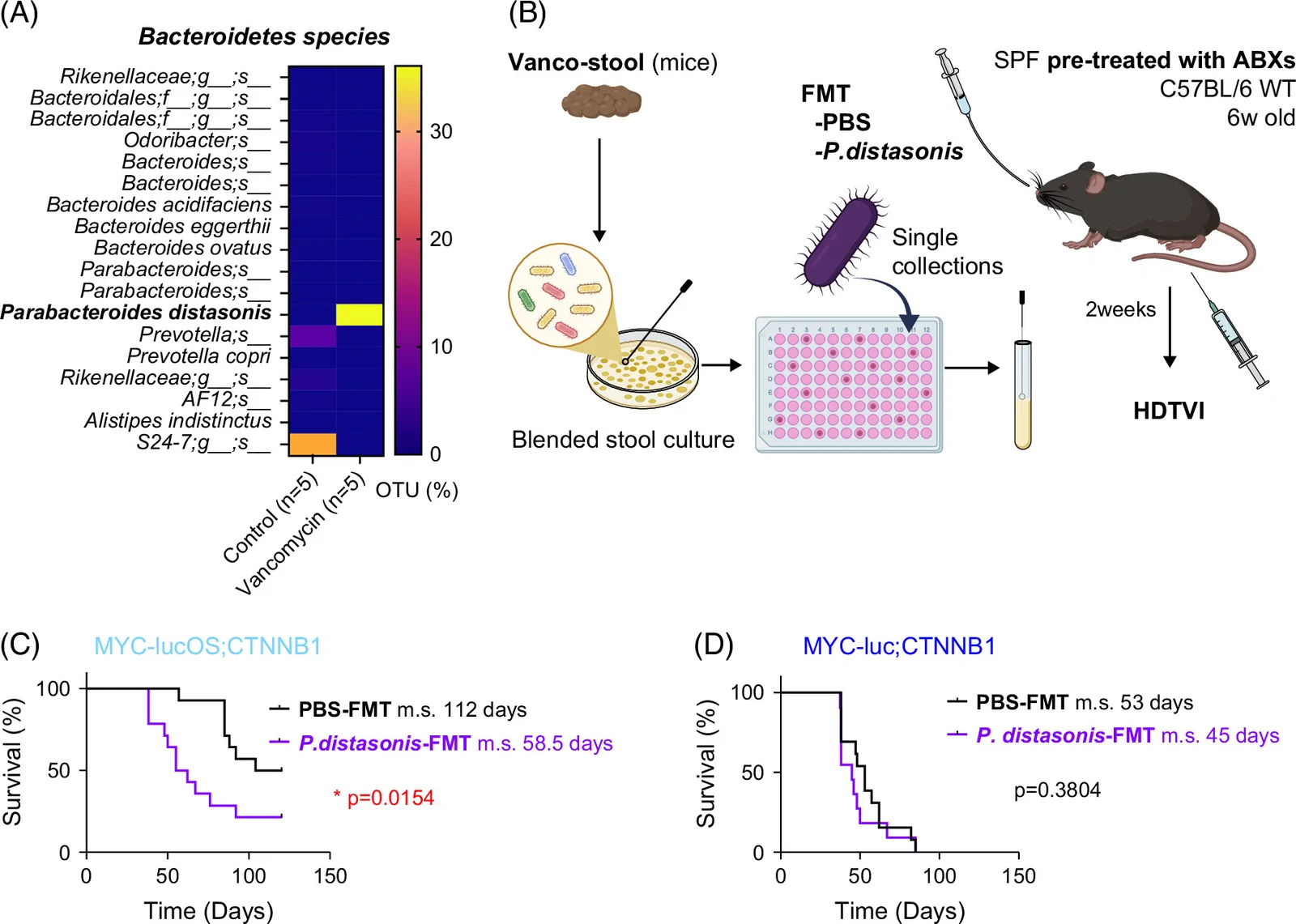
*Parabacteroides distasonis* promotes HCC in mice harboring tumors with OVA model antigens. (A) Heatmap plot of average % OTU *Bacteroidetes* species in ctrl and vancomycin-treated mice stools. (B) Schematic of the experimental design of the in vivo colonization with *P. distasonis* experiment performed in C57BL/6 WT mice. Generated by BioRender. Survival curve in SPF mice pre-treated with ABXs, colonized with *P. distasonis* or PBS (Ctrl-group) by oral gavage and harboring *MYC-lucOS;CTNNB1* (C) or *MYC-luc;CTNNB1* (D) tumors. Abbreviations: ABXs, antibiotics; FMT, fecal microbiome transplant; HCC, hepatocellular carcinoma; HDTVI, hydrodynamic tail-vein injection; OTU, operational taxonomic unit; OVA, ovalbumin; PBS, phosphate-buffered saline; PBS-FMT, phosphate-buffered saline–fecal microbiome transplantation; SPF, specific pathogen-free; WT, wild type.

### Antibiotic-induced gut microbiota alterations, including expansion of *Parabacteroides distasonis*, promote HCC progression in vivo

To confirm that ABX-induced changes in gut microbiome composition influence tumor progression, we first performed 16S rRNA amplicon sequencing from the 4 ABXs experiment in *MYC-lucOS;CTNNB1* tumors shown in Figures 3A, B. We observed that α-diversity was maintained in stool from control mice harboring HCC tumors when compared with stool collected at *T*=0 (before HCC) (Supplemental Figure S3A, https://links.lww.com/HC9/C517). While the α-diversity was significantly reduced in stools from ampicillin-treated, vancomycin-treated, and metronidazole-treated tumor-bearing mice, there was no significant difference in stools from neomycin-treated mice compared with the control group (Supplemental Figure S3A, https://links.lww.com/HC9/C517). The microbial depletion induced by ABXs is highly variable, and it was previously reported that neomycin has lower depletion ability than ampicillin or vancomycin.^46^ We next performed β-diversity analysis and observed a clear separation of the samples by treatment group: ampicillin-treated, vancomycin-treated, and metronidazole-treated samples clustered separately, while T0, control, and neomycin-treated samples clustered together (Supplemental Figure S3B, https://links.lww.com/HC9/C517). These findings could explain the lack of impact of neomycin on *MYC-lucOS;CTNNB1* tumor development. By contrast, metronidazole treatment demonstrated efficacy in inducing gut microbiome dysbiosis. We hypothesize that changes in microbiome composition specifically induced by ampicillin and vancomycin were responsible for the pro-tumorigenic effects in our model. We analyzed the composition of all groups at the phylum level, showing major changes in the abundance of most detected bacterial phyla in the fecal microbiome of vancomycin-treated, ampicillin-treated, and metronidazole-treated mice (Supplemental Figure S3C, https://links.lww.com/HC9/C517). Given that our human HCC analyses showed an increased relative abundance of *Bacteroidetes* in NR to ICB, we explored whether there was any correlation between *Bacteroidetes* composition and the observed pro-tumorigenic effects of vancomycin in mice. In all, 18 species belonging to the *Bacteroidetes* phylum were detected in the murine stools. In the no-antibiotic control group, a species from the *S24-7* family was the most abundant (19%–34% of operational taxonomic units, OUT), followed by *P* *revotella* spp. (4–7 OTU%) while the rest remained at lower percentage (<1 OTU%) (Figure 4A and Supplemental Figure S3D, https://links.lww.com/HC9/C517). In the vancomycin-treated mice, only 2 of the species were detected: *Prevotella copri* (<1 OTU%) and *Parabacteroides distasonis*. *P. distasonis* was not detected in the control group, but greatly expanded in the vancomycin group, representing a large percentage of the microbiome population in these mice (27–43 OTU%) (Figure 4A). We found that ampicillin-treated mice, which also prompted tumor development in our HCC model, similarly showed a great enrichment in *P. distasonis* (69–85 OTU%), unlike neomycin-treated and metronidazole-treated groups (<1 OTU%) (Supplemental Figure S3D, https://links.lww.com/HC9/C517).

To confirm that the pro-tumorigenic effect of vancomycin was directly dependent on gut microbiome dysbiosis, we generated blended stool aliquots of microbiomes from mice that were subjected to vancomycin for 3 weeks (Vanco-stool), in parallel with corresponding control stool aliquots from untreated mice (Ctrl-stool). We used these microbiomes to colonize GF mice by oral FMT before HDTVI with *MYC-lucOS;CTNNB1* (Supplemental Figure S4A, https://links.lww.com/HC9/C517). We observed a greater number of tumor-bearing mice that died within 200 days post-HDTVI in the group of “Vanco-SL”-FMT compared with “Ctrl-SL”-FMT (Supplemental Figure S4B, https://links.lww.com/HC9/C517), although no statistical significance was achieved. Furthermore, we cultured the Vanco-stool and isolated *P. distasonis* to test the impact of colonizing mice with this strain (Figure 4B). When we colonized SPF mice pre-depleted with ABXs, we observed a significantly shorter median survival in mice colonized with *P. distasonis* compared with PBS control (Figure 4C). To validate our previous hypothesis that microbiota-mediated tumor influence requires effective immune recognition of tumor antigens, we tested the effects of *P. distasonis* colonization in the *MYC-luc;CTNNB1* tumor model. As expected, we did not observe any change in tumor progression in colonized mice harboring these tumors without OVA antigens (Figure 4D).

Together, we observed that vancomycin and ampicillin can promote the expansion of specific *Bacteroidetes* species in treated mice, and that *P. distasonis* colonization recapitulates the pro-tumorigenic effect observed with ABXs treatment in a manner dependent on effective immune recognition of tumor antigens. Collectively, these results demonstrate a causal link between ABXs-induced gut dysbiosis and HCC progression in vivo and identify the enrichment of specific *Bacteroidetes* species, including *P. distasonis*, as a key mediator of this effect.

### Gut microbiota composition of patients with HCC can be transferred to mice and significantly influences tumor progression in vivo

We investigated whether differential *Bacteroidetes* relative abundance in the baseline gut microbiome of patients with HCC would affect HCC development in our in vivo experimental system. We selected pre-treatment stools from one NR and one R patient from cohort A that presented a large difference in the abundance of *Bacteroidetes* at phylum level, accounting for 44.70% in NR-subject 1025 (“high*-Bacteroidetes”* microbiota) and 0.89% in R-subject 1014 (“low-*Bacteroidetes”* microbiota) (Figure 5A). This recapitulated the clinical association of *Bacteroidetes* expansion in NR patients with ICB from our cross-cohort study. Within the species detected in these microbiomes (Supplemental Tables S2 and S3, https://links.lww.com/HC9/C516), we identified 24 and 6 species from the *Bacteroidetes* phylum in patients 1025 and 1014, respectively (Supplemental Tables S2 and S3, https://links.lww.com/HC9/C516).

**FIGURE 5:**
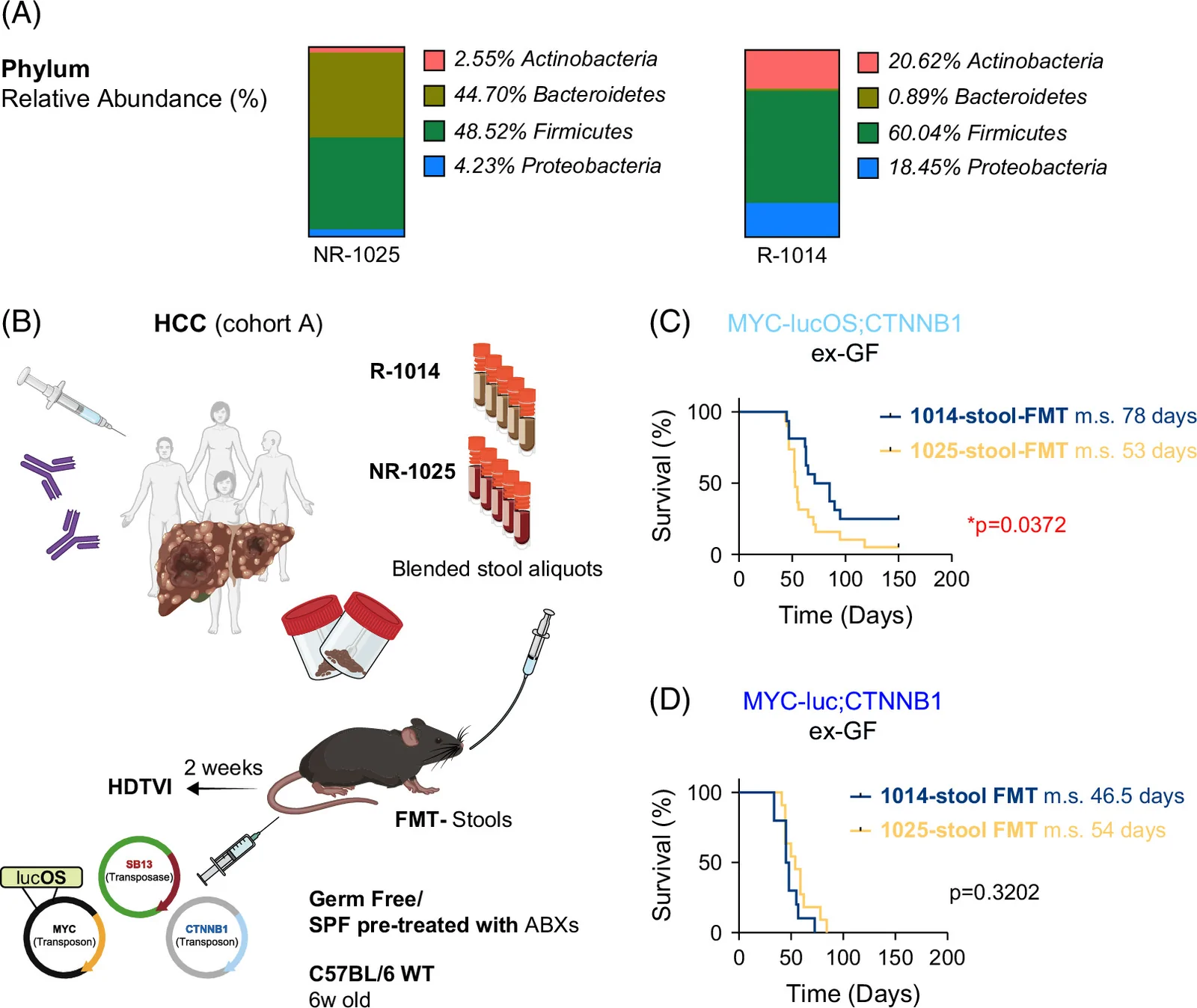
Fecal microbiome transplant with human microbiota enriched in *Bacteroidetes* promotes HCC in mice harboring tumors with OVA model antigens. (A) Taxonomic plots of phylum relative abundance (%) composition from patients’ stools collected from NR-1025 (left) and R-1014 (right) subjects from HCC “cohort A.” (B) Schematic of experiment design of survival experiments on either C57BL6 WT GF or SPF mice pre-depleted with ABXs harboring *MYC-lucOS;CTNNB1* tumors after FMT with blended stool generated from an R-donor (1014 subject) and an NR-donor (1025 subject). Schematic generated by BioRender. Survival curves from ex-GF mice harboring *MYC-lucOS;CTNNB1* (C) or *MYC-luc;CTNNB1* (D) tumors upon FMT with 1025-stool or 1014-stool. Abbreviations: ABXs, antibiotics; FMT, fecal microbiome transplant; GF, germ-free; HCC, hepatocellular carcinoma; HDTVI, hydrodynamic tail-vein injection; OVA, ovalbumin; SPF, specific pathogen-free; WT, wild type.

GF mice were colonized with fecal slurries from the relevant human donor before HDTVI of *MYC-lucOS;CTNNB1* (Figure 5B). We found that ex-GF mice from 1025-FMT exhibited a significantly reduced median survival when compared with 1014-FMT mice (Figure 5C). We validated these results in SPF mice pre-depleted with ABXs before FMT, which reproduced the prolonged median survival in 1014-FMT colonized mice compared with 1025-FMT (Supplemental Figure S5A, https://links.lww.com/HC9/C517). Furthermore, the different effect on tumor progression with 1014-FMT versus 1025-FMT was abrogated in *MYC-luc;CTNNB1* HCC tumors lacking OVA antigen recognition, as no differences in survival were observed in this mouse model (Figure 5D). These findings suggest that microbiome-driven modulation of tumor progression upon human microbiome FMT depends on antigen-specific immune responses.

### *Bacteroidetes* enrichment significantly influences tumor progression and impairs ICB response in vivo

To explore the causative role of *Bacteroidetes* enrichment in tumor promotion in our HCC model, we isolated species from 1025 stool samples and created a cocktail of 6 *Bacteroidetes* species: *Bacteroides caccae* (*B. caccae*), *Bacteroides finegoldii* (*B. finegoldii*), *Bacteroides thetaiotaomicron* (*B. thetaiotaomicron*), *Bacteroides uniformis* (*B. uniformis*), *Bacteroides vulgatus* (*B. vulgatus*), and *P. distasonis*. We colonized SPF mice (pre-depleted with ABXs) with this cocktail before HDTVI and followed tumor development and survival of mice harboring *MYC-lucOS;CTNNB1* tumors (Figure 6A).

**FIGURE 6:**
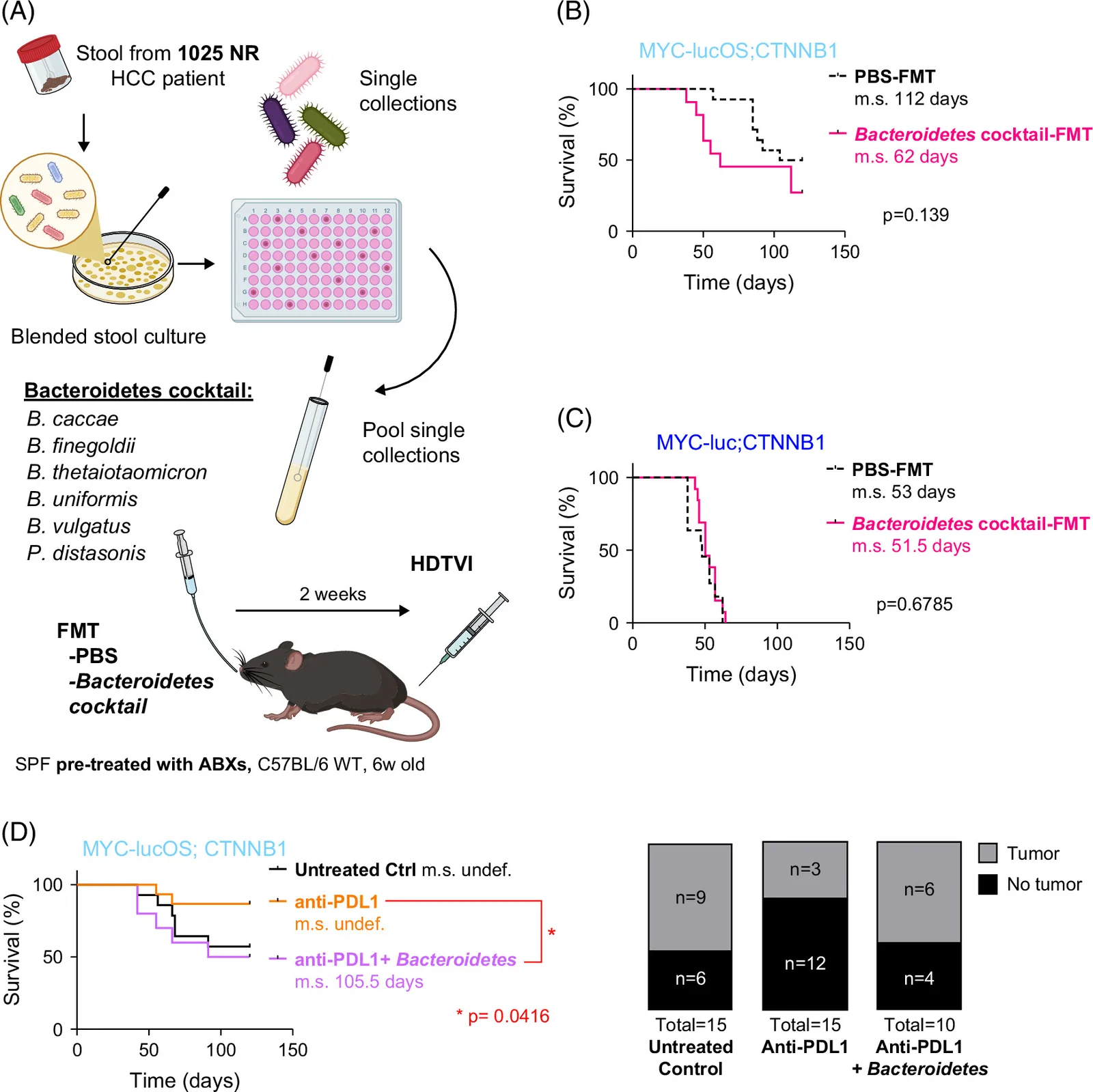
*Bacteroidetes* promote HCC and impair anti-PD-L1 response in mice harboring tumors with OVA model antigens. (A) Schematic of the experimental design of in vivo colonization in SPF mice pre-depleted with ABXs, colonized with a 6-species *Bacteroidetes* cocktail or PBS (Ctrl-group) by oral gavage. Generated by BioRender. Survival curve in SPF mice pre-treated with ABXs, colonized with a 6-strain *Bacteroidetes* cocktail or PBS (Ctrl-group) by oral gavage and harboring *MYC-lucOS;CTNNB1* (B) or *MYC-luc;CTNNB1* (C) tumors. Dot-style line indicates that these results were already included in a previous graph within this manuscript; as the experiments were run together, a unique control group was used to reduce the number of mice to utilize. (D) Survival curve in SPF mice pre-treated with ABXs, colonized with a 6-strain *Bacteroidetes* cocktail or PBS (Ctrl-group) by oral gavage, and harboring *MYC-lucOS;CTNNB1* tumors and treated with anti-PD-L1. Parts of the whole tables on the right, showing tumor prevalence at endpoint (120 d post-HDTVI). Abbreviations: ABXs, antibiotics; FMT, fecal microbiome transplant; HCC, hepatocellular carcinoma; HDTVI, hydrodynamic tail-vein injection; NR, nonresponder; OVA, ovalbumin; PBS, phosphate-buffered saline; PBS-FMT, phosphate-buffered saline–fecal microbiome transplantation; SPF, specific pathogen-free.

*Bacteroidetes*-cocktail FMT was associated with reduced early survival that was significant at 90 days post-HDTVI (median 62 vs. 112 d), although the survival difference did not reach significance (*p*=0.139) at 120 days post-HDTVI. This suggests that the study was underpowered to observe statistical survival differences at 120 days post-HDTVI (Figure 6B). On the other hand, *Bacteroidetes*-cocktail FMT had no impact on *MYC-luc;CTNNB1* tumor progression (Figure 6C). As a control for these experiments, and to validate that the pro-tumorigenic effect of the *Bacteroidetes* cocktail was specific for the selected bacterium, we colonized mice with a cocktail of 6 *Bifidobacterium* species (2 *Bifidobacterium bifidum,* 2 *Bifidobacterium breve*, and 2 *Bifidobacterium longum*) that were previously isolated from other human microbiomes.^47^ In these experiments, we found a non-significant anti-tumoral trend in colonized mice harboring *MYC-lucOS;CTNNB1* compared with the Control PBS-FMT group (Supplemental Figure S5B, https://links.lww.com/HC9/C517), while no significant effect in mice harboring *MYC-luc;CTNNB1* (Supplemental Figure S5C, https://links.lww.com/HC9/C517).

Finally, to determine whether *Bacteroidetes* colonization modulates response to ICB, we assessed survival in the *MYC-lucOS;CTNNB1* model under anti-PD-L1 therapy. Similarly, SPF mice were depleted of their endogenous microbiota with ABXs and then received oral gavage with either a control inoculum (PBS-FMT) or *Bacteroidetes* cocktail (Figure 6A). Tumors were induced by HDTVI and treated with anti-PD-L1. Anti-PD-L1 treatment of PBS-FMT mice showed strong tumor control: only 2 of 15 mice died of tumor-related causes during the study and overall tumor prevalence 3 out of 15 by endpoint (120 d post-HDTV) (Figure 6D). In contrast, untreated control mice exhibited substantially higher disease burden, with 6 out of 15 tumor-related deaths and higher tumor prevalence (9/15). These results indicate that these experimental conditions are sufficient to elicit a detectable anti-PD-L1 response. Colonization with *Bacteroidetes* impaired this response. Compared with anti-PD-L1-treated PBS-FMT mice, anti-PD-L1-treated *Bacteroidetes-*FMT mice showed significantly reduced survival (*p*=0.0416) (Figure 6D), with 5 out of 10 mice in the anti-PD-L1 + *Bacteroidetes* group dying of tumor-related causes and a tumor prevalence of 6 out of 10. These results indicate that *Bacteroidetes* colonization attenuates the protective anti-PD-L1 response in vivo.

Together, mice colonized with human microbiotas with high enrichment in *Bacteroidetes* exhibit a pro-tumor effect and impairment of the anti-PD-L1 response in *MYC-lucOS;CTNNB1* immunogenic HCC tumors, indicating a relevant role of gut *Bacteroidetes* composition in modulating HCC outcome and the anti-tumor immune response. Moreover, these results align with our previous results, which demonstrate that *P. distasonis* alone can promote tumor progression in mice harboring *MYC-lucOS;CTNNB1* tumors.

## DISCUSSION

The gut microbiome’s contribution to tumorigenesis and ICB response in patients with HCC is still poorly understood. Here, we profiled the microbiome composition of patients with advanced HCC undergoing ICB treatment. This study included paired pre-treatment and on-treatment samples from ICB-treated patients with HCC. Longitudinal changes in microbiome composition in ICB-treated patients have been previously reported in large cross-cohorts of melanoma patients^48^ but have been less studied in HCC cohorts.^23^ Our analyses identified only minor changes in the microbiome following ICB, indicating limited temporal dynamics in microbiome composition over the course of treatment.

A major finding from this work is the enrichment of *Bacteroidetes* in patients with HCC who did not respond to ICB. Different types of cancer have previously shown increased *Bacteroidetes* in patients to correlate with poor response to ICB.^14^,^17^,^41^,^42^,^49^ Some *Bacteroidetes* species were recently reported to be associated with response in patients with HCC.^20^,^21^ Poorer response to ICB was associated with higher abundance of *Bacteroides stercoris* ^20^ and *Bacteroides_AF20_13LB* ^21^ in patients with HCC treated with atezolizumab/bevacizumab or anti-PD-1, respectively. Moreover, skewed *Firmicutes/Bacteroidetes* ratio and low *Prevotella/Bacteroides* ratio have been proposed as predictive markers of non-response to nivolumab in advanced HCC.^24^ However, no study has causally demonstrated the role of *Bacteroidetes* in HCC resistance to ICB. Interestingly, we found a cohort-dependent association of *Bacteroidetes* enrichment with non-response. While the difference in R versus NR is clear in our cohort involving patients with earlier-stage HCC, this difference was not significant in our cohort with more advanced unresectable HCC. Future analysis in larger cohorts including patients with different stages of HCC would clarify whether *Bacteroidetes* enrichment is a more accurate predictive biomarker of poor response to ICB in patients at earlier stages of HCC and/or a biomarker for neoadjuvant therapy response exclusively.

The lack of appropriate mouse models to validate gut microbiome influence in liver cancer patients hinders mechanistic studies, while findings in existing models are often not consistently replicated in patients. To address this gap, we have developed genetically-engineered precision mouse models of HCC that mirror tumor genetics and tumor immune characteristics linked to ICB resistance observed in patients,^10^,^11^ including 2 complementary systems: (1) expressing OVA antigens that trigger enhanced immunogenicity and enable antigen-specific immune recognition, and (2) a matched non-OVA counterpart with identical tumor genetics but reduced immunogenicity, representing a “cold” tumor context with limited antigen recognition.

Here, we tested the effects of ABX-induced dysbiosis in HCC progression and validated the findings from human cohorts, which identified putative microbial species that may predispose patients to ICB resistance. The HCC mouse models used in this study, *MYC-lucOS;CTNNB1* and *MYC-luc;CTNNB1*, demonstrated a significant impact on tumor development and survival when mice were treated with specific ABXs (ampicillin and vancomycin) only in the immunogenic context of *MYC-lucOS;CTNNB1* tumors. We observed that vancomycin treatment influences the myeloid liver/tumor compartment by significantly reducing the abundance of DCs, which suggests impaired myeloid cell–driven immune surveillance and T-cell priming. We found that pro-tumorigenic ABXs induce a marked expansion of *P. distasonis*. Upon isolation and colonization experiments, we confirmed its pro-tumoral role in HCC, indicating that the expansion of *P. distasonis* is responsible for the effects observed following vancomycin and ampicillin treatment.

In addition, we colonized mice with representative human microbiomes from our HCC patient cohort to span a range of *Bacteroidetes* enrichment. *Bacteroidetes* enrichment was correlated with faster tumor progression and reduced survival in mice harboring immunogenic *MYC-lucOS;CTNNB1* tumors, in contrast to no effect on tumor progression in the non-immunogenic *MYC-luc;CTNNB1* model—this implicates an immune-dependent mechanism. Consistent with this, colonization of mice with a *Bacteroidetes* cocktail impaired the response to anti-PD-L1 in *MYC-lucOS;CTNNB1* model. Together, these in vivo findings corroborate the association we identified in the clinical cohort between *Bacteroidetes* enrichment and lack of response to ICB. Our study also suggests that limiting ABX use in patients with HCC undergoing ICB may be beneficial for patients’ outcomes, as previously suggested by others.^50^ Based on our results, pre-selection of ABXs that can target *Bacteroidetes* species, or exclusion of ABXs to which *Bacteroidetes* species are known to be resistant, may be a better choice when applicable. On the other hand, development of targeted ABXs capable of reducing *Bacteroidetes* may be exploited therapeutically in preclinical and clinical studies in combination with ICB for patients with HCC.

In conclusion, we identified a differential *Bacteroidetes* signature as a potential biomarker of response to ICB in patients with HCC. Using an immunogenic HCC mouse model, we further established that the *Bacteroidetes* enrichment promotes HCC progression in vivo by impairing anti-tumor immunity and attenuating the response to ICB. Further studies are warranted to investigate the potential benefit of targeting *Bacteroidetes* as adjuvant therapy to enhance ICB response in patients with HCC.

## Supplementary Material
