# Supplementary material for: Bacteroidetes promote hepatocellular carcinoma progression and resistance to immunotherapy
Source: Hepatol Commun. 2026 Nov 11;10(12):e01086. doi: 10.1097/HC9.0000000000001086 (PMC13766593; doi:10.1097/HC9.0000000000001086)
Supplement: Supplementary file 1 [file hc9-10-e01086-s003.pdf]

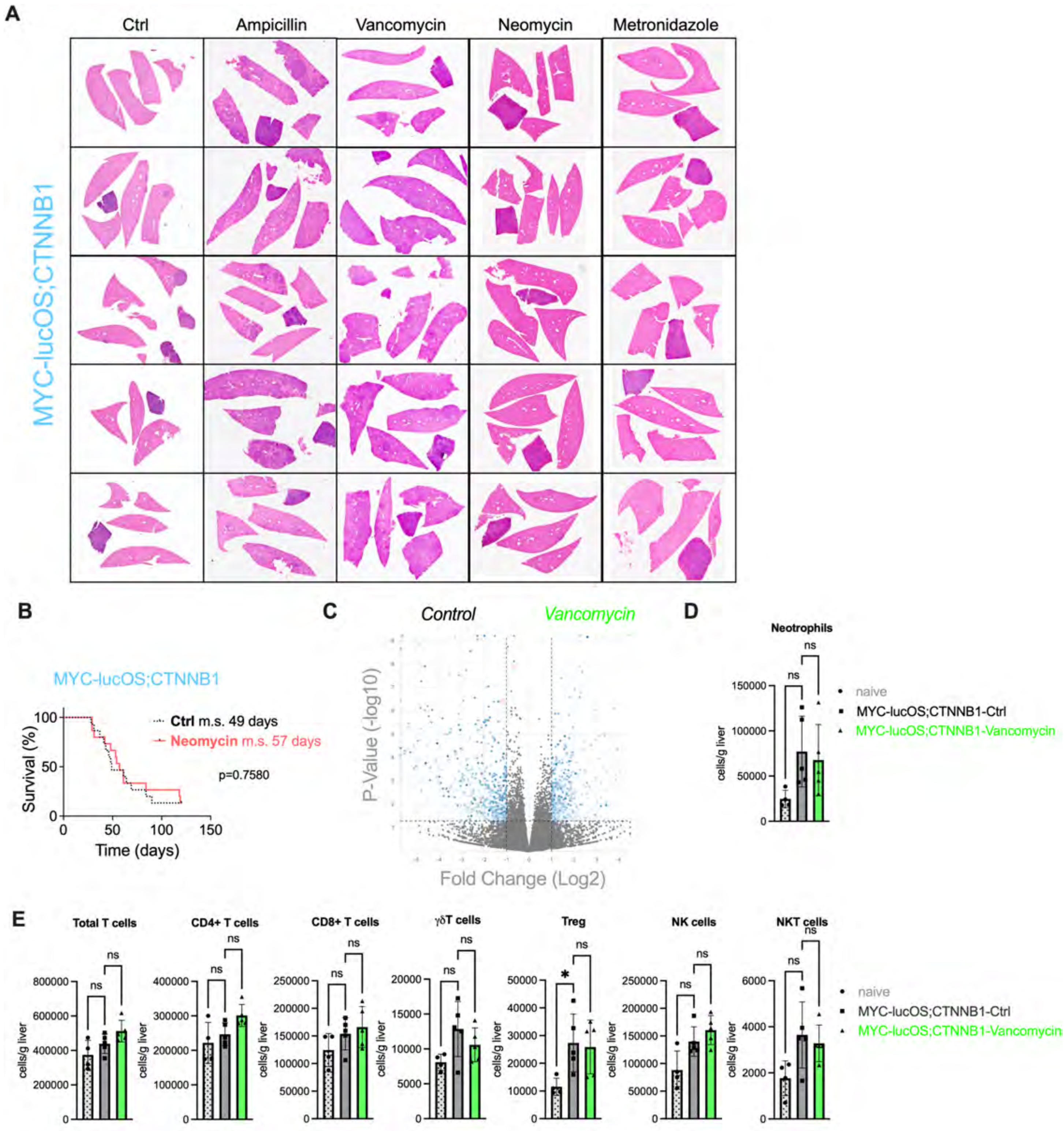


**S2**

**Supplementary figure S2.** A) Representative pictures of H&E stains of 5 murine livers at 3 weeks post HDTVI of *MYC-lucOS;CTNNB1* of 5 different groups from left to right: untreated (Ctrl), ampicillin-treated, vancomycin-treated, neomycin-treated, and metronidazole-treated. B) Survival curve from mice harboring *MYC-lucOS;CTNNB1* tumors treated with neomycin and compared with untreated (Ctrl). Dot-style line indicates that these results were already included in a previous graph within this manuscript, as the experiments were run together, a unique control group was used to reduce the number of mice to utilize. C) Volcano plot of bulk RNA sequencing of murine *MYC-lucOS;CTNNB1* tumors treated with vancomycin or without treatment (Ctrl) in survival experiment. Quantification of the number of Neutrophils (D) and lymphoid subpopulations (E) including Total T cells, CD4+ T cells, CD8+ T cells, γδT cells, Treg cells, NK cells and NKT cells in the livers of the corresponding mice (n = 4–5 per group).

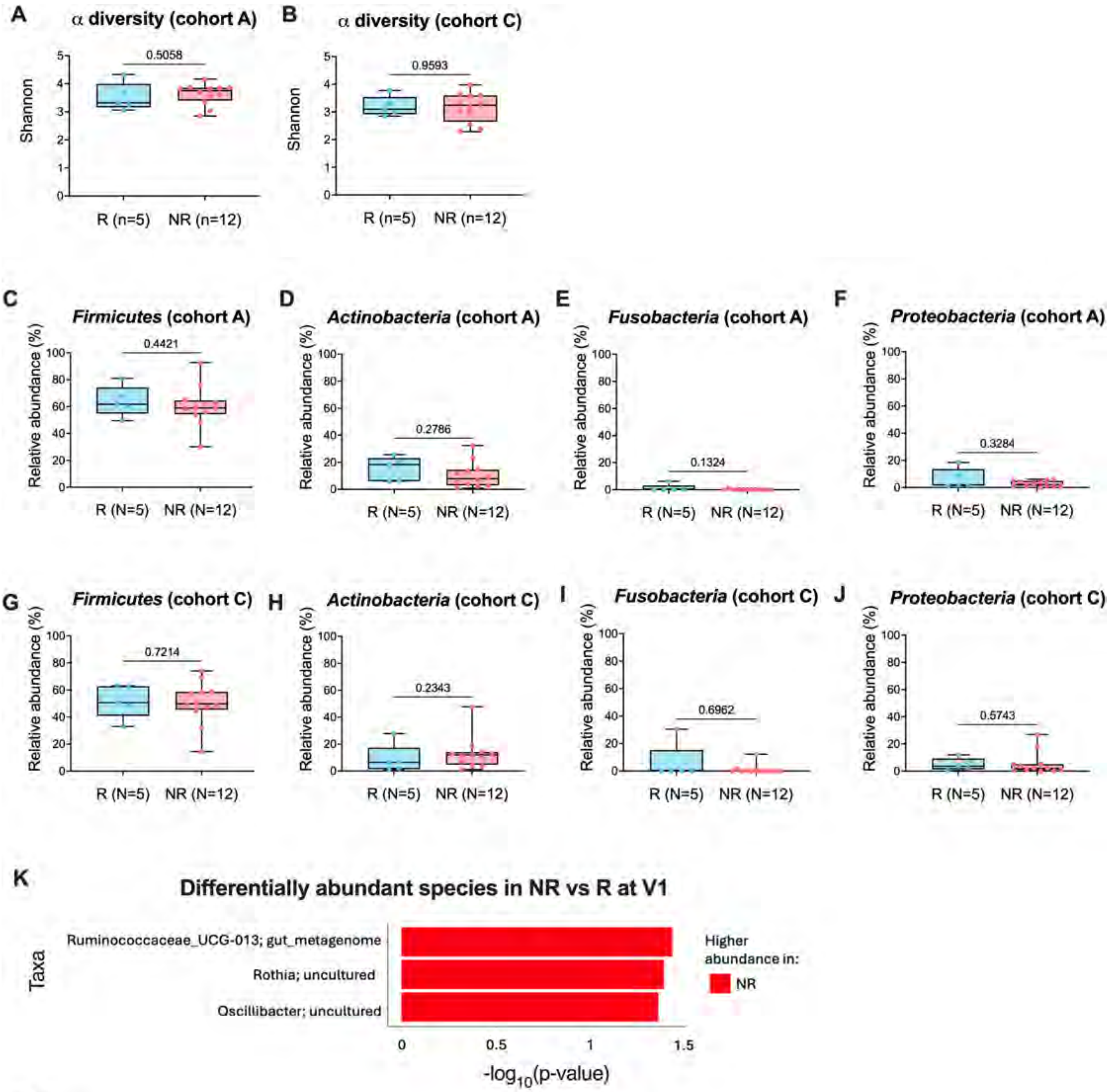


**S1**

**Supplementary figure S1.** α-diversity Shannon plot comparing R and NR baseline (V1) stools from A) cohort A and B) cohort C. Relative abundance (%) phylum plots of C) *Firmicutes,* D) *Actinobacteria,* E) *Fusobacteria,* and F) *Proteobacteria* of R vs NR baseline microbiomes (V1) from cohort A. Relative abundance (%) phylum plots of G) *Firmicutes,* H) *Actinobacteria,* I) *Fusobacteria,* and J) *Proteobacteria* of R vs NR baseline microbiomes (V1) from cohort C. K) Significantly enriched species in NR vs R (MaAsLin2 , $p<0.05$) from V1 samples across cohorts A-C. R= responders (turquoise) and NR= non-responders (pink).

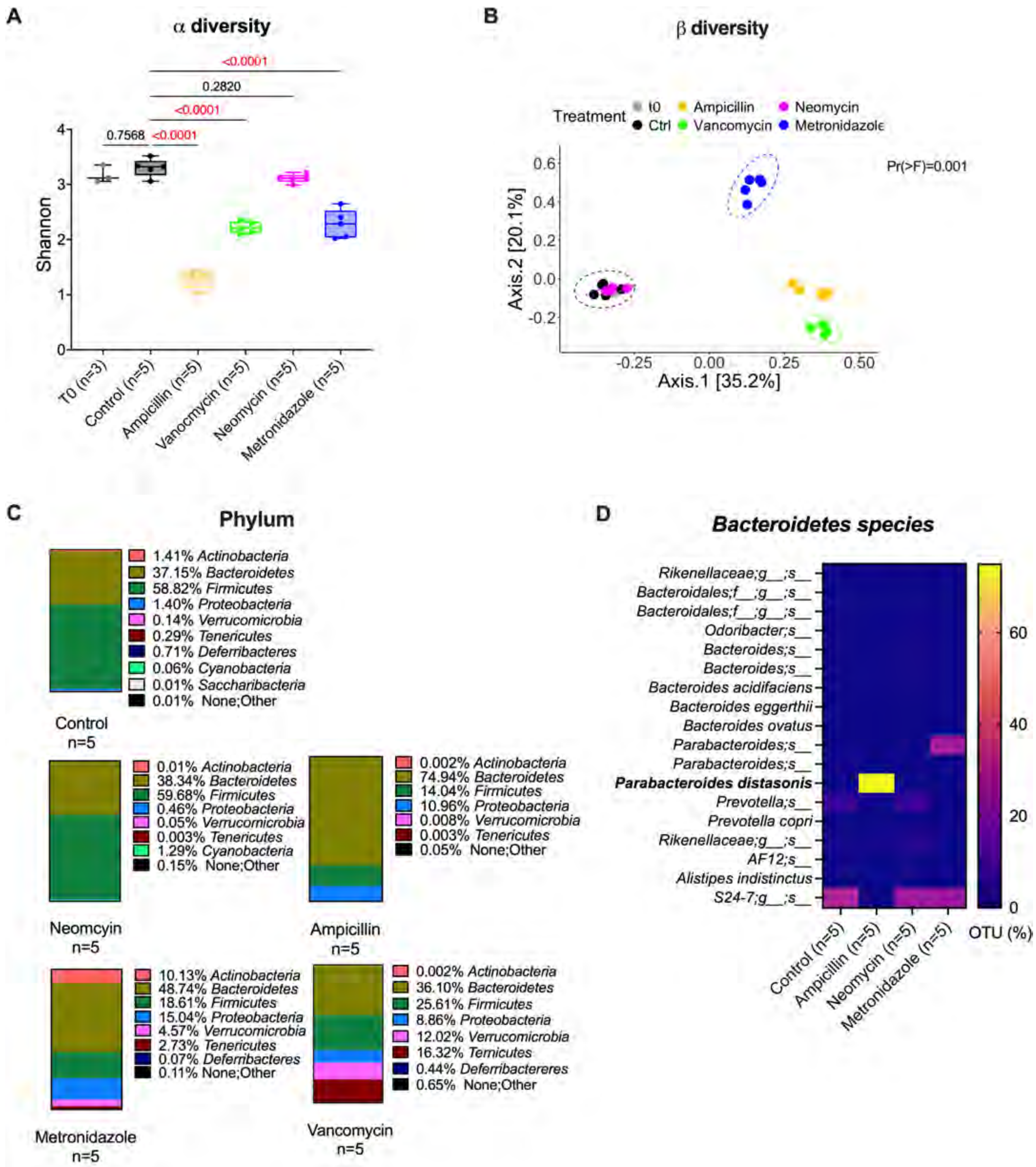


**S3**

**Supplementary Figure 1:** α-diversity Shannon plot comparing the microbiomes from murine stools collected from t0 (grey), ctrl (black), ampicillin (yellow), vancomycin (green), neomycin (magenta) and metronidazole (blue) groups. B) Bray-Curtis dissimilarity PcoA plot of β-diversity comparing the microbiomes from murine stools collected from t0 (grey), ctrl (black), ampicillin (yellow), vancomycin (green), neomycin (magenta) and metronidazole (blue) groups. C) Representative plots of average phylum composition from cecum stools collected from 5 Ctrl (water) mice, 5 vancomycin-treated mice, 5 ampicillin-treated mice, 5 neomycin-treated mice and 5 metronidazole-treated mice for 3 weeks upon *MYC-lucOS;CTNNB1* HDTVI. D) Heatmap plot of average % relative abundance of Bacteroidetes species in stools from ctrl-, ampicillin-, neomycin- and metronidazole-treated mice.

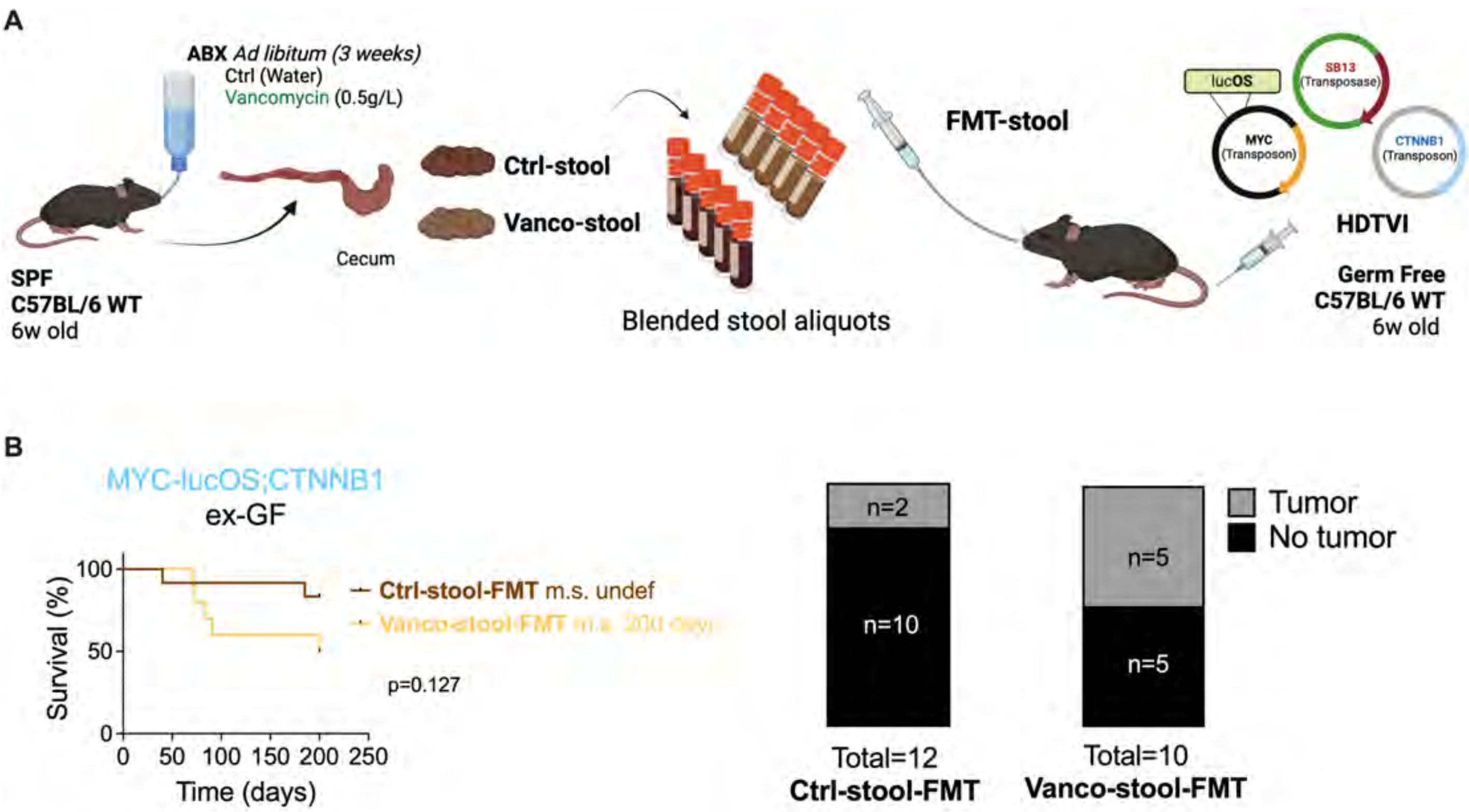


## S4

**Supplementary figure S4.** A) Schematic of experiment design of survival experiment on C57BL6 WT female GF mice harboring *MYC-lucOS;CTNNB1* tumors after FMT with blended stools previously generated from vancomycin-treated mice stools ('Vanco-stool'), and control-treated mice stools ('Ctrl-stool') generated by BioRender. B) Survival curve of ex-GF mice harboring *MYC-lucOS;CTNNB1* tumors upon FMT with 'Vanco-stool' or 'Ctrl-stool'. Parts of whole tables on the right, showing tumor penetrance at endpoint (200 days post-HDTVI).

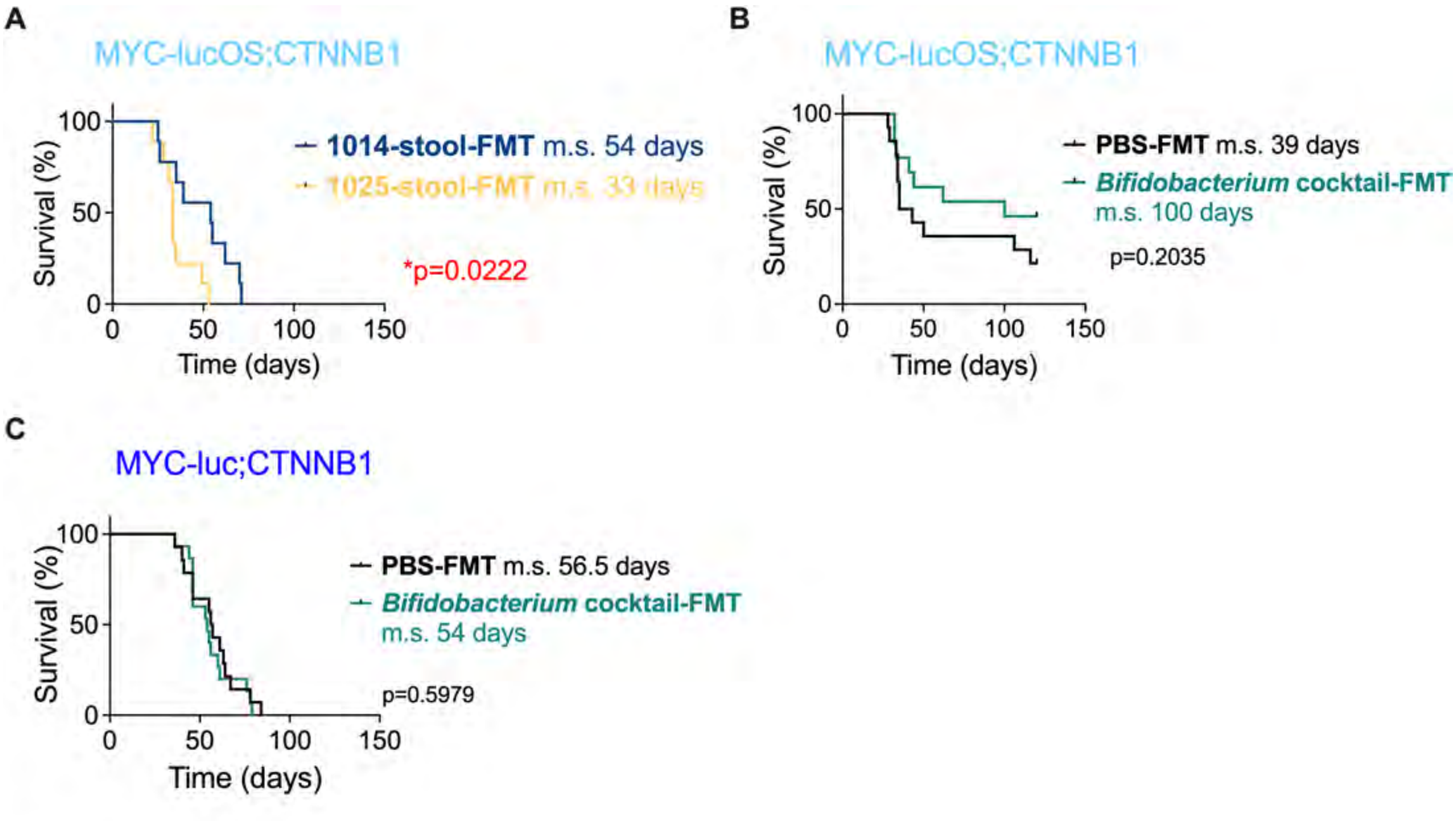


**S5**

**Supplementary figure S5.** A) Survival curve from SPF (ABX pre-treated) harboring *MYC-lucOS;CTNNB1* tumors upon FMT with 1025-stool or 1014-stool. Survival curves in SPF mice pre-treated with ABXs and colonized with 3-species of *Bifidobacterium* cocktail or PBS (Ctrl-group) by oral gavage harboring *MYC-lucOS;CTNNB1* (B) or *MYC-luc;CTNNB1* (C) tumors.
