# Supplementary material for: Bacteroidetes promote hepatocellular carcinoma progression and resistance to immunotherapy
Source: Hepatol Commun. 2026 Nov 11;10(12):e01086. doi: 10.1097/HC9.0000000000001086 (PMC13766593; doi:10.1097/HC9.0000000000001086)
Supplement: Supplementary file 2 [file hc9-10-e01086-s001.docx]

**SUPPLEMENTARY** **MATERIALS**

**Patient’s samples (extended information)**

**Cohort A**

‘Cohort A’ involved patients from R2810-ONC-1866, ph2a clinical trial (NCT03916627) with eligibility of enrolled patients previously described(1). Tumor response and progression assessments were performed according to modified Response Evaluation Criteria in Solid Tumors (RECIST) guideline version 1.1(2) without the requirement for confirmation of responses [Partial Response (PR)/ Complete Response (CR)] as this is not feasible within the neoadjuvant period. Stool samples for metagenomic profiling were collected at screening (V1), and at the 30-day follow-up visit on-treatment post-cemiplimab/pre-surgery (V2).

**Cohort B**

‘Cohort B’ involved patients from #CA027-005, ph2a multi-cohort and multi-arm trial of neoadjuvant nivolumab + BMS-813160 (CCR2/5-inhibitor) or BMS-986253 (anti-IL8) for NSCLC or HCC. Eligibility of patients with HCC included in this study were previously described(3). Response to neoadjuvant therapy was defined using RECIST v1.1(2), with Complete Response (CR) as the disappearance of all lesions in two observations at least 4 weeks apart, Partial Response (PR) as a ≥30% decrease in tumor burden compared to baseline in two observations at least 4 weeks apart, Progressive Disease (PD) as a ≥20% increase in tumor burden compared to nadir in two consecutive observations at least 4 weeks apart, and Stable Disease (SD) as neither a 30% decrease in tumor burden nor a 20% increase compared to nadir. Stool samples for metagenomic profiling were collected before initiation of nivolumab (V1) and within the week before surgery (V2) at the completion of neoadjuvant therapy.

**Cohort C**

‘Cohort C’ involved HCC patients recruited for the pilot study #STUDY-19-00777; IF2431642; IRB-19-02232 from clinics at Mount Sinai hospital (New York, US). Eligible individuals >18 years old, diagnosed with advanced-stage, unresectable, HCC, planned for treatment with an immunotherapy agent with or without additional targeted agents and had preserved hepatic function (Child-Pugh A or B). The cohort included patients that were treated with nivolumab, pembrolizumab, atezolizumab/bevacizumab, or lenvatinib/pembrolizumab. Exclusion criteria included patients that received lactulose/rifaximin or any other antibiotics in prior 30 days, history of organ transplant, autoimmune disease, or prior ICB therapy. Clinical outcome was assessed by best objective response based on at least 2 scans. Radiographic response (R and NR) was assigned per RECIST 1.1 criteria(2). Stool samples were collected at week 1, prior to ICB treatment (V1), and at 8-12 weeks, after treatment initiation (V2).

**Microbiomes 16s rRNA sequencing (in-house)**

Human samples were delivered to the MTC, where samples were aliquoted on liquid nitrogen and subsequently stored at −80°C until further processing. Stool samples from murine experiments were collected immediately upon defecation (t=0 samples) or upon sacrifice from the cecum (end-point samples), snap-frozen, and stored at −80°C until processed. Fecal sample aliquot sizes were optimized for DNA quantification, ensuring they fell within the linear range of the fecal DNA protocol (20–200 mg). Microbial DNA was extracted from stool samples using established methods involving bead beating with phenol:chloroform, as previously described(4,5). Purified DNA quantification was conducted using the Broad Range Quant-IT dsDNA Assay Kit in conjunction with a BioTek Synergy HTX Multi-Mode Reader. For 16S rRNA sequencing, custom barcoded primers were utilized to target and amplify the V4 variable region of the 16S rRNA gene, generating Illumina sequencing libraries as outlined in prior studies(6). The amplified products were purified using Beckman Coulter AMPure XP beads. Sequencing of prepared libraries was carried out on an Illumina MiSeq V2 platform following the manufacturer’s instructions. Raw 16S rRNA amplicon sequences were processed using the DADA2 software package, which included built-in algorithms for correcting amplicon sequencing errors(7). The SILVA 16S rRNA sequence database was employed for merging and aligning paired-end reads(8), resulting in an amplicon sequence variant (ASV) table.

**Microbiome analysis**

R statistical software (Rstudio Team)(9), phyloSeq(10), *vegan*(11), QIIME2(12) and DADA2(7) were used to perform downstream analysis and visualization of 16S rRNA sequencing data. With QIIME2 and DADA2 data processing, demultiplexing, and denoising using default parameters(7,12). For cohort analysis, we filtered out low abundance taxa, we removed ambiguously annotated phylum (e.g., “NA”) and excluded samples with fewer than 10,000 reads. We filtered out taxa present in less than 2% of samples. Microbial community composition beta diversity was analyzed using the Bray-Curtis distance metric. Ordination was performed using principal coordinates analysis (PCoA) to visualize differences in community structure. PERMANOVA was used to test for differences in beta-diversity between groups of interest while adjusting for cohort as a covariate. The analysis was conducted using the adonis function from the vegan R package. A Bray-Curtis distance matrix was computed from the microbiome data normalized to relative abundance. The PERMANOVA model assessed the variance in microbial community composition explained by the factor of interest (e.g., response to treatment), and when appropriate, accounting for the cohort effect. Significance was determined based on 999 permutations of the data. The proportion of variance explained and associated p-values were reported for both the factor-of-interest and cohort. The residual variance was also calculated to understand the proportion of unexplained variability. Taxonomic composition plots were generated based on average relative abundance of specific bacteria between groups (e.g.R vs NR). MaAsLin2 was used to identify significant taxa linear model using a linear model with the variable of interest (e.g. response) as a fixed effect, while adjusting for potential confounders. Features with p<0.05 were plotted. Alpha diversity was calculated using Shannon index on count data after removal of low-read samples and low abundance taxa, prior to prevalence filtering.

**Vectors for *in vivo* model**

The CMV-SB13 plasmid was kindly provided by Dr. Scott Lowe (MSKCC, New York). The *pT3-EF1a-N90-CTNNB1* (Addgene #31785) was kindly gifted from Dr. Xin Chen (University of California, San Francisco, CA). The *pT3-EF1a-MYC-IRES-luciferase-OS* (MYC-lucOS; Addgene plasmid #129776) and *pT3-EF1a-MYC-IRES-luciferase* (MYC-luc; Addgene plasmid #129775) were previously generated(13) from the *pT3-EF1a-MYC* vector (MYC, Addgene plasmid #92046) from Dr. Xin Chen (University of Hawaii Cancer Center) and the *Lenti-LucOS* (Addgene plasmid #22777) from Dr. Tyler Jacks (MIT Center for Cancer Research, Massachusetts, MA). All constructs were verified by nucleotide sequencing and vector integrity was confirmed by restriction enzyme digestion.

**Hydrodynamic tail-vein injection (HDTVI)**

Vectors were prepared at the following proportions considered for a 20g mouse: 11.4 μg of *pT3-EF1a-MYC-IRES-luciferase* (*MYC-luc*), 12 μg of *pT3-EF1a-MYC-IRES-luciferase-OS* (*MYC-lucOS*), 10 μg of *pT3-N90-CTNNB1 (CTNNB1*) and a 4:1 ratio of transposon to *SB13* transposase–encoding plasmid in 2mL of sterile 0.9% NaCl solution (Intermountain). Injections of 10% of the weight of each mouse in volume were adjusted to the precise weight of mice at injection. Hepatocytes transfection of the plasmids was achieved by performing lateral tail vein injection within 5-7 seconds, creating a temporary congestion, which then flows back into the hepatic veins. Only the hepatocytes transfected with all three plasmids (transposon-based, CRISPR-based or transposon-based, and transposase-encoding) will have the potential to form tumors, since two independent “hits” are necessary for malignant transformation in C57BL/6 mice(14). Vectors for hydrodynamic delivery were produced by Genewiz (Azenta Life Sciences Genomics, NJ). Equivalent DNA concentration between different batches of DNA was confirmed to ensure reproducibility among experiments.

***In vivo* treatments**

ABXs were administrated *ad libitum*, protected from light and refreshing the treatment every 3-4 days. ABXs were diluted in sterile autoclaved water at 1g/l for Ampicillin (Sigma-Aldrich) and Metronidazole (Sigma-Aldrich), and at 0.5g/l for Neomycin (Sigma-Aldrich) and Vancomycin (Chem-Impex). Mice were subjected to ABXs 24h post-HDTVI and sustained until sacrifice/dead of mice. InVivoMAb anti-mouse PD-L1 (B7-H1) clone 10F.9G2™ isotype: Rat IgG2b (BioXcell) was administrated in three doses of 200μg/mouse intraperitoneally at days 14, 16, and 18 post-HDTVI.

**Hematoxylin and eosin (H&E) stain**

H&E staining of murine liver was performed using 4 μm-thick sections from formalin-fixed and paraffin-embedded tissues (FFPE). Tissue deparaffinization and re-hydration was performed through three 5-minute serial xylene treatments followed by incubation in decreasing ethanol concentrations (100%, 100%, 90%, and 80%) for 3 minutes each, then incubated in distilled water for 5 minutes. Stain was performed as followed: 5 minutes in Haematoxylin (Sigma-Aldrich) followed by differentiation with 0.3% acid alcohol for 2 seconds and rinse in Scott’s tap water substitute for 5 minutes and final stain in eosin for 2 minutes. Rinse in running tap water for 5 minutes was performed in between all the steps. Last wash step in running water was for 30 minutes. Then tissue was dehydrated in serial ethanol (90% 15 seconds, 100% 15 seconds, 100% 15 seconds) and xylene (twice for 5 minutes). Slides were mounted with Permount Media, dried at RT and scanned at 20x resolution using Hamamatsu, NanoZoomer S60.

**RNA extraction, sequencing, and analysis**

Total RNA was isolated from 15-20mg mouse liver tumors using Trizol® (Invitrogen) tissue homogenizer followed by digestion with Dnase I (Roche) and purification with the Rneasy Kit (Qiagen). RNA sequencing (poly-A selected, PE150, 40 million reads) was performed through Genewiz (Azenta Life Sciences Genomics, NJ). Read counts for each transcript were measured using featureCounts and differentially expressed genes were determined using DESeq2 (cut-off of 0.05 on adjusted p-value corrected for multiple hypotheses testing). Gene Set Enrichment Analysis (GSEA) was then conducted on genes ranked according to differential expression statistics. Enrichment was assessed using six MSigDB gene-set collections: Hallmark (H), WikiPathways (C2:CP), Reactome (C2:CP), Immunologic Signatures (C7), KEGG Legacy (C2), and Gene Ontology Biological Processes (C5:GO). Results are publicly available at Zenodo under number [GSEA694647](https://doi.org/10.5281/zenodo.20660952).

**Isolation of immune cells and flow cytometry**

Isolation of liver immune cells and staining for flow cytometry was performed as previously (9,10). The liver was perfused with 2 mM PBS-EDTA, chopped, and enzymatically digested with HBSS (Corning) supplemented with 1 mg/ml collagenase IV (SIGMA) and DNase I 0.02 mg/ml (SIGMA) for 30 minutes at 37ºC in agitation. The digestion was neutralized with HBSS and the liver suspension was strained through a 70 μm nylon mesh (Corning) and centrifuged at 30g at room temperature for 3 minutes. The supernatant was then centrifuged at 600g at 4ºC for 5 minutes. After centrifugation, the pellet was re-suspended in 36% HBSS-percoll and centrifuged at 800g at 4ºC for 20 minutes without breaks. The resultant pellet was incubated with ACK lysing buffer (Gibco). One million cells were resuspended in blocking solution (2% BSA in PBS), and Fc receptors were blocked using BioLegend TruStain FcX PLUS anti-mouse CD16/32 antibody according to the manufacturer’s instructions. Cells were then washed and stained with fluorochrome-conjugated antibodies (BioLegend or eBioscience) targeting TCRγδ (BV421), CD45 (BV510), CD8 (BV650), NK1.1 (BV711), CD4 (FITC), CD25 (PE-Dazzle 594), FoxP3 PE-Cy7) and CD3 (APC-Cy7) for lymphoid analysis, and CD11c (BV421), CD45 (BV510), CD103 (BV605), CD11b (BV650), Ly6C (BV711), NK1.1 (FITC), B220 (FITC), TCRb (FITC), F4/80 (PE), Ly6G (PE-Cy7), CD24 (APC), and MHC-II (APC-Cy7) for myeloid analysis. Lineage exclusion in the myeloid panel was performed using a FITC dump channel containing NK1.1, B220, and TCRβ. Fixation and permeabilization were performed using the eBioscience Foxp3/Transcription Factor Staining Buffer Set (Thermo Fisher) according to the manufacturer’s instructions. Samples were acquired on an LSRFortessa flow cytometer (BD Biosciences) at the Tisch Cancer Institute Flow Cytometry Core. Compensation was performed using single-stained compensation beads (Thermo Fisher) and applied prior to analysis. Positive gates were defined using fluorescence-minus-one (FMO) controls for each marker.

Data were analyzed using FlowJo software. Doublets were excluded using height versus area dot plots and samples were additionally gated on viable leukocytes by DAPI exclusion. For lymphocyte analysis, live CD45⁺ cells were gated on CD3 expression to identify T cells, which were further subdivided into CD4⁺, CD8⁺, TCRγδ⁺, and FoxP3^+^CD25⁺ (Treg-enriched) subsets. NK cells were defined as CD3⁻NK1.1⁺ cells, while NKT-like cells were identified as CD3⁺NK1.1⁺. For myeloid analysis, CD45⁺ live singlets were first separated into Ly6G⁺ neutrophils and Ly6G⁻ populations. Within the Ly6G⁻ compartment, lineage-positive cells (NK1.1⁺, B220⁺, TCRβ⁺) were excluded. To avoid overlap between myeloid lineages, dendritic cells (DCs) were first isolated from the remaining population as CD11c⁺MHC-II⁺ cells and further subdivided into CD103⁺CD11b⁻ conventional type 1 dendritic cells (cDC1s) and CD11b⁺CD103⁻ conventional type 2 dendritic cells (cDC2s), with CD24 expression used to refine these subsets. From the non-DC compartment, Kupffer cells (KCs) were defined as F4/80^hiCD11b^lo cells. Monocytes were identified as Ly6C⁺F4/80⁻/lo cells, monocyte/macrophage populations (Mono/Macs) as CD11b⁺F4/80⁺ cells, and tumor-associated macrophages (TAMs) were refined as CD11b⁺F4/80⁺Ly6C⁻/lo cells.

**REFERENCES**

1.	Marron TU, Fiel MI, Hamon P, Fiaschi N, Kim E, Ward SC, et al. Neoadjuvant cemiplimab for resectable hepatocellular carcinoma: a single-arm, open-label, phase 2 trial. Lancet Gastroenterol Hepatol. 2022 Mar;7(3):219–29. doi:10.1016/S2468-1253(21)00385-X PubMed PMID: 35065058; PubMed Central PMCID: PMC9901534.

2.	Eisenhauer EA, Therasse P, Bogaerts J, Schwartz LH, Sargent D, Ford R, et al. New response evaluation criteria in solid tumours: revised RECIST guideline (version 1.1). Eur J Cancer Oxf Engl 1990. 2009 Jan;45(2):228–47. doi:10.1016/j.ejca.2008.10.026 PubMed PMID: 19097774.

3.	Venturini N, Marron T, Casanova-Acebes M, Mandeli J, Doroshow D, Lucas N, et al. 629 Neoadjuvant nivolumab combined with CCR2/5 inhibitor or anti-IL-8 antibody in non-small cell lung cancer and hepatocellular carcinoma. In: Journal for ImmunoTherapy of Cancer. 2022. p. A660–A660. doi:10.1136/jitc-2022-SITC2022.0629

4.	Britton GJ, Contijoch EJ, Mogno I, Vennaro OH, Llewellyn SR, Ng R, et al. Microbiotas from Humans with Inflammatory Bowel Disease Alter the Balance of Gut Th17 and RORγt(+) Regulatory T Cells and Exacerbate Colitis in Mice. Immunity. 2019 Jan 15;50(1):212-224.e4. doi:10.1016/j.immuni.2018.12.015 PubMed PMID: 30650377; PubMed Central PMCID: PMC6512335.

5.	Contijoch EJ, Britton GJ, Yang C, Mogno I, Li Z, Ng R, et al. Gut microbiota density influences host physiology and is shaped by host and microbial factors. eLife. 2019 Jan 22;8. doi:10.7554/eLife.40553 PubMed PMID: 30666957; PubMed Central PMCID: PMC6342524.

6.	Faith JJ, Guruge JL, Charbonneau M, Subramanian S, Seedorf H, Goodman AL, et al. The long-term stability of the human gut microbiota. Science. 2013 Jul 5;341(6141):1237439. doi:10.1126/science.1237439 PubMed PMID: 23828941; PubMed Central PMCID: PMC3791589.

7.	Callahan BJ, McMurdie PJ, Rosen MJ, Han AW, Johnson AJA, Holmes SP. DADA2: High-resolution sample inference from Illumina amplicon data. Nat Methods. 2016 Jul;13(7):581–3. doi:10.1038/nmeth.3869 PubMed PMID: 27214047; PubMed Central PMCID: PMC4927377.

8.	Quast C, Pruesse E, Yilmaz P, Gerken J, Schweer T, Yarza P, et al. The SILVA ribosomal RNA gene database project: improved data processing and web-based tools. Nucleic Acids Res. 2013 Jan;41(Database issue):D590-596. doi:10.1093/nar/gks1219 PubMed PMID: 23193283; PubMed Central PMCID: PMC3531112.

9.	RStudio Team. RStudio: Integrated Development Environment for R [Internet]. RStudio, PBC Lugar: Boston, MA; 2020. Available from: http://www.rstudio.com/

10.McMurdie PJ, Holmes S. phyloseq: An R Package for Reproducible Interactive Analysis and Graphics of Microbiome Census Data. PLOS ONE. 2013 Apr;8(4):1–11. doi:10.1371/journal.pone.0061217

11.Oksanen J, Blanchet FG, Kindt R, Legendre P, Minchin P, O’Hara B, et al. Vegan: Community Ecology Package. R Package Version 22-1. 2015 Jan;2:1–2.

12.Estaki M, Jiang L, Bokulich NA, McDonald D, González A, Kosciolek T, et al. QIIME 2 Enables Comprehensive End-to-End Analysis of Diverse Microbiome Data and Comparative Studies with Publicly Available Data. Curr Protoc Bioinforma. 2020 Jun;70(1):e100. doi:10.1002/cpbi.100 PubMed PMID: 32343490; PubMed Central PMCID: PMC9285460.

13.Ruiz de Galarreta M, Bresnahan E, Molina-Sánchez P, Lindblad KE, Maier B, Sia D, et al. β-Catenin Activation Promotes Immune Escape and Resistance to Anti-PD-1 Therapy in Hepatocellular Carcinoma. Cancer Discov. 2019 Aug;9(8):1124–41. doi:10.1158/2159-8290.CD-19-0074 PubMed PMID: 31186238; PubMed Central PMCID: PMC6677618.

14.Molina-Sánchez P, Ruiz de Galarreta M, Yao MA, Lindblad KE, Bresnahan E, Bitterman E, et al. Cooperation Between Distinct Cancer Driver Genes Underlies Intertumor Heterogeneity in Hepatocellular Carcinoma. Gastroenterology. 2020 Dec;159(6):2203-2220.e14. doi:10.1053/j.gastro.2020.08.015 PubMed PMID: 32814112; PubMed Central PMCID: PMC7726023.

**SUPPLEMENTRY FIGURE LEGENDS**

**Supplementary figure S1.** α-diversity Shannon plot comparing R and NR baseline (V1) stools from A) cohort A and B) cohort C. Relative abundance (%) phylum plots of C) *Firmicutes*, D) *Actinobacteria,* E) *Fusobacteria*, and F) *Proteobacteria* of R vs NR baseline microbiomes (V1) from cohort A. Relative abundance (%) phylum plots of G) *Firmicutes*, H*) Actinobacteria,* I) *Fusobacteria*, and J) *Proteobacteria* of R vs NR baseline microbiomes (V1) from cohort C. K) Significantly enriched species in NR vs R (MaAsLin2 , p<0.05) from V1 samples across cohorts A-C. R= responders (turquoise) and NR= non-responders (pink).

**Supplementary figure S2.** A) Representative pictures of H&E stains of 5 murine livers at 3 weeks post HDTVI of *MYC-lucOS;CTNNB1* of 5 different groups from left to right: untreated (Ctrl), ampicillin-treated, vancomycin-treated, neomycin-treated, and metronidazole-treated. B) Survival curve from mice harboring *MYC-lucOS;CTNNB1* tumors treated with neomycin and compared with untreated (Ctrl). Dot-style line indicates that these results were already included in a previous graph within this manuscript, as the experiments were run together, a unique control group was used to reduce the number of mice to utilize. C) Volcano plot of bulk RNA sequencing of murine *MYC-lucOS;CTNNB1* tumors treated with vancomycin or without treatment (Ctrl) in survival experiment. Quantification of the number of Neutrophils (D) and lymphoid subpopulations (E) including Total T cells, CD4+ T cells, CD8+ T cells, γδT cells, Treg cells, NK cells and NKT cells in the livers of the corresponding mice (n = 4–5 per group).

**Supplementary figure S3.** A) α-diversity Shannon plot comparing the microbiomes from murine stools collected from t0 (grey), ctrl (black), ampicillin (yellow), vancomycin (green), neomycin (magenta) and metronidazole (blue) groups. B) Bray-Curtis dissimilarity PcoA plot of β-diversity comparing the microbiomes from murine stools collected from t0 (grey), ctrl (black), ampicillin (yellow), vancomycin (green), neomycin (magenta) and metronidazole (blue) groups. C) Representative plots of average phylum composition from cecum stools collected from 5 Ctrl (water) mice, 5 vancomycin-treated mice, 5 ampicillin-treated mice, 5 neomycin-treated mice and 5 metronidazole-treated mice for 3 weeks upon *MYC-lucOS;CTNNB1* HDTVI. D) Heatmap plot of average % relative abundance of *Bacteroidetes* species in stools from ctrl-, ampicillin-, neomycin- and metronidazole-treated mice.

**Supplementary figure S4.** A) Schematic of experiment design of survival experiment on C57BL6 WT female GF mice harboring *MYC-lucOS;CTNNB1* tumors after FMT with blended stools previously generated from vancomycin-treated mice stools (‘Vanco-stool’), and control-treated mice stools (‘Ctrl-stool’) generated by BioRender. B) Survival curve of ex-GF mice harboring *MYC-lucOS;CTNNB1* tumors upon FMT with ‘Vanco-stool’ or ‘Ctrl-stool’. Parts of whole tables on the right, showing tumor penetrance at endpoint (200 days post-HDTVI).

**Supplementary figure S5.** A) Survival curve from SPF (ABX pre-treated) harboring *MYC-lucOS;CTNNB1* tumors upon FMT with 1025-stool or 1014-stool. Survival curves in SPF mice pre-treated with ABXs and colonized with 3-species of *Bifidobacterium* cocktail or PBS (Ctrl-group) by oral gavage harboring *MYC-lucOS;CTNNB1* (B) or *MYC-luc;CTNNB1* (C) tumors.
