# Supplementary material for: Bacteroidetes promote hepatocellular carcinoma progression and resistance to immunotherapy
Source: Hepatol Commun. 2026 Nov 11;10(12):e01086. doi: 10.1097/HC9.0000000000001086 (PMC13766593; doi:10.1097/HC9.0000000000001086)
Supplement: Supplementary file 3 [file hc9-10-e01086-s002.docx]

**Supp. Table 1.** Gene-Set-Enrichment-Analysis (GSEA) results from bulkRNAseq

| **Enriched Gene-set-hallmarks in Ctrl** | SIZE | | ES | NES | NOM p-val FDR q-val | | FWER p-val | RANK AT MAX LEADING EDGE |
| --- | --- | --- | --- | --- | --- | --- | --- | --- |
| REACTOME_MATURATION_OF_DENV_PROTEINS |  | 51 | -0.6812999 | -1.9528292 | 0 | 0.0381 | 0.151 | 2566 tags=45%, list=13%, signal=52% |
| WP_ANGIOGENESIS_OVERVIEW |  | 63 | -0.6393056 | -1.9027101 | 0 | 0.0386 | 0.374 | 1503 tags=25%, list=8%, signal=27% |
| WP_ZINC_HOMEOSTASIS |  | 27 | -0.7557689 | -1.9448526 | 0 | 0.0389 | 0.18 | 1922 tags=37%, list=10%, signal=41% |
| GOBP_MODIFIED_AMINO_ACID_TRANSPORT |  | 39 | -0.7041199 | -1.9056935 | 0 | 0.0398 | 0.362 | 2129 tags=38%, list=11%, signal=43% |
| GOBP_TRANSITION_METAL_ION_TRANSPORT |  | 90 | -0.643799 | -1.9864506 | 0 | 0.04 | 0.071 | 3792 tags=46%, list=19%, signal=56% |
| REACTOME_UNFOLDED_PROTEIN_RESPONSE_UPR |  | 87 | -0.6327953 | -1.9728093 | 0 | 0.0401 | 0.1 | 4486 tags=47%, list=23%, signal=61% |
| REACTOME_RESPONSE_TO_ELEVATED_PLATELET_CYTOSOLIC_CA2 |  | 122 | -0.5897817 | -1.9337403 | 0 | 0.0416 | 0.224 | 2519 tags=29%, list=13%, signal=33% |
| GSE22935_UNSTIM_VS_24H_MBOVIS_BCG_STIM_MYD88_KO_MACROPHAGE_UP |  | 187 | -0.5606985 | -1.9109644 | 0 | 0.0416 | 0.326 | 2206 tags=28%, list=11%, signal=31% |
| KEGG_VIBRIO_CHOLERAE_INFECTION |  | 51 | -0.6720491 | -1.9194572 | 0 | 0.0418 | 0.277 | 2098 tags=39%, list=11%, signal=44% |
| WP_SPHINGOLIPID_PATHWAY |  | 25 | -0.7637159 | -1.9066626 | 0 | 0.042 | 0.351 | 2323 tags=56%, list=12%, signal=63% |
| WP_KISSPEPTINKISSPEPTIN_RECEPTOR_SYSTEM_IN_THE_OVARY |  | 37 | -0.7086375 | -1.9120129 | 0 | 0.0448 | 0.32 | 2545 tags=38%, list=13%, signal=43% |
| GOBP_INTRACELLULAR_ZINC_ION_HOMEOSTASIS |  | 26 | -0.7579626 | -1.9214782 | 0 | 0.0449 | 0.268 | 681 tags=27%, list=3%, signal=28% |
| GSE40273_EOS_KO_VS_WT_TREG_UP |  | 179 | -0.5500448 | -1.8544519 | 0 | 0.0581 | 0.697 | 3169 tags=40%, list=16%, signal=47% |
| GOBP_ENDOPLASMIC_RETICULUM_TO_CYTOSOL_TRANSPORT |  | 24 | -0.7517899 | -1.8768995 | 0 | 0.0582 | 0.53 | 2109 tags=50%, list=11%, signal=56% |
| GSE339_CD4POS_VS_CD8POS_DC_DN |  | 182 | -0.5337892 | -1.8382864 | 0 | 0.0584 | 0.794 | 2712 tags=29%, list=14%, signal=33% |
| KEGG_DRUG_METABOLISM_CYTOCHROME_P450 |  | 39 | -0.6746992 | -1.8396045 | 0 | 0.0592 | 0.786 | 2938 tags=38%, list=15%, signal=45% |
| GSE6674_CPG_VS_PL2_3_STIM_BCELL_DN |  | 169 | -0.539361 | -1.8477939 | 0 | 0.0595 | 0.735 | 4631 tags=44%, list=24%, signal=58% |
| GOBP_GLYCOPROTEIN_METABOLIC_PROCESS |  | 343 | -0.5145598 | -1.8555439 | 0 | 0.0597 | 0.691 | 3200 tags=29%, list=16%, signal=35% |
| GSE6674_ANTI_IGM_VS_PL2_3_STIM_BCELL_DN |  | 174 | -0.5418662 | -1.8429826 | 0 | 0.06 | 0.763 | 4215 tags=34%, list=21%, signal=43% |
| GOBP_ESTABLISHMENT_OF_PROTEIN_LOCALIZATION_TO_ENDOPLASMIC_RETICULUM |  | 38 | -0.6966709 | -1.8682606 | 0 | 0.0603 | 0.595 | 3319 tags=58%, list=17%, signal=70% |
| GSE6674_UNSTIM_VS_ANTI_IGM_AND_CPG_STIM_BCELL_DN |  | 171 | -0.5471528 | -1.8570606 | 0 | 0.0604 | 0.672 | 4215 tags=38%, list=21%, signal=48% |
| WP_MIRTARGETED_GENES_IN_SQUAMOUS_CELL |  | 127 | -0.5658755 | -1.8439499 | 0 | 0.0608 | 0.757 | 1179 tags=18%, list=6%, signal=19% |
| GOBP_GLYCOPROTEIN_BIOSYNTHETIC_PROCESS |  | 282 | -0.5262614 | -1.8617376 | 0 | 0.0618 | 0.642 | 2604 tags=27%, list=13%, signal=30% |
| **GSE27859_MACROPHAGE_VS_DC_UP** |  | 156 | -0.5640405 | -1.8703651 | 0 | 0.0624 | 0.581 | 3612 tags=35%, list=18%, signal=43% |
| REACTOME_DENGUE_VIRUS_GENOME_TRANSLATION_AND_REPLICATION |  | 80 | -0.5963855 | -1.827986 | 0 | 0.0642 | 0.85 | 2566 tags=35%, list=13%, signal=40% |
| REACTOME_SYNDECAN_INTERACTIONS |  | 27 | -0.7072161 | -1.810467 | 0 | 0.0664 | 0.93 | 3841 tags=52%, list=20%, signal=64% |
| KEGG_ALPHA_LINOLENIC_ACID_METABOLISM |  | 15 | -0.803822 | -1.8129491 | 0 | 0.0665 | 0.924 | 905 tags=27%, list=5%, signal=28% |
| WP_MIRTARGETED_GENES_IN_MUSCLE_CELL |  | 333 | -0.500927 | -1.8141679 | 0 | 0.0671 | 0.919 | 3472 tags=28%, list=18%, signal=33% |
| REACTOME_GLUTATHIONE_CONJUGATION |  | 33 | -0.6881092 | -1.8220409 | 0 | 0.0673 | 0.886 | 580 tags=24%, list=3%, signal=25% |
| WP_TRANSCRIPTIONAL_ACTIVATION_BY_NRF2_IN_RESPONSE_TO_PHYTOCHEMICALS |  | 15 | -0.8137228 | -1.8106577 | 0 | 0.0678 | 0.929 | 1751 tags=47%, list=9%, signal=51% |
| **GSE25088_ROSIGLITAZONE_VS_IL4_AND_ROSIGLITAZONE_STIM_STAT6_KO_MACROPHAGE_DAY10_DN** |  | 163 | -0.5390761 | -1.8192729 | 0 | 0.0684 | 0.899 | 4056 tags=36%, list=21%, signal=45% |
| **GSE7348_LPS_VS_TOLERIZED_AND_LPS_STIM_MACROPHAGE_UP** |  | 171 | -0.5381703 | -1.8144668 | 0 | 0.0686 | 0.917 | 3421 tags=30%, list=17%, signal=36% |
| GOBP_ZINC_ION_TRANSPORT |  | 34 | -0.6827472 | -1.8148184 | 0 | 0.0719 | 0.916 | 3171 tags=38%, list=16%, signal=46% |
| REACTOME_BIOSYNTHESIS_OF_THE_N_GLYCAN_PRECURSOR_DOLICHOL_LIPID_LINKED_OLIGOSACCHARIDE_LLO_AND_TRANSFER_TO_A_NAS | | 76 | -0.5867921 | -1.7988131 | 0 | 0.0751 | 0.96 | 2962 tags=30%, list=15%, signal=36% |
| REACTOME_IRE1ALPHA_ACTIVATES_CHAPERONES | | 47 | -0.7009933 | -1.9887613 | 0 | 0.0756 | 0.067 | 3595 tags=53%, list=18%, signal=65% |
| WP_MIRTARGETED_GENES_IN_LEUKOCYTES | | 128 | -0.5507439 | -1.7992207 | 0 | 0.0763 | 0.96 | 2627 tags=24%, list=13%, signal=28% |
| GSE6269_E_COLI_VS_STAPH_AUREUS_INF_PBMC_DN | | 152 | -0.5458111 | -1.793121 | 0 | 0.0768 | 0.966 | 3386 tags=37%, list=17%, signal=44% |
| GSE360_L_MAJOR_VS_B_MALAYI_HIGH_DOSE_DC_UP | | 175 | -0.5232014 | -1.7936409 | 0 | 0.0777 | 0.966 | 2741 tags=30%, list=14%, signal=34% |
| REACTOME_ASPARAGINE_N_LINKED_GLYCOSYLATION | | 291 | -0.5023215 | -1.7873778 | 0 | 0.0827 | 0.969 | 2962 tags=32%, list=15%, signal=37% |
| GSE18281_CORTICAL_VS_MEDULLARY_THYMOCYTE_UP | | 167 | -0.5277717 | -1.7689441 | 0 | 0.0865 | 0.993 | 2712 tags=30%, list=14%, signal=34% |
| WP_NRF2_PATHWAY | | 119 | -0.5435557 | -1.7693319 | 0 | 0.0875 | 0.993 | 1964 tags=22%, list=10%, signal=24% |
| GSE36009_WT_VS_NLRP10_KO_DC_DN | | 185 | -0.5178906 | -1.7667211 | 0 | 0.0879 | 0.994 | 3316 tags=36%, list=17%, signal=43% |
| REACTOME_SYNTHESIS_OF_SUBSTRATES_IN_N_GLYCAN_BIOSYTHESIS | | 62 | -0.5916341 | -1.7696532 | 0 | 0.0886 | 0.993 | 2895 tags=29%, list=15%, signal=34% |
| GSE1791_CTRL_VS_NEUROMEDINU_IN_T_CELL_LINE_6H_UP | | 176 | -0.5116199 | -1.7494369 | 0 | 0.089 | 0.999 | 2957 tags=30%, list=15%, signal=35% |
| GSE14769_UNSTIM_VS_60MIN_LPS_BMDM_DN | | 181 | -0.5159627 | -1.74837 | 0 | 0.0892 | 0.999 | 3498 tags=34%, list=18%, signal=41% |
| WP_WNT_SIGNALING_NETPATH | | 51 | -0.6133732 | -1.747581 | 0 | 0.0892 | 0.999 | 3938 tags=47%, list=20%, signal=59% |
| REACTOME_SRP_DEPENDENT_COTRANSLATIONAL_PROTEIN_TARGETING_TO_MEMBRANE | | 106 | -0.5563719 | -1.7745342 | 0 | 0.09 | 0.986 | 3319 tags=19%, list=17%, signal=23% |
| **GSE2770_IL4_ACT_VS_ACT_CD4_TCELL_6H_DN** | | 159 | -0.5272626 | -1.7451217 | 0 | 0.0903 | 0.999 | 3326 tags=30%, list=17%, signal=35% |
| REACTOME_N_GLYCAN_TRIMMING_IN_THE_ER_AND_CALNEXIN_CALRETICULIN_CYCLE | | 34 | -0.6684136 | -1.7717087 | 0 | 0.0906 | 0.991 | 2359 tags=53%, list=12%, signal=60% |
| REACTOME_MATURATION_OF_SARS_COV_2_SPIKE_PROTEIN | | 40 | -0.6563884 | -1.7728046 | 0 | 0.0907 | 0.988 | 2933 tags=60%, list=15%, signal=70% |
| **GSE30971_CTRL_VS_LPS_STIM_MACROPHAGE_WBP7_KO_2H_UP** | | 160 | -0.519375 | -1.7453439 | 0 | 0.091 | 0.999 | 2469 tags=30%, list=13%, signal=34% |
| WP_COMMON_PATHWAYS_UNDERLYING_DRUG_ADDICTION | | 37 | -0.6460869 | -1.751304 | 0 | 0.0912 | 0.999 | 3470 tags=41%, list=18%, signal=49% |
| GSE24142_EARLY_THYMIC_PROGENITOR_VS_DN2_THYMOCYTE_FETAL_UP | | 180 | -0.5099366 | -1.751376 | 0 | 0.0924 | 0.999 | 2482 tags=26%, list=13%, signal=30% |
| **GSE25088_IL4_VS_IL4_AND_ROSIGLITAZONE_STIM_STAT6_KO_MACROPHAGE_DAY10_DN** | | 162 | -0.517095 | -1.7555188 | 0 | 0.0945 | 0.995 | 3252 tags=35%, list=17%, signal=41% |
| KEGG_VEGF_SIGNALING_PATHWAY | | 71 | -0.5729427 | -1.7535298 | 0 | 0.0948 | 0.997 | 1604 tags=21%, list=8%, signal=23% |
| **GSE12392_IFNAR_KO_VS_IFNB_KO_CD8_NEG_SPLEEN_DC_UP** | | 167 | -0.5144401 | -1.7542803 | 0 | 0.0949 | 0.996 | 3190 tags=29%, list=16%, signal=35% |
| GOBP_PROTEIN_O_LINKED_GLYCOSYLATION | | 132 | -0.5360776 | -1.7557836 | 0 | 0.0955 | 0.995 | 2212 tags=25%, list=11%, signal=28% |
| WP_SPHINGOLIPID_METABOLISM_OVERVIEW | | 22 | -0.7235873 | -1.7589761 | 0 | 0.0963 | 0.995 | 2323 tags=50%, list=12%, signal=57% |
| **GSE32128_INOS_DEPENDENT_VS_INOS_INDEPENDENT_ACTIVATED_TCELL_UP** | | 175 | -0.5084322 | -1.7562293 | 0 | 0.0965 | 0.995 | 3081 tags=33%, list=16%, signal=39% |
| REACTOME_REGULATION_OF_CDH1_POSTTRANSLATIONAL_PROCESSING_AND_TRAFFICKING_TO_PLASMA_MEMBRANE | | 31 | -0.6716467 | -1.757256 | 0 | 0.0968 | 0.995 | 3474 tags=71%, list=18%, signal=86% |
| **GSE17974_1H_VS_72H_UNTREATED_IN_VITRO_CD4_TCELL_UP** | | 169 | -0.5082994 | -1.7389858 | 0 | 0.0976 | 1 | 3170 tags=30%, list=16%, signal=35% |
| GSE6269_HEALTHY_VS_STAPH_PNEUMO_INF_PBMC_DN | | 142 | -0.5241023 | -1.735411 | 0 | 0.099 | 1 | 4183 tags=44%, list=21%, signal=56% |
| GSE45365_NK_CELL_VS_BCELL_UP | | 183 | -0.509986 | -1.7335855 | 0 | 0.1002 | 1 | 3066 tags=31%, list=16%, signal=36% |
| **GSE30971_CTRL_VS_LPS_STIM_MACROPHAGE_WBP7_KO_4H_UP** | | 169 | -0.5102314 | -1.729503 | 0 | 0.1018 | 1 | 2220 tags=27%, list=11%, signal=30% |

| WP_NGLYCAN_BIOSYNTHESIS | 52 | -0.6054378 | -1.7296295 |  | 0.1027 | 1 | 2962 tags=48%, list=15%, signal=56% |
| --- | --- | --- | --- | --- | --- | --- | --- |
| GSE43260_BTLA_POS_VS_NEG_INTRATUMORAL_CD8_TCELL_UP | 183 | -0.5122891 | -1.726943 | 0 | 0.1031 | 1 | 3139 tags=31%, list=16%, signal=37% |
| GSE21546_ELK1_KO_VS_SAP1A_KO_AND_ELK1_KO_ANTI_CD3_STIM_DP_THYMOCYTES_DN | 176 | -0.5125077 | -1.7273856 | 0 | 0.1036 | 1 | 3761 tags=32%, list=19%, signal=39% |
| GSE14769_40MIN_VS_360MIN_LPS_BMDM_DN | 183 | -0.4994118 | -1.7167872 | 0 | 0.1089 | 1 | 3303 tags=32%, list=17%, signal=38% |
| GSE14769_20MIN_VS_360MIN_LPS_BMDM_DN | 177 | -0.501114 | -1.7183506 | 0 | 0.109 | 1 | 3210 tags=32%, list=16%, signal=38% |
| GSE23925_DARK_ZONE_VS_NAIVE_BCELL_DN | 180 | -0.5005562 | -1.7093414 | 0 | 0.1133 | 1 | 3383 tags=32%, list=17%, signal=39% |
| REACTOME_TRANSCRIPTIONAL_REGULATION_BY_THE_AP_2_TFAP2_FAMILY_OF_TRANSCRIPTION_FACTORS | 34 | -0.6440986 | -1.7113676 | 0 | 0.1137 | 1 | 1654 tags=21%, list=8%, signal=22% |
| GOBP_CAMERA_TYPE_EYE_PHOTORECEPTOR_CELL_DIFFERENTIATION | 27 | -0.6690512 | -1.7094635 | 0 | 0.1142 | 1 | 1914 tags=22%, list=10%, signal=25% |
| GSE37416_CTRL_VS_48H_F_TULARENSIS_LVS_NEUTROPHIL_DN | 170 | -0.5052515 | -1.7036926 | 0 | 0.1176 | 1 | 4906 tags=42%, list=25%, signal=56% |
| **GSE20754_WT_VS_TCF1_KO_MEMORY_CD8_TCELL_UP** | 179 | -0.49951 | -1.7025218 | 0 | 0.1182 | 1 | 3446 tags=32%, list=18%, signal=38% |
| **GSE22103_LPS_VS_GMCSF_AND_IFNG_STIM_NEUTROPHIL_DN** | 177 | -0.5025281 | -1.7039183 | 0 | 0.1184 | 1 | 3303 tags=30%, list=17%, signal=36% |
| GSE17721_LPS_VS_PAM3CSK4_1H_BMDC_DN | 184 | -0.4938799 | -1.6998134 | 0 | 0.1202 | 1 | 2972 tags=28%, list=15%, signal=32% |
| GOBP_RESPONSE_TO_ENDOPLASMIC_RETICULUM_STRESS | 258 | -0.4798223 | -1.6888354 | 0 | 0.1263 | 1 | 3474 tags=34%, list=18%, signal=41% |
| GSE12366_PLASMA_CELL_VS_MEMORY_BCELL_UP | 156 | -0.5041289 | -1.6932741 | 0 | 0.1263 | 1 | 2532 tags=26%, list=13%, signal=29% |
| GSE2197_IMMUNOSUPPRESSIVE_DNA_VS_UNTREATED_IN_DC_UP | 180 | -0.4976116 | -1.6892763 | 0 | 0.1266 | 1 | 3239 tags=31%, list=17%, signal=37% |
| **GSE30971_WBP7_HET_VS_KO_MACROPHAGE_4H_LPS_STIM_UP** | 169 | -0.5025606 | -1.689738 | 0 | 0.1268 | 1 | 1954 tags=20%, list=10%, signal=21% |
| GSE37416_0H_VS_48H_F_TULARENSIS_LVS_NEUTROPHIL_DN | 170 | -0.4962837 | -1.6917503 | 0 | 0.1277 | 1 | 4867 tags=46%, list=25%, signal=60% |
| **GSE36888_UNTREATED_VS_IL2_TREATED_STAT5_AB_KNOCKIN_TCELL_6H_UP** | 154 | -0.5043466 | -1.6910834 | 0 | 0.1277 | 1 | 2550 tags=25%, list=13%, signal=29% |
| GSE17301_ACD3_ACD28_VS_ACD3_ACD28_AND_IFNA2_STIM_CD8_TCELL_DN | 173 | -0.4969227 | -1.6860151 | 0 | 0.1288 | 1 | 2955 tags=27%, list=15%, signal=32% |
| GSE21360_TERTIARY_VS_QUATERNARY_MEMORY_CD8_TCELL_DN | 167 | -0.494923 | -1.6817182 | 0 | 0.129 | 1 | 2545 tags=26%, list=13%, signal=30% |
| REACTOME_REGULATION_OF_HOMOTYPIC_CELL_CELL_ADHESION | 151 | -0.5007376 | -1.6817386 | 0 | 0.1299 | 1 | 3800 tags=32%, list=19%, signal=39% |
| GSE22601_DOUBLE_NEGATIVE_VS_CD4_SINGLE_POSITIVE_THYMOCYTE_DN | 179 | -0.4965651 | -1.6791081 | 0 | 0.13 | 1 | 3211 tags=27%, list=16%, signal=32% |
| GSE36888_UNTREATED_VS_IL2_TREATED_TCELL_17H_DN | 163 | -0.4941401 | -1.6794118 | 0 | 0.1305 | 1 | 2291 tags=25%, list=12%, signal=28% |
| **GSE43863_TFH_VS_LY6C_INT_CXCR5POS_MEMORY_CD4_TCELL_UP** | 175 | -0.4926492 | -1.6817857 | 0 | 0.1309 | 1 | 2220 tags=22%, list=11%, signal=25% |
| GSE11386_NAIVE_VS_MEMORY_BCELL_DN | 147 | -0.5038267 | -1.6829425 | 0 | 0.131 | 1 | 2552 tags=26%, list=13%, signal=29% |
| GSE15330_HSC_VS_GRANULOCYTE_MONOCYTE_PROGENITOR_DN | 173 | -0.4937239 | -1.6728307 | 0 | 0.1356 | 1 | 2417 tags=25%, list=12%, signal=28% |
| GSE43863_TH1_VS_TFH_MEMORY_CD4_TCELL_UP | 182 | -0.4893557 | -1.6714026 | 0 | 0.1369 | 1 | 2939 tags=24%, list=15%, signal=28% |
| GSE360_LOW_DOSE_B_MALAYI_VS_M_TUBERCULOSIS_DC_DN | 177 | -0.4930026 | -1.6667036 | 0 | 0.141 | 1 | 3286 tags=28%, list=17%, signal=33% |
| GSE2585_CTEC_VS_MTEC_THYMUS_UP | 174 | -0.4917016 | -1.6660384 | 0 | 0.1411 | 1 | 2418 tags=22%, list=12%, signal=25% |
| GSE10325_MYELOID_VS_LUPUS_MYELOID_DN | 167 | -0.4939452 | -1.6670439 | 0 | 0.1416 | 1 | 3093 tags=28%, list=16%, signal=32% |
| GSE37301_PRO_BCELL_VS_RAG2_KO_NK_CELL_UP | 162 | -0.5027287 | -1.6639516 | 0 | 0.144 | 1 | 3098 tags=25%, list=16%, signal=30% |
| GSE41176_WT_VS_TAK1_KO_ANTI_IGM_STIM_BCELL_24H_UP | 189 | -0.4832484 | -1.6492944 | 0 | 0.1496 | 1 | 2814 tags=27%, list=14%, signal=31% |
| GSE360_DC_VS_MAC_B_MALAYI_HIGH_DOSE_DN | 170 | -0.4869692 | -1.6544622 | 0 | 0.1498 | 1 | 2287 tags=21%, list=12%, signal=24% |
| GSE17721_CTRL_VS_GARDIQUIMOD_6H_BMDC_DN | 182 | -0.4902857 | -1.655725 | 0 | 0.1504 | 1 | 2979 tags=25%, list=15%, signal=29% |
| **GSE45365_NK_CELL_VS_CD11B_DC_DN** | 186 | -0.4815632 | -1.6530079 | 0 | 0.1504 | 1 | 2796 tags=24%, list=14%, signal=28% |
| **GSE9946_IMMATURE_VS_MATURE_STIMULATORY_DC_UP** | 120 | -0.5123062 | -1.6534888 | 0 | 0.1505 | 1 | 4135 tags=38%, list=21%, signal=48% |
| GSE37301_RAG2_KO_VS_RAG2_AND_ETS1_KO_NK_CELL_UP | 179 | -0.4812889 | -1.650882 | 0 | 0.1514 | 1 | 2803 tags=27%, list=14%, signal=31% |
| GSE14308_TH1_VS_NATURAL_TREG_DN | 179 | -0.4838785 | -1.640725 | 0 | 0.1519 | 1 | 3299 tags=28%, list=17%, signal=34% |
| **GSE22103_UNSTIM_VS_LPS_STIM_NEUTROPHIL_UP** | 179 | -0.4840863 | -1.6450564 | 0 | 0.1521 | 1 | 3457 tags=32%, list=18%, signal=38% |
| GSE28237_EARLY_VS_LATE_GC_BCELL_DN | 170 | -0.4790508 | -1.6444061 | 0 | 0.1523 | 1 | 3215 tags=28%, list=16%, signal=33% |
| GSE34156_TLR1_TLR2_LIGAND_VS_NOD2_AND_TLR1_TLR2_LIGAND_24H_TREATED_MONOCYTE_UP | 167 | -0.481961 | -1.6456684 | 0 | 0.1526 | 1 | 2713 tags=22%, list=14%, signal=25% |
| GOBP_XENOBIOTIC_METABOLIC_PROCESS | 90 | -0.5282633 | -1.643446 | 0 | 0.1531 | 1 | 2385 tags=26%, list=12%, signal=29% |
| GSE37416_CTRL_VS_12H_F_TULARENSIS_LVS_NEUTROPHIL_UP | 168 | -0.4853827 | -1.6413565 | 0 | 0.1534 | 1 | 3798 tags=32%, list=19%, signal=40% |
| GSE5503_LIVER_DC_VS_PLN_DC_ACTIVATED_ALLOGENIC_TCELL_DN | 176 | -0.4842066 | -1.6414758 | 0 | 0.1541 | 1 | 2618 tags=23%, list=13%, signal=27% |
| GSE29615_CTRL_VS_DAY3_LAIV_IFLU_VACCINE_PBMC_UP | 157 | -0.488225 | -1.6420205 | 0 | 0.1548 | 1 | 3383 tags=27%, list=17%, signal=33% |
| WP_NUCLEAR_RECEPTORS_METAPATHWAY | 266 | -0.4616605 | -1.6415695 | 0 | 0.1548 | 1 | 2766 tags=24%, list=14%, signal=28% |
| GSE7568_CTRL_VS_3H_TGFB_TREATED_MACROPHAGES_WITH_IL4_AND_DEXAMETHASONE_DN | 151 | -0.4866436 | -1.6280266 | 0 | 0.1673 | 1 | 3261 tags=26%, list=17%, signal=31% |
| GSE15330_HSC_VS_GRANULOCYTE_MONOCYTE_PROGENITOR_IKAROS_KO_UP | 184 | -0.4777926 | -1.6262579 | 0 | 0.1688 | 1 | 2900 tags=27%, list=15%, signal=32% |
| **GSE27859_MACROPHAGE_VS_CD11C_INT_F480_HI_MACROPHAGE_UP** | 161 | -0.4826246 | -1.6234496 | 0 | 0.1706 | 1 | 3418 tags=32%, list=17%, signal=38% |
| **GSE41867_MEMORY_VS_EXHAUSTED_CD8_TCELL_DAY30_LCMV_DN** | 176 | -0.4754674 | -1.6225969 | 0 | 0.1712 | 1 | 2769 tags=29%, list=14%, signal=33% |
| WP_MIRTARGETED_GENES_IN_LYMPHOCYTES | 405 | -0.4460768 | -1.6235658 | 0 | 0.1713 | 1 | 2627 tags=20%, list=13%, signal=23% |
| GOBP_GRANULOCYTE_CHEMOTAXIS | 105 | -0.5030352 | -1.6152612 | 0 | 0.1763 | 1 | 2863 tags=19%, list=15%, signal=22% |
| **GSE30971_CTRL_VS_LPS_STIM_MACROPHAGE_WBP7_HET_2H_UP** | 168 | -0.4786066 | -1.6100355 | 0 | 0.1771 | 1 | 2469 tags=26%, list=13%, signal=30% |
| **GSE2770_TGFB_AND_IL4_VS_IL4_TREATED_ACT_CD4_TCELL_48H_UP** | 167 | -0.4717965 | -1.6074966 | 0 | 0.1772 | 1 | 4170 tags=36%, list=21%, signal=45% |
| GOBP_GASTRULATION | 173 | -0.472843 | -1.6106273 | 0 | 0.1776 | 1 | 3681 tags=28%, list=19%, signal=35% |
| REACTOME_PLATELET_ACTIVATION_SIGNALING_AND_AGGREGATION | 250 | -0.461937 | -1.6109757 | 0 | 0.1778 | 1 | 3664 tags=30%, list=19%, signal=36% |
| REACTOME_CLASS_I_MHC_MEDIATED_ANTIGEN_PROCESSING_PRESENTATION | 344 | -0.4459391 | -1.6054085 | 0 | 0.178 |  | 3219 tags=26%, list=16%, signal=31% |
| GSE9988_ANTI_TREM1_VS_CTRL_TREATED_MONOCYTES_UP | 168 | -0.4793006 | -1.6110775 | 0 | 0.1783 |  | 2510 tags=23%, list=13%, signal=26% |
| **GSE2770_IL4_ACT_VS_ACT_CD4_TCELL_2H_DN** | 177 | -0.4699514 | -1.6082909 | 0 | 0.1789 |  | 2718 tags=25%, list=14%, signal=29% |
| **GSE37301_HEMATOPOIETIC_STEM_CELL_VS_LYMPHOID_PRIMED_MPP_DN** | 155 | -0.4798541 | -1.6004747 | 0 | 0.1811 |  | 2941 tags=26%, list=15%, signal=31% |
| **GSE27859_MACROPHAGE_VS_CD11C_INT_F480_INT_DC_UP** | 158 | -0.4708458 | -1.5789098 | 0 | 0.1834 |  | 3649 tags=31%, list=19%, signal=38% |
| **GSE22935_WT_VS_MYD88_KO_MACROPHAGE_12H_MBOVIS_BCG_STIM_DN** | 184 | -0.462687 | -1.5761817 | 0 | 0.1836 |  | 3826 tags=37%, list=20%, signal=45% |
| GSE22611_MUTANT_NOD2_TRANSDUCED_VS_CTRL_HEK293T_STIMULATED_WITH_MDP_6H_DN | 169 | -0.469194 | -1.5978358 | 0 | 0.1837 |  | 3688 tags=31%, list=19%, signal=38% |
| **GSE23505_UNTREATED_VS_4DAY_IL6_IL1_TGFB_TREATED_CD4_TCELL_UP** | 185 | -0.4603415 | -1.571389 | 0 | 0.1839 |  | 3486 tags=26%, list=18%, signal=31% |
| GSE17721_PAM3CSK4_VS_CPG_0.5H_BMDC_UP | 175 | -0.4658002 | -1.5836946 | 0 | 0.1839 |  | 2400 tags=27%, list=12%, signal=31% |
| GSE15330_LYMPHOID_MULTIPOTENT_VS_MEGAKARYOCYTE_ERYTHROID_PROGENITOR_IKAROS_KO_UP | 149 | -0.477353 | -1.5762947 | 0 | 0.1839 |  | 2842 tags=27%, list=14%, signal=31% |
| **GSE21063_CTRL_VS_ANTI_IGM_STIM_BCELL_NFATC1_KO_8H_UP** | 168 | -0.4623574 | -1.5792986 | 0 | 0.1839 |  | 3331 tags=33%, list=17%, signal=40% |

| **GSE26343_UNSTIM_VS_LPS_STIM_NFAT5_KO_MACROPHAGE_DN** | 189 | -0.4647725 | -1.5838885 |  | 0.1841 |  | 2897 tags=24%, list=15%, signal=28% |
| --- | --- | --- | --- | --- | --- | --- | --- |
| GSE13522_CTRL_VS_T_CRUZI_Y_STRAIN_INF_SKIN_IFNAR_KO_DN | 186 | -0.466114 | -1.5748328 | 0 | 0.1842 |  | 3087 tags=28%, list=16%, signal=33% |
| GSE13484_UNSTIM_VS_12H_YF17D_VACCINE_STIM_PBMC_DN | 166 | -0.4685083 | -1.5800848 | 0 | 0.1844 |  | 2977 tags=25%, list=15%, signal=29% |
| **GSE46606_IRF4_KO_VS_WT_CD40L_IL2_IL5_1DAY_STIMULATED_BCELL_DN** | 155 | -0.4683319 | -1.5842981 | 0 | 0.1847 |  | 2642 tags=24%, list=13%, signal=27% |
| **GSE3039_CD4_TCELL_VS_ALPHAALPHA_CD8_TCELL_UP** | 184 | -0.465323 | -1.5719994 | 0 | 0.185 |  | 3565 tags=35%, list=18%, signal=42% |
| GSE9006_TYPE_1_DIABETES_AT_DX_VS_1MONTH_POST_DX_PBMC_UP | 186 | -0.4582582 | -1.5826946 | 0 | 0.1852 |  | 2534 tags=21%, list=13%, signal=24% |
| **GSE25087_TREG_VS_TCONV_FETUS_UP** | 178 | -0.4626586 | -1.5892609 | 0 | 0.1858 | 1 | 2998 tags=26%, list=15%, signal=31% |
| GSE29618_BCELL_VS_PDC_DN | 179 | -0.4692373 | -1.5893732 | 0 | 0.1863 | 1 | 2983 tags=30%, list=15%, signal=35% |
| GSE25085_FETAL_LIVER_VS_FETAL_BM_SP4_THYMIC_IMPLANT_DN | 180 | -0.467193 | -1.5855027 | 0 | 0.187 | 1 | 2688 tags=24%, list=14%, signal=28% |
| GSE24142_ADULT_VS_FETAL_DN2_THYMOCYTE_DN | 172 | -0.4742669 | -1.5915132 | 0 | 0.1878 | 1 | 3263 tags=26%, list=17%, signal=31% |
| GSE19923_HEB_KO_VS_HEB_AND_E2A_KO_DP_THYMOCYTE_DN | 185 | -0.4552997 | -1.5529722 | 0 | 0.1971 | 1 | 3180 tags=25%, list=16%, signal=29% |
| **GSE9988_LPS_VS_LPS_AND_ANTI_TREM1_MONOCYTE_UP** | 176 | -0.4600544 | -1.5517818 | 0 | 0.1977 | 1 | 3484 tags=30%, list=18%, signal=36% |
| GSE22886_IGM_MEMORY_BCELL_VS_BM_PLASMA_CELL_DN | 176 | -0.4517735 | -1.5459452 | 0 | 0.1993 | 1 | 2532 tags=25%, list=13%, signal=28% |
| **GSE42724_NAIVE_VS_MEMORY_BCELL_DN** | 170 | -0.4536468 | -1.541243 | 0 | 0.2021 | 1 | 3249 tags=27%, list=17%, signal=32% |
| **GSE27786_BCELL_VS_NKCELL_DN** | 185 | -0.4485287 | -1.5395908 | 0 | 0.2029 | 1 | 2861 tags=23%, list=15%, signal=27% |
| **GSE8515_IL1_VS_IL6_4H_STIM_MAC_UP** | 180 | -0.4517432 | -1.5399348 | 0 | 0.2033 | 1 | 3200 tags=28%, list=16%, signal=34% |
| GSE22196_HEALTHY_VS_OBESE_MOUSE_SKIN_GAMMADELTA_TCELL_DN | 188 | -0.4434775 | -1.5350848 | 0 | 0.2043 | 1 | 3436 tags=29%, list=18%, signal=34% |
| GSE41867_LCMV_ARMSTRONG_VS_CLONE13_DAY8_EFFECTOR_CD8_TCELL_DN | 173 | -0.4534085 | -1.5365765 | 0 | 0.2045 | 1 | 3136 tags=26%, list=16%, signal=31% |
| GSE7852_THYMUS_VS_FAT_TCONV_DN | 185 | -0.4484896 | -1.5351253 | 0 | 0.2048 | 1 | 2128 tags=24%, list=11%, signal=26% |
| GSE20715_0H_VS_24H_OZONE_LUNG_DN | 183 | -0.4454373 | -1.5322078 | 0 | 0.2072 | 1 | 3563 tags=36%, list=18%, signal=44% |
| **GSE24634_TREG_VS_TCONV_POST_DAY10_IL4_CONVERSION_DN** | 174 | -0.4500895 | -1.5280633 | 0 | 0.2086 | 1 | 3172 tags=24%, list=16%, signal=28% |
| **GSE3920_IFNA_VS_IFNG_TREATED_ENDOTHELIAL_CELL_UP** | 160 | -0.4497573 | -1.5250311 | 0 | 0.2089 | 1 | 2943 tags=28%, list=15%, signal=33% |
| GSE37534_GW1929_VS_PIOGLITAZONE_TREATED_CD4_TCELL_PPARG1_FOXP3_TRANSDUCED_DN | 179 | -0.4500746 | -1.5222113 | 0 | 0.2089 | 1 | 4110 tags=34%, list=21%, signal=43% |
| GSE17721_CTRL_VS_GARDIQUIMOD_24H_BMDC_DN | 175 | -0.4483986 | -1.5172828 | 0 | 0.2091 | 1 | 3558 tags=29%, list=18%, signal=35% |
| GSE17721_LPS_VS_POLYIC_16H_BMDC_DN | 186 | -0.4385551 | -1.5139395 | 0 | 0.2124 | 1 | 3157 tags=25%, list=16%, signal=30% |
| GSE41176_WT_VS_TAK1_KO_ANTI_IGM_STIM_BCELL_24H_DN | 161 | -0.4436094 | -1.5026383 | 0 | 0.2193 | 1 | 2786 tags=23%, list=14%, signal=27% |
| GSE40273_GATA1_KO_VS_WT_TREG_DN | 176 | -0.4440568 | -1.4989593 | 0 | 0.2193 | 1 | 3544 tags=31%, list=18%, signal=38% |
| GSE7460_CTRL_VS_TGFB_TREATED_ACT_FOXP3_HET_TCONV_UP | 182 | -0.4347729 | -1.4999738 | 0 | 0.2201 | 1 | 3345 tags=29%, list=17%, signal=35% |
| GSE17721_ALL_VS_24H_PAM3CSK4_BMDC_UP | 177 | -0.4451636 | -1.4907553 | 0 | 0.2213 | 1 | 3699 tags=28%, list=19%, signal=34% |
| GOBP_CELL_SURFACE_RECEPTOR_PROTEIN_SERINE_THREONINE_KINASE_SIGNALING_PATHWAY | 374 | -0.4060647 | -1.483468 | 0 | 0.2237 | 1 | 3750 tags=28%, list=19%, signal=34% |
| **GSE6259_DEC205_POS_DC_VS_BCELL_DN** | 162 | -0.435001 | -1.467518 | 0 | 0.2367 | 1 | 2551 tags=21%, list=13%, signal=24% |
| GOBP_ACTIN_FILAMENT_ORGANIZATION | 418 | -0.3941789 | -1.4505787 | 0 | 0.2493 | 1 | 2627 tags=23%, list=13%, signal=26% |
| GSE14769_UNSTIM_VS_240MIN_LPS_BMDM_DN | 178 | -0.4281386 | -1.4506117 | 0 | 0.2495 | 1 | 3614 tags=28%, list=18%, signal=33% |
| **Enriched Gene-set-hallmarks in Vancomycin** | SIZE | ES | NES | NOM p-val FDR q-val | | FWER p-val | RANK AT MAX LEADING EDGE |
| WP_CHOLESTEROL_SYNTHESIS_DISORDERS | 16 | 0.8608486 | 2.1024868 | 0 0.0067827 | | 0.024 | 1571 tags=75%, list=8%, signal=81% |
| GOBP_CHOLESTEROL_BIOSYNTHETIC_PROCESS_VIA_DESMOSTEROL | 18 0.85177946 | | 2.1107616 | 0 0.0077315 | | 0.018 | 2271 tags=83%, list=12%, signal=94% |
| **REACTOME_DNA_STRAND_ELONGATION** | 32 0.7395364 | | 2.113225 | 0 0.0154631 | | 0.018 | 3148 tags=63%, list=16%, signal=74% |
| REACTOME_CHOLESTEROL_BIOSYNTHESIS | 23 0.79605603 | | 2.0573766 | 0 0.0154889 | | 0.071 | 2271 tags=74%, list=12%, signal=83% |
| WP_LEUCINE_ISOLEUCINE_AND_VALINE_METABOLISM | 23 0.7612895 | | 2.0002105 | 0 0.0177236 | | 0.2 | 1589 tags=52%, list=8%, signal=57% |
| WP_GENES_RELATED_TO_PRIMARY_CILIUM_DEVELOPMENT_BASED_ON_CRISPR | 84 0.58965874 | | 1.9942142 | 0 0.0182612 | | 0.225 | 3676 tags=45%, list=19%, signal=55% |
| **REACTOME_DISEASES_OF_DNA_REPAIR** | 49 0.65357506 | | 2.0359316 | 0 0.0182748 | | 0.106 | 3726 tags=51%, list=19%, signal=63% |
| **GSE15750_DAY6_VS_DAY10_EFF_CD8_TCELL_UP** | 177 0.5323209 | | 2.0013783 | 0 0.0192389 | | 0.196 | 3940 tags=47%, list=20%, signal=59% |
| GOBP_NON_PROTEINOGENIC_AMINO_ACID_CATABOLIC_PROCESS | 16 0.8125885 | | 2.0064995 | 0 0.0199082 | | 0.18 | 1851 tags=50%, list=9%, signal=55% |
| **GSE35543_IN_VITRO_ITREG_VS_CONVERTED_EX_ITREG_UP** | 184 0.53785646 | | 2.0100312 | 0 0.0211208 | | 0.167 | 3841 tags=42%, list=20%, signal=52% |
| GOBP_BRANCHED_CHAIN_AMINO_ACID_METABOLIC_PROCESS | 26 0.7395212 | | 2.014362 | 0 0.0231408 | | 0.157 | 1589 tags=46%, list=8%, signal=50% |
| REACTOME_MITOCHONDRIAL_FATTY_ACID_BETA_OXIDATION | 35 0.70133007 | | 1.9749712 | 0 0.0244933 | | 0.309 | 2805 tags=49%, list=14%, signal=57% |
| KEGG_VALINE_LEUCINE_AND_ISOLEUCINE_DEGRADATION | 41 0.66654897 | | 1.9596102 | 0 0.0278885 | | 0.36 | 1697 tags=46%, list=9%, signal=51% |
| **WP_DNA_REPAIR_PATHWAYS_FULL_NETWORK** | 113 0.5489193 | | 1.9302846 | 0 0.0396537 | | 0.486 | 4930 tags=54%, list=25%, signal=72% |
| **GOBP_DNA_TEMPLATED_DNA_REPLICATION_MAINTENANCE_OF_FIDELITY** | 59 0.60290134 | | 1.9115632 | 0 0.0417411 | | 0.603 | 4507 tags=53%, list=23%, signal=68% |
| **KEGG_DNA_REPLICATION** | 35 0.6552592 | | 1.9056987 0.00241546 0.0433225 | | | 0.641 | 4878 tags=66%, list=25%, signal=87% |
| REACTOME_CILIUM_ASSEMBLY | 181 0.51353097 | | 1.912433 0 0.0437174 | | | 0.598 | 4651 tags=44%, list=24%, signal=57% |
| REACTOME_INTRAFLAGELLAR_TRANSPORT | 42 0.6370369 | | 1.9128561 0 0.0461968 | | | 0.595 | 2384 tags=33%, list=12%, signal=38% |
| GOBP_CELL_COMMUNICATION_BY_ELECTRICAL_COUPLING | 35 0.6750272 | | 1.887416 0.00260417 0.047624 | | | 0.717 | 2157 tags=31%, list=11%, signal=35% |
| **GSE15750_DAY6_VS_DAY10_TRAF6KO_EFF_CD8_TCELL_UP** | 183 0.5126351 | | 1.9152615 0 0.0477431 | | | 0.583 | 4654 tags=57%, list=24%, signal=75% |
| REACTOME_SULFUR_AMINO_ACID_METABOLISM | 26 0.7092291 | | 1.8896116 0.00238664 0.0486105 | | | 0.708 | 2623 tags=46%, list=13%, signal=53% |
| **GOBP_MISMATCH_REPAIR** | 31 0.6903293 | | 1.8822061 0 0.0488976 | | | 0.734 | 3520 tags=65%, list=18%, signal=78% |
| REACTOME_VOLTAGE_GATED_POTASSIUM_CHANNELS | 38 0.6593351 | | 1.8919458 0 0.0498578 | | | 0.7 | 2326 tags=21%, list=12%, signal=24% |
| **GSE36476_CTRL_VS_TSST_ACT_72H_MEMORY_CD4_TCELL_OLD_DN** | 179 0.50323737 | | 1.8768629 0 0.0512224 | | | 0.769 | 4043 tags=43%, list=21%, signal=54% |
| GOBP_CENTRIOLE_ASSEMBLY | 44 0.63646924 | | 1.8725412 0 0.0522459 | | | 0.792 | 4496 tags=50%, list=23%, signal=65% |
| **REACTOME_MEIOTIC_RECOMBINATION** | 26 0.704429 | | 1.8645394 0 0.0537973 | | | 0.826 | 2042 tags=42%, list=10%, signal=47% |
| REACTOME_BRANCHED_CHAIN_AMINO_ACID_CATABOLISM | 21 0.73570037 | | 1.8658803 0.00227273 0.0547068 | | | 0.821 | 1589 tags=52%, list=8%, signal=57% |
| **REACTOME_TELOMERE_C_STRAND_LAGGING_STRAND_SYNTHESIS** | 32 0.65154225 | | 1.8522938 0.00503778 0.0554971 | | | 0.871 | 4828 tags=63%, list=25%, signal=83% |
| GOBP_INTRACILIARY_TRANSPORT | 42 0.625833 | | 1.8537316 0.00505051 0.0559319 | | | 0.864 | 3126 tags=45%, list=16%, signal=54% |
| GOBP_CELL_COMMUNICATION_INVOLVED_IN_CARDIAC_CONDUCTION | 55 0.58386534 | | 1.8587047 0 0.0562167 | | | 0.849 | 2157 tags=24%, list=11%, signal=26% |
| GOBP_MITOTIC_SISTER_CHROMATID_SEPARATION | 68 0.5678747 | | 1.854488 0 0.0571011 | | | 0.86 | 4921 tags=60%, list=25%, signal=80% |
| **REACTOME_LAGGING_STRAND_SYNTHESIS** | 20 0.7184338 | | 1.8472332 0.00491401 0.0578756 | | | 0.886 | 4827 tags=70%, list=25%, signal=93% |

| KEGG_GLYCINE_SERINE_AND_THREONINE_METABOLISM | 31 0.65052336 | 1.8425858 | 0.0580962 | 0.899 | 686 tags=19%, list=3%, signal=20% |
| --- | --- | --- | --- | --- | --- |
| GOBP_MEMBRANE_REPOLARIZATION_DURING_ACTION_POTENTIAL | 28 0.6644082 | 1.8438153 0.00236967 | 0.0589645 | 0.897 | 131 tags=11%, list=1%, signal=11% |
| **KEGG_MISMATCH_REPAIR** | 22 0.7217173 | 1.8331475 0.00470588 | 0.0619731 | 0.93 | 3127 tags=64%, list=16%, signal=76% |
| GOBP_AMINO_ACID_METABOLIC_PROCESS | 246 0.47034696 | 1.8350285 0 | 0.0625541 | 0.921 | 2511 tags=33%, list=13%, signal=37% |
| WP_BARDETBIEDL_SYNDROME | 74 0.56194675 | 1.8268486 0 | 0.0633996 | 0.952 | 2853 tags=35%, list=15%, signal=41% |
| GOBP_POTASSIUM_ION_HOMEOSTASIS | 42 0.61201143 | 1.8280126 0 | 0.0642287 | 0.947 | 1782 tags=21%, list=9%, signal=24% |
| **GOBP_DNA_TEMPLATED_DNA_REPLICATION** | 158 0.495132 | 1.8131363 0 | 0.0735025 | 0.974 | 3632 tags=41%, list=19%, signal=49% |
| GOBP_SULFUR_AMINO_ACID_BIOSYNTHETIC_PROCESS | 16 0.73932606 | 1.8079133 0.00244499 | 0.0760779 | 0.977 | 2242 tags=50%, list=11%, signal=56% |
| GOBP_ESTABLISHMENT_OF_PROTEIN_LOCALIZATION_TO_PEROXISOME | 20 0.71628284 | 1.8054677 0.00497512 | 0.076287 | 0.979 | 2954 tags=50%, list=15%, signal=59% |
| WP_7OXOC_AND_7BETAHC_PATHWAYS | 27 0.67416626 | 1.7893053 0.00234192 | 0.0775074 | 0.991 | 2158 tags=26%, list=11%, signal=29% |
| GOBP_REGULATION_OF_MEMBRANE_REPOLARIZATION | 32 0.6343619 | 1.7875938 0.00503778 | 0.0775886 | 0.992 | 131 tags=13%, list=1%, signal=13% |
| WP_MTHFR_DEFICIENCY | 22 0.6837183 | 1.7904389 0.00234742 | 0.0779117 | 0.991 | 1937 tags=27%, list=10%, signal=30% |
| GOBP_L_AMINO_ACID_METABOLIC_PROCESS | 158 0.48584527 | 1.7917246 0 | 0.0782293 | 0.991 | 1693 tags=28%, list=9%, signal=31% |
| **GOBP_DNA_SYNTHESIS_INVOLVED_IN_DNA_REPAIR** | 17 0.7311983 | 1.7991921 0.00451467 | 0.0787802 | 0.987 | 3629 tags=71%, list=18%, signal=87% |
| GOBP_NON_PROTEINOGENIC_AMINO_ACID_METABOLIC_PROCESS | 55 0.5738134 | 1.7947958 0 | 0.0791281 | 0.989 | 2149 tags=40%, list=11%, signal=45% |
| GOBP_AMINO_ACID_CATABOLIC_PROCESS | 98 0.5138842 | 1.7963914 0 | 0.0794753 | 0.988 | 1851 tags=35%, list=9%, signal=38% |
| GOBP_REGULATION_OF_PRESYNAPTIC_MEMBRANE_POTENTIAL | 24 0.6676646 | 1.8004829 0.00469484 | 0.079625 | 0.987 | 1248 tags=21%, list=6%, signal=22% |
| **REACTOME_HOMOLOGOUS_DNA_PAIRING_AND_STRAND_EXCHANGE** | 41 0.60236925 | 1.792069 0.00240964 | 0.0797349 | 0.991 | 3726 tags=44%, list=19%, signal=54% |
| GSE24634_TEFF_VS_TCONV_DAY7_IN_CULTURE_UP | 181 0.4718524 | 1.7506293 0 | 0.0848356 | 0.998 | 4329 tags=43%, list=22%, signal=54% |
| GOBP_METALLO_SULFUR_CLUSTER_ASSEMBLY | 24 0.66176856 | 1.7605201 0.00486618 | 0.0850573 | 0.998 | 2277 tags=33%, list=12%, signal=38% |
| GOBP_AMINO_ACID_BETAINE_METABOLIC_PROCESS | 19 0.7054366 | 1.7516805 0 | 0.0851508 | 0.998 | 1453 tags=37%, list=7%, signal=40% |
| GOBP_CELL_CELL_SIGNALING_INVOLVED_IN_CARDIAC_CONDUCTION | 28 0.64663726 | 1.7699789 0.00496278 | 0.0858088 | 0.997 | 601 tags=14%, list=3%, signal=15% |
| **REACTOME_MITOTIC_PROMETAPHASE** | 185 0.4689329 | 1.7580304 0 | 0.0860593 | 0.998 | 4850 tags=47%, list=25%, signal=62% |
| WP_PYRIMIDINE_METABOLISM_AND_RELATED_DISEASES | 19 0.6987645 | 1.7519466 0.00490196 | 0.0861684 | 0.998 | 4606 tags=68%, list=23%, signal=89% |
| **GSE5679_CTRL_VS_RARA_AGONIST_AM580_TREATED_DC_UP** | 179 0.46815646 | 1.7607495 0 | 0.0862445 | 0.998 | 3379 tags=34%, list=17%, signal=41% |
| **GOBP_INTERSTRAND_CROSS_LINK_REPAIR** | 36 0.6043306 | 1.7641215 0 | 0.0862654 | 0.998 | 4871 tags=58%, list=25%, signal=77% |
| GOBP_NUCLEOSIDE_METABOLIC_PROCESS | 37 0.61765707 | 1.7621264 0.0026455 | 0.0866397 | 0.998 | 4520 tags=62%, list=23%, signal=81% |
| **WP_NUCLEOTIDE_EXCISION_REPAIR_IN_XERODERMA_PIGMENTOSUM** | 57 0.5576891 | 1.7558482 0.00260417 | 0.0867316 | 0.998 | 4851 tags=56%, list=25%, signal=74% |
| **GSE25088_WT_VS_STAT6_KO_MACROPHAGE_IL4_STIM_DN** | 164 0.47756842 | 1.7705654 0 | 0.0868811 | 0.997 | 3806 tags=46%, list=19%, signal=56% |
| REACTOME_RESOLUTION_OF_D_LOOP_STRUCTURES | 33 0.60982096 | 1.7463892 0.00248139 | 0.0873563 | 0.998 | 5961 tags=55%, list=30%, signal=78% |
| GOBP_METHIONINE_METABOLIC_PROCESS | 16 0.7254089 | 1.7645442 0.00219298 | 0.0873774 | 0.998 | 2768 tags=63%, list=14%, signal=73% |
| REACTOME_NICOTINATE_METABOLISM | 27 0.6446205 | 1.7520158 0.00973236 | 0.0874571 | 0.998 | 2148 tags=37%, list=11%, signal=42% |
| REACTOME_ACTIVATION_OF_GENE_EXPRESSION_BY_SREBF_SREBP | 39 0.6038093 | 1.7713593 0 | 0.0877642 | 0.997 | 1571 tags=33%, list=8%, signal=36% |
| **GSE18893_TCONV_VS_TREG_24H_TNF_STIM_UP** | 179 0.4727757 | 1.752508 0 | 0.0883135 | 0.998 | 4037 tags=37%, list=21%, signal=47% |
| **GSE30962_PRIMARY_VS_SECONDARY_ACUTE_LCMV_INF_CD8_TCELL_UP** | 180 0.46501628 | 1.7650192 0 | 0.0884439 | 0.998 | 4718 tags=53%, list=24%, signal=69% |
| WP_FATTY_ACID_BIOSYNTHESIS | 20 0.69447654 | 1.774475 0.00471698 | 0.0884769 | 0.996 | 3721 tags=70%, list=19%, signal=86% |
| **WP_DNA_MISMATCH_REPAIR** | 23 0.6749112 | 1.771545 0.00234742 | 0.0893092 | 0.997 | 4827 tags=74%, list=25%, signal=98% |
| GOBP_CELL_COMMUNICATION_BY_ELECTRICAL_COUPLING_INVOLVED_IN_CARDIAC_CONDUCTION | 26 0.64925 | 1.740412 0.00487805 | 0.0911368 | 0.999 | 2157 tags=35%, list=11%, signal=39% |
| WP_TRYPTOPHAN_CATABOLISM_LEADING_TO_NAD_PRODUCTION | 15 0.7085057 | 1.7408097 0.00475059 | 0.092066 | 0.999 | 2115 tags=53%, list=11%, signal=60% |
| WP_CHOLESTEROL_BIOSYNTHESIS_PATHWAY_IN_HEPATOCYTES | 93 0.51238674 | 1.7285489 0 | 0.092272 | 1 | 2583 tags=34%, list=13%, signal=39% |
| **REACTOME_MITOTIC_SPINDLE_CHECKPOINT** | 105 0.4982138 | 1.738004 0 | 0.0924572 | 1 | 4839 tags=48%, list=25%, signal=63% |
| **REACTOME_RECRUITMENT_OF_MITOTIC_CENTROSOME_PROTEINS_AND_COMPLEXES** | 75 0.51608753 | 1.7292355 0.003003 | 0.0926331 | 1 | 4642 tags=47%, list=24%, signal=61% |
| GOBP_ALPHA_AMINO_ACID_METABOLIC_PROCESS | 187 0.45689273 | 1.7271124 0 | 0.0927263 | 1 | 1693 tags=27%, list=9%, signal=30% |
| REACTOME_AURKA_ACTIVATION_BY_TPX2 | 69 0.51776546 | 1.7251571 0.0028169 | 0.0933722 | 1 | 4642 tags=48%, list=24%, signal=62% |
| **REACTOME_POLYMERASE_SWITCHING_ON_THE_C_STRAND_OF_THE_TELOMERE** | 24 0.6490648 | 1.7295785 0.00242718 | 0.0934754 | 1 | 5464 tags=71%, list=28%, signal=98% |
| WP_JOUBERT_SYNDROME | 70 0.5236872 | 1.7234169 0.00276243 | 0.0940366 | 1 | 4381 tags=44%, list=22%, signal=57% |
| GOBP_ACYL_COA_METABOLIC_PROCESS | 98 0.49624383 | 1.7296727 0 | 0.0945722 | 1 | 2561 tags=33%, list=13%, signal=37% |
| **REACTOME_CHROMOSOME_MAINTENANCE** | 86 0.51462543 | 1.7345338 0.00282486 | 0.09476 | 1 | 5264 tags=53%, list=27%, signal=73% |
| KEGG_PROPANOATE_METABOLISM | 29 0.6275162 | 1.7300967 0.01196172 | 0.0953976 | 1 | 1697 tags=45%, list=9%, signal=49% |
| GOBP_REGULATION_OF_CELL_COMMUNICATION_BY_ELECTRICAL_COUPLING | 18 0.70628816 | 1.7301311 0.00473934 | 0.0966587 | 1 | 1992 tags=39%, list=10%, signal=43% |
| GOBP_LIGAND_GATED_ION_CHANNEL_SIGNALING_PATHWAY | 31 0.62303114 | 1.7197027 0.00251889 | 0.0970792 | 1 | 2135 tags=23%, list=11%, signal=25% |
| GOBP_L_AMINO_ACID_CATABOLIC_PROCESS | 78 0.51597166 | 1.7309597 0 | 0.0971934 | 1 | 1676 tags=35%, list=9%, signal=38% |
| WP_INTRAFLAGELLAR_TRANSPORT_PROTEINS_BINDING_TO_DYNEIN | 21 0.6748908 | 1.7171661 0.00689655 | 0.0972241 | 1 | 3591 tags=52%, list=18%, signal=64% |
| GOBP_ISOPRENOID_BIOSYNTHETIC_PROCESS | 24 0.6444713 | 1.7146802 0.00961539 | 0.0972879 | 1 | 1571 tags=38%, list=8%, signal=41% |
| REACTOME_RESOLUTION_OF_D_LOOP_STRUCTURES_THROUGH_SYNTHESIS_DEPENDENT_STRAND_ANNEALING_SDSA | 25 0.64008474 | 1.7157547 0.01459854 | 0.0974963 | 1 | 2042 tags=32%, list=10%, signal=36% |
| **GSE36476_CTRL_VS_TSST_ACT_72H_MEMORY_CD4_TCELL_YOUNG_DN** | 180 0.457915 | 1.7179561 0 | 0.097628 | 1 | 4476 tags=44%, list=23%, signal=57% |
| **REACTOME_HDR_THROUGH_HOMOLOGOUS_RECOMBINATION_HRR** | 66 0.5301476 | 1.7110076 0.00277778 | 0.0987023 |  | 4827 tags=48%, list=25%, signal=64% |
| GOBP_ATRIAL_CARDIAC_MUSCLE_CELL_TO_AV_NODE_CELL_COMMUNICATION | 16 0.71008354 | 1.7112974 0.01149425 | 0.0994718 |  | 68 tags=13%, list=0%, signal=13% |
| **GOBP_CHROMOSOME_ORGANIZATION_INVOLVED_IN_MEIOTIC_CELL_CYCLE** | 51 0.54048884 | 1.6998601 0.0080429 | 0.100783 |  | 3190 tags=39%, list=16%, signal=47% |
| **REACTOME_ORGANELLE_BIOGENESIS_AND_MAINTENANCE** | 250 0.4422464 | 1.7015613 0 | 0.1009548 |  | 3806 tags=35%, list=19%, signal=43% |
| **KEGG_NUCLEOTIDE_EXCISION_REPAIR** | 43 0.56880534 | 1.7067257 0.005 | 0.100959 |  | 5365 tags=60%, list=27%, signal=83% |
| GOBP_TERPENOID_BIOSYNTHETIC_PROCESS | 20 0.6730264 | 1.7074775 0.00947867 | 0.1013216 |  | 1571 tags=45%, list=8%, signal=49% |
| **GOBP_DNA_REPLICATION_INITIATION** | 41 0.5671979 | 1.700096 0.00524934 | 0.101485 |  | 3148 tags=39%, list=16%, signal=46% |
| **WP_NUCLEOTIDE_EXCISION_REPAIR** | 43 0.56880534 | 1.7018589 0.0026455 | 0.1016557 |  | 5365 tags=60%, list=27%, signal=83% |
| GOBP_CHEMOSENSORY_BEHAVIOR | 16 0.7063275 | 1.7025901 0.00851064 | 0.1018935 |  | 2641 tags=38%, list=13%, signal=43% |
| KEGG_PEROXISOME | 74 0.52013195 | 1.7026749 0.00573066 | 0.1028647 |  | 3156 tags=31%, list=16%, signal=37% |
| **GOBP_TELOMERE_MAINTENANCE_VIA_RECOMBINATION** | 15 0.7047426 | 1.7033103 0.00683371 | 0.1031114 |  | 2916 tags=53%, list=15%, signal=63% |

| WP_OXYSTEROLS_DERIVED_FROM_CHOLESTEROL | 47 0.5463369 | 1.6933237 0.00787402 | 0.1055133 |  | 2503 tags=28%, list=13%, signal=32% |
| --- | --- | --- | --- | --- | --- |
| REACTOME_POTASSIUM_CHANNELS | 95 0.49521193 | 1.6941777 0 | 0.105661 |  | 2532 tags=22%, list=13%, signal=25% |
| GOBP_SULFUR_AMINO_ACID_METABOLIC_PROCESS | 34 0.596042 | 1.6910511 0.00735294 | 0.1067537 |  | 2768 tags=47%, list=14%, signal=55% |
| REACTOME_MITOCHONDRIAL_TRNA_AMINOACYLATION | 18 0.686256 | 1.689628 0.0162413 | 0.1072421 |  | 2511 tags=50%, list=13%, signal=57% |
| REACTOME_ANCHORING_OF_THE_BASAL_BODY_TO_THE_PLASMA_MEMBRANE | 93 0.49000165 | 1.6862837 0 | 0.109825 |  | 4642 tags=45%, list=24%, signal=59% |
| **REACTOME_EXTENSION_OF_TELOMERES** | 49 0.5539719 | 1.6804444 0.00259067 | 0.1117486 |  | 5642 tags=61%, list=29%, signal=86% |
| GOBP_NEGATIVE_ADAPTATION_OF_SIGNALING_PATHWAY | 16 0.6834661 | 1.6789998 0.01445783 | 0.112385 | 1 | 1 tags=6%, list=0%, signal=6% |
| **REACTOME_MISMATCH_REPAIR** | 15 0.70207584 | 1.6807586 0.00902935 | 0.1125335 | 1 | 4827 tags=80%, list=25%, signal=106% |
| GOBP_MEMBRANE_REPOLARIZATION | 48 0.55088115 | 1.6773747 0.00259067 | 0.1129737 | 1 | 131 tags=8%, list=1%, signal=8% |
| **GOBP_MITOTIC_RECOMBINATION** | 26 0.6279623 | 1.6813709 0.00241546 | 0.1130243 | 1 | 5781 tags=73%, list=29%, signal=103% |
| **REACTOME_RECRUITMENT_OF_NUMA_TO_MITOTIC_CENTROSOMES** | 84 0.49566236 | 1.6821105 0 | 0.1132767 | 1 | 4642 tags=45%, list=24%, signal=59% |
| **GSE27241_WT_VS_RORGT_KO_TH17_POLARIZED_CD4_TCELL_UP** | 142 0.45903823 | 1.6734811 0 | 0.1161943 | 1 | 3832 tags=46%, list=20%, signal=56% |
| **GSE19374_UNINF_VS_LISTERIA_INFECTED_MACROPHAGE_UP** | 156 0.4449007 | 1.6662182 0 | 0.1183541 | 1 | 3097 tags=25%, list=16%, signal=29% |
| WP_MITOCHONDRIAL_FATTY_ACID_OXIDATION_DISORDERS | 19 0.66296107 | 1.6675029 0.01234568 | 0.1189079 | 1 | 2805 tags=37%, list=14%, signal=43% |
| GOBP_SMOOTH_MUSCLE_CONTRACTION | 94 0.47843954 | 1.6647891 0.00295858 | 0.1189519 | 1 | 1813 tags=21%, list=9%, signal=23% |
| **WP_DNA_REPLICATION** | 41 0.5665399 | 1.6663202 0.00258398 | 0.1192687 | 1 | 4878 tags=59%, list=25%, signal=78% |
| **GSE22919_RESTING_VS_IL2_IL12_IL15_STIM_NK_CELL_DN** | 178 0.44632557 | 1.6679589 0 | 0.1193688 | 1 | 3547 tags=33%, list=18%, signal=39% |
| **GSE27241_WT_VS_RORGT_KO_TH17_POLARIZED_CD4_TCELL_DN** | 155 0.44941047 | 1.6625382 0 | 0.1194291 | 1 | 4223 tags=38%, list=22%, signal=48% |
| **REACTOME_RESOLUTION_OF_SISTER_CHROMATID_COHESION** | 113 0.47639224 | 1.6687101 0.00282486 | 0.1195301 | 1 | 4850 tags=47%, list=25%, signal=62% |
| KEGG_NICOTINATE_AND_NICOTINAMIDE_METABOLISM | 23 0.6216398 | 1.6627332 0.01234568 | 0.1201308 | 1 | 2115 tags=43%, list=11%, signal=49% |
| GOBP_PEROXISOMAL_TRANSPORT | 22 0.6425442 | 1.6690013 0.01463415 | 0.1202543 | 1 | 2954 tags=41%, list=15%, signal=48% |
| **GOBP_MITOTIC_SISTER_CHROMATID_SEGREGATION** | 187 0.44419628 | 1.6608983 0 | 0.1203309 | 1 | 4496 tags=47%, list=23%, signal=60% |
| REACTOME_ACTIVATION_OF_ATR_IN_RESPONSE_TO_REPLICATION_STRESS | 37 0.57437575 | 1.6579027 0.00950119 | 0.1226701 | 1 | 3455 tags=51%, list=18%, signal=62% |
| **GSE15930_NAIVE_VS_72H_IN_VITRO_STIM_CD8_TCELL_DN** | 182 0.43589693 | 1.6569754 0 | 0.1228882 | 1 | 3360 tags=34%, list=17%, signal=41% |
| REACTOME_PCNA_DEPENDENT_LONG_PATCH_BASE_EXCISION_REPAIR | 21 0.65826046 | 1.656195 0.00941176 | 0.1229184 | 1 | 4827 tags=67%, list=25%, signal=88% |
| WP_DNA_IRDAMAGE_AND_CELLULAR_RESPONSE_VIA_ATR | 76 0.5034431 | 1.655241 0 | 0.1230011 | 1 | 3460 tags=41%, list=18%, signal=49% |
| **GOBP_POSITIVE_REGULATION_OF_MITOTIC_SISTER_CHROMATID_SEPARATION** | 22 0.6399041 | 1.6414995 0.007109 | 0.1264008 | 1 | 5499 tags=73%, list=28%, signal=101% |
| GOBP_MUCUS_SECRETION | 17 0.6601613 | 1.6418549 0.02137767 | 0.1269063 | 1 | 206 tags=12%, list=1%, signal=12% |
| **GSE39110_DAY3_VS_DAY6_POST_IMMUNIZATION_CD8_TCELL_DN** | 180 0.44285545 | 1.6447977 0 | 0.1273091 | 1 | 4727 tags=49%, list=24%, signal=65% |
| GOBP_MODIFIED_AMINO_ACID_CATABOLIC_PROCESS | 30 0.60018057 | 1.6422863 0.00917431 | 0.1273234 | 1 | 837 tags=27%, list=4%, signal=28% |
| **GOBP_DNA_STRAND_ELONGATION_INVOLVED_IN_DNA_REPLICATION** | 15 0.69611394 | 1.6430595 0.01643192 | 0.1273772 | 1 | 4878 tags=73%, list=25%, signal=98% |
| **REACTOME_PROCESSIVE_SYNTHESIS_ON_THE_C_STRAND_OF_THE_TELOMERE** | 18 0.6675301 | 1.6464025 0.00696056 | 0.1274052 | 1 | 5961 tags=78%, list=30%, signal=112% |
| GOBP_PROTEIN_TRANSMEMBRANE_IMPORT_INTO_INTRACELLULAR_ORGANELLE | 15 0.686237 | 1.6364638 0.03090508 | 0.1275454 | 1 | 2954 tags=53%, list=15%, signal=63% |
| GOBP_ACTION_POTENTIAL | 162 0.44376552 | 1.6386728 0 | 0.1277821 | 1 | 2532 tags=20%, list=13%, signal=23% |
| GOBP_CARDIAC_MUSCLE_CELL_ACTION_POTENTIAL_INVOLVED_IN_CONTRACTION | 54 0.5211637 | 1.6354495 0.00555556 | 0.127889 | 1 | 601 tags=11%, list=3%, signal=11% |
| **REACTOME_MEIOSIS** | 57 0.53129715 | 1.6450659 0.0052356 | 0.1279449 | 1 | 2962 tags=35%, list=15%, signal=41% |
| **GSE2770_IL12_AND_TGFB_VS_IL4_TREATED_ACT_CD4_TCELL_6H_DN** | 165 0.44134605 | 1.6475675 0 | 0.1280637 | 1 | 2814 tags=23%, list=14%, signal=27% |
| **REACTOME_INHIBITION_OF_THE_PROTEOLYTIC_ACTIVITY_OF_APC_C_REQUIRED_FOR_THE_ONSET_OF_ANAPHASE_BY_MITOTIC_SPIND** | 21 0.6315206 | 1.6432961 0.0127551 | 0.1280903 | 1 | 4276 tags=52%, list=22%, signal=67% |
| **GSE21360_PRIMARY_VS_SECONDARY_MEMORY_CD8_TCELL_UP** | 158 0.44318473 | 1.636727 0 | 0.1281496 | 1 | 2466 tags=22%, list=13%, signal=25% |
| **GOBP_CENTROSOME_DUPLICATION** | 75 0.48174512 | 1.6391388 0 | 0.1281545 | 1 | 4496 tags=41%, list=23%, signal=53% |
| WP_SEROTONIN_AND_ANXIETY | 16 0.68668044 | 1.6465111 0.01856148 | 0.128253 | 1 | 2841 tags=31%, list=14%, signal=37% |
| GOBP_STEROL_BIOSYNTHETIC_PROCESS | 60 0.51809025 | 1.6490808 0.00269542 | 0.1283422 | 1 | 2271 tags=38%, list=12%, signal=43% |
| **GSE39556_CD8A_DC_VS_NK_CELL_MOUSE_3H_POST_POLYIC_INJ_UP** | 180 0.44007477 | 1.6371111 0 | 0.1286077 | 1 | 4227 tags=45%, list=22%, signal=57% |
| **REACTOME_TELOMERE_MAINTENANCE** | 63 0.5108028 | 1.6333193 0.01595745 | 0.1286878 | 1 | 5027 tags=49%, list=26%, signal=66% |
| REACTOME_PROCESSIVE_SYNTHESIS_ON_THE_LAGGING_STRAND | 15 0.716752 | 1.6477427 0.01459854 | 0.1288275 | 1 | 4827 tags=67%, list=25%, signal=88% |
| **REACTOME_ACTIVATION_OF_THE_PRE_REPLICATIVE_COMPLEX** | 32 0.57568717 | 1.6315522 0.00485437 | 0.1289085 | 1 | 3629 tags=47%, list=18%, signal=57% |
| **GSE21063_WT_VS_NFATC1_KO_8H_ANTI_IGM_STIM_BCELL_UP** | 174 0.44934043 | 1.6492335 0.00330033 | 0.1291665 | 1 | 4590 tags=44%, list=23%, signal=57% |
| **GSE5679_CTRL_VS_PPARG_LIGAND_ROSIGLITAZONE_TREATED_DC_UP** | 178 0.43726793 | 1.6319562 0 | 0.1292794 | 1 | 3790 tags=35%, list=19%, signal=43% |
| WP_MITOCHONDRIAL_LONG_CHAIN_FATTY_ACID_BETAOXIDATION | 17 0.6649629 | 1.6334058 0.02083333 | 0.1294453 | 1 | 2548 tags=35%, list=13%, signal=41% |
| **GOBP_REGULATION_OF_DNA_TEMPLATED_DNA_REPLICATION_INITIATION** | 20 0.6509918 | 1.6267211 0.01131222 | 0.1327055 | 1 | 3148 tags=50%, list=16%, signal=59% |
| GOBP_G_PROTEIN_COUPLED_RECEPTOR_INTERNALIZATION | 15 0.68750465 | 1.6267707 0.01826484 | 0.1335245 | 1 | 1 tags=7%, list=0%, signal=7% |
| **GSE21063_CTRL_VS_ANTI_IGM_STIM_BCELL_16H_UP** | 174 0.43816903 | 1.6251554 0 | 0.133724 | 1 | 3461 tags=33%, list=18%, signal=39% |
| KEGG_AMINOACYL_TRNA_BIOSYNTHESIS | 22 0.6181507 | 1.623503 0.02212389 | 0.133935 | 1 | 2778 tags=41%, list=14%, signal=48% |
| GOBP_ACETYL_COA_METABOLIC_PROCESS | 42 0.5570971 | 1.6240097 0.01044386 | 0.1342518 | 1 | 2448 tags=38%, list=12%, signal=43% |
| GOBP_NADPPLUS_METABOLIC_PROCESS | 32 0.57188106 | 1.6161575 0.00721154 | 0.1420739 | 1 | 2283 tags=44%, list=12%, signal=49% |
| **GSE40274_FOXP3_VS_FOXP3_AND_EOS_TRANSDUCED_ACTIVATED_CD4_TCELL_DN** | 182 0.43179658 | 1.6140459 0 | 0.1428613 | 1 | 3790 tags=36%, list=19%, signal=45% |
| REACTOME_TRNA_AMINOACYLATION | 24 0.5997231 | 1.6143812 0.02375297 | 0.1434302 |  | 2511 tags=38%, list=13%, signal=43% |
| GSE19888_ADENOSINE_A3R_ACT_VS_A3R_ACT_WITH_A3R_INH_PRETREATMENT_IN_MAST_CELL_UP | 163 0.43371928 | 1.6098536 0 | 0.1473619 |  | 2791 tags=28%, list=14%, signal=33% |
| **KEGG_HOMOLOGOUS_RECOMBINATION** | 25 0.6038821 | 1.6087419 0.01666667 | 0.1478974 |  | 5205 tags=56%, list=27%, signal=76% |
| **GSE24634_TREG_VS_TCONV_POST_DAY7_IL4_CONVERSION_UP** | 179 0.4314271 | 1.6059321 0 | 0.1504889 |  | 4878 tags=41%, list=25%, signal=54% |
| GOBP_POTASSIUM_ION_TRANSPORT | 211 0.42076433 | 1.5984428 0 | 0.1509108 |  | 2701 tags=20%, list=14%, signal=23% |
| GSE25085_FETAL_LIVER_VS_ADULT_BM_SP4_THYMIC_IMPLANT_DN | 175 0.42731556 | 1.5975792 0 | 0.1511849 |  | 5279 tags=54%, list=27%, signal=74% |
| REACTOME_METABOLISM_OF_AMINO_ACIDS_AND_DERIVATIVES | 326 0.4031252 | 1.5999739 0 | 0.1516189 |  | 2482 tags=19%, list=13%, signal=21% |
| GOBP_ORGANIC_ACID_CATABOLIC_PROCESS | 222 0.4179588 | 1.5985833 0 | 0.1516434 |  | 2883 tags=32%, list=15%, signal=37% |
| WP_TRANSSULFURATION_ONECARBON_METABOLISM_AND_RELATED_PATHWAYS | 67 0.5023139 | 1.6026161 0.00542005 | 0.1518552 |  | 1937 tags=33%, list=10%, signal=36% |
| GOBP_POSITIVE_REGULATION_OF_SODIUM_ION_TRANSMEMBRANE_TRANSPORT | 18 0.64828867 | 1.6032699 0.01674641 | 0.1518958 |  | 921 tags=28%, list=5%, signal=29% |
| REACTOME_HDR_THROUGH_SINGLE_STRAND_ANNEALING_SSA | 36 0.5550338 | 1.6008971 0.01228501 | 0.1522528 |  | 3455 tags=39%, list=18%, signal=47% |

| **GOBP_CHROMOSOME_SEPARATION** | 85 0.47416914 | 1.6016088 0.00289017 | 0.1522804 |  | 4496 tags=54%, list=23%, signal=70% |
| --- | --- | --- | --- | --- | --- |
| GOBP_NEGATIVE_REGULATION_OF_CHROMOSOME_ORGANIZATION | 94 0.46992892 | 1.6001482 0.00560224 | 0.1523052 |  | 5264 tags=53%, list=27%, signal=72% |
| **GSE17974_IL4_AND_ANTI_IL12_VS_UNTREATED_4H_ACT_CD4_TCELL_DN** | 122 0.45027152 | 1.5987192 0.00298507 | 0.1523898 |  | 2616 tags=25%, list=13%, signal=29% |
| **GOBP_REGULATION_OF_CENTRIOLE_REPLICATION** | 21 0.61542 | 1.6035373 0.02233251 | 0.1525186 |  | 2510 tags=29%, list=13%, signal=33% |
| KEGG_STEROID_BIOSYNTHESIS | 16 0.6526421 | 1.5953887 0.02364066 | 0.1530007 |  | 2271 tags=56%, list=12%, signal=64% |
| **GSE18791_CTRL_VS_NEWCASTLE_VIRUS_DC_4H_UP** | 169 0.43257862 | 1.5944592 0 | 0.1533413 |  | 3657 tags=32%, list=19%, signal=39% |
| REACTOME_RESOLUTION_OF_AP_SITES_VIA_THE_MULTIPLE_NUCLEOTIDE_PATCH_REPLACEMENT_PATHWAY | 24 0.6062973 | 1.5906768 0.01162791 | 0.1546554 | 1 | 4827 tags=58%, list=25%, signal=77% |
| GOBP_DETECTION_OF_TEMPERATURE_STIMULUS_INVOLVED_IN_SENSORY_PERCEPTION_OF_PAIN | 19 0.63804233 | 1.5919625 0.02050114 | 0.1547275 | 1 | 3287 tags=37%, list=17%, signal=44% |
| **GSE18791_UNSTIM_VS_NEWCATSLE_VIRUS_DC_2H_UP** | 115 0.44876492 | 1.5921196 0.00276243 | 0.155343 | 1 | 3646 tags=35%, list=19%, signal=42% |
| REACTOME_CITRIC_ACID_CYCLE_TCA_CYCLE | 25 0.5965475 | 1.5907469 0.02857143 | 0.155474 | 1 | 4197 tags=56%, list=21%, signal=71% |
| **GSE13547_CTRL_VS_ANTI_IGM_STIM_BCELL_12H_UP** | 173 0.4266724 | 1.5891945 0 | 0.1558401 | 1 | 3832 tags=42%, list=20%, signal=51% |
| KEGG_BETA_ALANINE_METABOLISM | 22 0.60904783 | 1.5870398 0.02347418 | 0.1571038 | 1 | 2149 tags=45%, list=11%, signal=51% |
| **GOBP_PURINE_DEOXYRIBONUCLEOTIDE_METABOLIC_PROCESS** | 17 0.649238 | 1.5871046 0.01834862 | 0.1578766 | 1 | 960 tags=24%, list=5%, signal=25% |
| GOBP_DETECTION_OF_TEMPERATURE_STIMULUS | 25 0.58891034 | 1.5855423 0.02050114 | 0.1581314 | 1 | 2090 tags=20%, list=11%, signal=22% |
| **REACTOME_REGULATION_OF_PLK1_ACTIVITY_AT_G2_M_TRANSITION** | 83 0.4687673 | 1.5835001 0 | 0.1583098 | 1 | 4642 tags=43%, list=24%, signal=57% |
| GOBP_PIGMENT_GRANULE_LOCALIZATION | 24 0.5961564 | 1.583686 0.02030457 | 0.1589061 | 1 | 3533 tags=54%, list=18%, signal=66% |
| REACTOME_MITOCHONDRIAL_PROTEIN_DEGRADATION | 79 0.4778059 | 1.5812948 0.00288184 | 0.1595175 | 1 | 3961 tags=41%, list=20%, signal=51% |
| GSE14413_UNSTIM_VS_IFNB_STIM_RAW264_CELLS_DN | 102 0.4521026 | 1.5838901 0.0027933 | 0.1595406 | 1 | 1613 tags=15%, list=8%, signal=16% |
| GOBP_NEGATIVE_REGULATION_OF_POTASSIUM_ION_TRANSPORT | 25 0.60168254 | 1.5777194 0.02046036 | 0.1598669 | 1 | 1185 tags=24%, list=6%, signal=26% |
| **REACTOME_BASE_EXCISION_REPAIR** | 43 0.52449036 | 1.5815419 0.02088773 | 0.1600549 | 1 | 5636 tags=63%, list=29%, signal=88% |
| REACTOME_RESOLUTION_OF_ABASIC_SITES_AP_SITES | 37 0.5392041 | 1.5781976 0.01477833 | 0.1601374 | 1 | 4930 tags=59%, list=25%, signal=79% |
| GOBP_REGULATION_OF_VASCULOGENESIS | 19 0.6253533 | 1.5784062 0.0258216 | 0.1606813 | 1 | 2044 tags=26%, list=10%, signal=29% |
| GOBP_GLYCOSYL_COMPOUND_CATABOLIC_PROCESS | 17 0.65365356 | 1.5751718 0.02727273 | 0.1608307 | 1 | 3974 tags=53%, list=20%, signal=66% |
| GOBP_REGULATION_OF_SYNAPTIC_VESICLE_MEMBRANE_ORGANIZATION | 22 0.5894787 | 1.5788237 0.02758621 | 0.160973 | 1 | 1081 tags=23%, list=6%, signal=24% |
| KEGG_PYRIMIDINE_METABOLISM | 92 0.4607773 | 1.5755675 0.01169591 | 0.1611237 | 1 | 4849 tags=42%, list=25%, signal=56% |
| REACTOME_REGULATION_OF_CHOLESTEROL_BIOSYNTHESIS_BY_SREBP_SREBF | 52 0.5101402 | 1.5733213 0 | 0.1616053 | 1 | 1571 tags=27%, list=8%, signal=29% |
| GOBP_REGULATION_OF_CARDIAC_MUSCLE_CELL_MEMBRANE_REPOLARIZATION | 23 0.61779195 | 1.5788555 0.02392345 | 0.1617987 | 1 | 18 tags=9%, list=0%, signal=9% |
| **GSE22601_DOUBLE_POSITIVE_VS_CD4_SINGLE_POSITIVE_THYMOCYTE_DN** | 182 0.41747865 | 1.5737884 0 | 0.1618067 | 1 | 4008 tags=40%, list=20%, signal=49% |
| **REACTOME_G1_S_SPECIFIC_TRANSCRIPTION** | 29 0.5774189 | 1.5756302 0.03703704 | 0.1618862 | 1 | 4977 tags=45%, list=25%, signal=60% |
| **GOBP_HOMOLOGOUS_RECOMBINATION** | 60 0.48432228 | 1.5713959 0 | 0.1634916 | 1 | 2707 tags=28%, list=14%, signal=33% |
| GSE2405_S_AUREUS_VS_UNTREATED_NEUTROPHIL_DN | 183 0.4166682 | 1.569146 0 | 0.1655934 | 1 | 3127 tags=34%, list=16%, signal=40% |
| **GOBP_PROTEIN_LOCALIZATION_TO_CHROMOSOME_CENTROMERIC_REGION** | 22 0.6088777 | 1.5665064 0.01662708 | 0.1683933 | 1 | 4921 tags=64%, list=25%, signal=85% |
| REACTOME_FANCONI_ANEMIA_PATHWAY | 33 0.5509194 | 1.5636175 0.02941177 | 0.1707639 | 1 | 3455 tags=36%, list=18%, signal=44% |
| GOBP_DETECTION_OF_STIMULUS_INVOLVED_IN_SENSORY_PERCEPTION_OF_PAIN | 27 0.58948314 | 1.5629423 0.01941748 | 0.1708886 | 1 | 3287 tags=30%, list=17%, signal=36% |
| GOBP_CARDIAC_MUSCLE_CELL_MEMBRANE_REPOLARIZATION | 34 0.5529655 | 1.5623573 0.02672606 | 0.1709201 | 1 | 18 tags=6%, list=0%, signal=6% |
| WP_METABOLIC_EPILEPTIC_DISORDERS | 84 0.46257973 | 1.5638186 0.00557103 | 0.1713108 | 1 | 1676 tags=23%, list=9%, signal=25% |
| **GOBP_MITOTIC_INTRA_S_DNA_DAMAGE_CHECKPOINT_SIGNALING** | 19 0.6209725 | 1.560129 0.04618938 | 0.1733384 | 1 | 3215 tags=42%, list=16%, signal=50% |
| GSE2405_0H_VS_12H_A_PHAGOCYTOPHILUM_STIM_NEUTROPHIL_DN | 178 0.4136484 | 1.5558169 0.00348432 | 0.1779328 | 1 | 3400 tags=35%, list=17%, signal=42% |
| GOBP_POSITIVE_REGULATION_OF_SODIUM_ION_TRANSPORT | 31 0.5680984 | 1.555992 0.01809955 | 0.1785525 | 1 | 921 tags=19%, list=5%, signal=20% |
| **GSE14415_NATURAL_TREG_VS_TCONV_DN** | 167 0.41664994 | 1.5534145 0 | 0.1804603 | 1 | 3810 tags=39%, list=19%, signal=48% |
| GOBP_DNA_BIOSYNTHETIC_PROCESS | 148 0.41966376 | 1.5511683 0 | 0.1827538 | 1 | 3790 tags=36%, list=19%, signal=45% |
| GOBP_INTRACELLULAR_POTASSIUM_ION_HOMEOSTASIS | 23 0.5898668 | 1.5503029 0.02921348 | 0.1832092 | 1 | 1782 tags=22%, list=9%, signal=24% |
| **GSE5589_WT_VS_IL10_KO_LPS_STIM_MACROPHAGE_45MIN_UP** | 178 0.4146295 | 1.5453887 0.00355872 | 0.1896869 | 1 | 3850 tags=37%, list=20%, signal=45% |
| REACTOME_BBSOME_MEDIATED_CARGO_TARGETING_TO_CILIUM | 23 0.5922164 | 1.5444556 0.02962963 | 0.190194 | 1 | 3998 tags=39%, list=20%, signal=49% |
| GSE13485_DAY3_VS_DAY21_YF17D_VACCINE_PBMC_DN | 107 0.44182366 | 1.5435486 0.00895522 | 0.1904607 | 1 | 1609 tags=13%, list=8%, signal=14% |
| **GOBP_REGULATION_OF_MITOTIC_SISTER_CHROMATID_SEGREGATION** | 61 0.49161386 | 1.5377699 0.0136612 | 0.1955731 | 1 | 5262 tags=57%, list=27%, signal=78% |
| REACTOME_RND1_GTPASE_CYCLE | 42 0.5174908 | 1.5378795 0.0026455 | 0.1963573 | 1 | 3087 tags=36%, list=16%, signal=42% |
| WP_FANCONI_ANEMIA | 41 0.5287976 | 1.5381687 0.02717391 | 0.196877 | 1 | 3553 tags=41%, list=18%, signal=51% |
| **GSE40274_CTRL_VS_FOXP3_TRANSDUCED_ACTIVATED_CD4_TCELL_UP** | 185 0.4115516 | 1.5382696 0 | 0.1976168 | 1 | 3194 tags=36%, list=16%, signal=43% |
| **GOBP_NUCLEAR_CHROMOSOME_SEGREGATION** | 293 0.3936781 | 1.5329515 0 | 0.2023866 | 1 | 4496 tags=42%, list=23%, signal=53% |
| GOBP_INTRACELLULAR_SODIUM_ION_HOMEOSTASIS | 31 0.54921836 | 1.5301036 0.03544304 | 0.2050145 | 1 | 1988 tags=19%, list=10%, signal=22% |
| GOBP_MELANOCYTE_DIFFERENTIATION | 21 0.601424 | 1.5305116 0.04222222 | 0.2053231 | 1 | 2966 tags=38%, list=15%, signal=45% |
| WP_ETHER_LIPID_BIOSYNTHESIS | 18 0.61798686 | 1.52926 0.04136253 | 0.2054975 | 1 | 2954 tags=39%, list=15%, signal=46% |
| GSE29618_PRE_VS_DAY7_FLU_VACCINE_MONOCYTE_UP | 146 0.42543906 | 1.5273001 0.00692042 | 0.2057222 | 1 | 2391 tags=22%, list=12%, signal=25% |
| REACTOME_EGR2_AND_SOX10_MEDIATED_INITIATION_OF_SCHWANN_CELL_MYELINATION | 24 0.5725485 | 1.5275339 0.03058824 | 0.2063212 | 1 | 4152 tags=50%, list=21%, signal=63% |
| **GOBP_DNA_REPLICATION_CHECKPOINT_SIGNALING** | 25 0.5865365 | 1.5279932 0.03846154 | 0.2065719 | 1 | 3105 tags=40%, list=16%, signal=47% |
| KEGG_BUTANOATE_METABOLISM | 31 0.5492612 | 1.522242 0.02722772 | 0.2121539 |  | 2548 tags=45%, list=13%, signal=52% |
| GOBP_CALCIUM_ION_IMPORT | 72 0.46007428 | 1.5224217 0.00795756 | 0.2128052 |  | 1911 tags=17%, list=10%, signal=18% |
| REACTOME_METABOLISM_OF_STEROIDS | 132 0.42295772 | 1.5196247 0.00291545 | 0.2144962 |  | 2583 tags=24%, list=13%, signal=28% |
| **GOBP_ATTACHMENT_OF_SPINDLE_MICROTUBULES_TO_KINETOCHORE** | 50 0.48695335 | 1.520124 0.01980198 | 0.2145015 |  | 3940 tags=46%, list=20%, signal=57% |
| **REACTOME_PHOSPHORYLATION_OF_THE_APC_C** | 20 0.60096455 | 1.5188099 0.03349282 | 0.2148824 |  | 5135 tags=55%, list=26%, signal=74% |
| **REACTOME_S_PHASE** | 147 0.41693935 | 1.5153799 0.00664452 | 0.2200151 |  | 5125 tags=39%, list=26%, signal=53% |
| GOBP_POSITIVE_REGULATION_OF_DEFENSE_RESPONSE_TO_VIRUS_BY_HOST | 23 0.57078433 | 1.5129869 0.02941177 | 0.2231376 |  | 1710 tags=13%, list=9%, signal=14% |
| GOBP_POSITIVE_REGULATION_OF_SMOOTH_MUSCLE_CELL_DIFFERENTIATION | 19 0.59309465 | 1.5105479 0.04418605 | 0.2233745 |  | 2499 tags=37%, list=13%, signal=42% |
| **GOBP_T_CELL_MEDIATED_CYTOTOXICITY** | 36 0.528089 | 1.509862 0.01995013 | 0.2235937 |  | 1022 tags=14%, list=5%, signal=15% |
| WP_H19_RBE2F1_AND_CDKBETACATENIN_IN_COLORECTAL_CANCER | 15 0.64769065 | 1.5108535 0.05411765 | 0.2237816 |  | 2095 tags=40%, list=11%, signal=45% |
| **REACTOME_ABERRANT_REGULATION_OF_MITOTIC_EXIT_IN_CANCER_DUE_TO_RB1_DEFECTS** | 20 0.5878486 | 1.511744 0.03818616 | 0.2242607 |  | 5125 tags=50%, list=26%, signal=68% |

| GOBP_CARDIAC_MUSCLE_CELL_ACTION_POTENTIAL | 78 0.45235175 | 1.5110233 0.01136364 | 0.2244637 |  | 601 tags=9%, list=3%, signal=9% |
| --- | --- | --- | --- | --- | --- |
| WP_SUDDEN_INFANT_DEATH_SYNDROME_SIDS_SUSCEPTIBILITY_PATHWAYS | 140 0.41343135 | 1.5085728 0.00309598 | 0.2249991 |  | 2292 tags=21%, list=12%, signal=23% |
| GOBP_NEPHRON_TUBULE_FORMATION | 20 0.5864951 | 1.5074333 0.04285714 | 0.2259451 |  | 2022 tags=25%, list=10%, signal=28% |
| GSE37532_WT_VS_PPARG_KO_VISCERAL_ADIPOSE_TISSUE_TREG_UP | 169 0.4041161 | 1.5060252 0 | 0.2264793 |  | 3860 tags=40%, list=20%, signal=50% |
| **GOBP_REGULATION_OF_DNA_TEMPLATED_DNA_REPLICATION** | 46 0.48829216 | 1.5063319 0.02835052 | 0.226911 |  | 3404 tags=39%, list=17%, signal=47% |
| GOBP_MITOCHONDRIAL_TRANSMEMBRANE_TRANSPORT | 24 0.56038016 | 1.5018662 0.03864734 | 0.2293668 |  | 1087 tags=17%, list=6%, signal=18% |
| **GSE36476_CTRL_VS_TSST_ACT_40H_MEMORY_CD4_TCELL_YOUNG_DN** | 180 0.40209505 | 1.5012356 0.00357143 | 0.2295093 | 1 | 3307 tags=30%, list=17%, signal=36% |
| **GOBP_HOMOLOGOUS_CHROMOSOME_PAIRING_AT_MEIOSIS** | 39 0.5080151 | 1.5021589 0.02863962 | 0.2297276 | 1 | 4044 tags=41%, list=21%, signal=52% |
| GOBP_ACTIN_FILAMENT_BASED_MOVEMENT | 130 0.4135173 | 1.5027039 0.00325733 | 0.2297726 | 1 | 809 tags=11%, list=4%, signal=11% |
| GOBP_FATTY_ACID_DERIVATIVE_BIOSYNTHETIC_PROCESS | 43 0.5022136 | 1.5031464 0.02356021 | 0.2299337 | 1 | 2234 tags=28%, list=11%, signal=31% |
| GSE45365_CTRL_VS_MCMV_INFECTION_NK_CELL_UP | 174 0.40468216 | 1.5036658 0.00996678 | 0.2299828 | 1 | 2077 tags=24%, list=11%, signal=27% |
| GOBP_AORTA_DEVELOPMENT | 66 0.4637953 | 1.500385 0.02739726 | 0.2300228 | 1 | 2152 tags=26%, list=11%, signal=29% |
| **GOBP_DOUBLE_STRAND_BREAK_REPAIR_VIA_NONHOMOLOGOUS_END_JOINING** | 61 0.46950287 | 1.4983826 0.01265823 | 0.2307573 | 1 | 4058 tags=38%, list=21%, signal=47% |
| GOBP_AORTA_MORPHOGENESIS | 34 0.5077893 | 1.4987743 0.02926829 | 0.2310322 | 1 | 2821 tags=35%, list=14%, signal=41% |
| GOBP_EPITHELIAL_TO_MESENCHYMAL_TRANSITION_INVOLVED_IN_ENDOCARDIAL_CUSHION_FORMATION | 18 0.6118787 | 1.4993058 0.05450237 | 0.2310372 | 1 | 2223 tags=33%, list=11%, signal=38% |
| REACTOME_PEROXISOMAL_PROTEIN_IMPORT | 58 0.47010946 | 1.4956478 0.033241 | 0.2325115 | 1 | 2734 tags=29%, list=14%, signal=34% |
| GOBP_NEURON_FATE_COMMITMENT | 55 0.48086917 | 1.4948001 0.01794872 | 0.2330886 | 1 | 1011 tags=15%, list=5%, signal=15% |
| WP_ONECARBON_METABOLISM | 29 0.5414261 | 1.4957442 0.04466501 | 0.2332946 | 1 | 1937 tags=31%, list=10%, signal=34% |
| GOBP_SMALL_MOLECULE_CATABOLIC_PROCESS | 326 0.3733661 | 1.4938108 0 | 0.2339449 | 1 | 2435 tags=25%, list=12%, signal=28% |
| GSE24671_BAKIMULC_VS_SENDAI_VIRUS_INFECTED_MOUSE_SPLENOCYTES_UP | 182 0.39904478 | 1.4957619 0 | 0.2341856 | 1 | 2524 tags=26%, list=13%, signal=30% |
| REACTOME_PURINE_CATABOLISM | 17 0.6234598 | 1.4911367 0.03820225 | 0.2366733 | 1 | 1694 tags=29%, list=9%, signal=32% |
| GOBP_SODIUM_ION_EXPORT_ACROSS_PLASMA_MEMBRANE | 21 0.58630985 | 1.491616 0.02830189 | 0.236804 | 1 | 921 tags=14%, list=5%, signal=15% |
| GOBP_METABOLIC_COMPOUND_SALVAGE | 40 0.50526226 | 1.4899842 0.03084833 | 0.2376498 | 1 | 4050 tags=43%, list=21%, signal=53% |
| REACTOME_FATTY_ACID_METABOLISM | 141 0.40718156 | 1.4884222 0.0096463 | 0.2395253 | 1 | 2805 tags=28%, list=14%, signal=33% |
| GOBP_CALCIUM_ION_IMPORT_ACROSS_PLASMA_MEMBRANE | 36 0.505518 | 1.4867611 0.04207921 | 0.2398358 | 1 | 1911 tags=17%, list=10%, signal=18% |
| **REACTOME_HOMOLOGY_DIRECTED_REPAIR** | 98 0.44027913 | 1.4876969 0.01416431 | 0.2399361 | 1 | 4827 tags=41%, list=25%, signal=54% |
| GOBP_POSITIVE_REGULATION_OF_GLUCONEOGENESIS | 21 0.5751732 | 1.4871386 0.06203474 | 0.2400163 | 1 | 2629 tags=24%, list=13%, signal=27% |
| GOBP_GLYCOSYL_COMPOUND_BIOSYNTHETIC_PROCESS | 18 0.5986916 | 1.4861017 0.04634146 | 0.2401395 | 1 | 4050 tags=44%, list=21%, signal=56% |
| GOBP_CELLULAR_RESPONSE_TO_FATTY_ACID | 34 0.5161687 | 1.484473 0.03962704 | 0.2421426 | 1 | 1144 tags=24%, list=6%, signal=25% |
| WP_NAD_METABOLISM | 16 0.60261774 | 1.4825588 0.05569007 | 0.2429473 | 1 | 2115 tags=31%, list=11%, signal=35% |
| GOBP_AMINO_ACID_ACTIVATION | 28 0.53975165 | 1.482645 0.04207921 | 0.2436763 | 1 | 2511 tags=32%, list=13%, signal=37% |
| **REACTOME_CELL_CYCLE_CHECKPOINTS** | 240 0.3817779 | 1.4829962 0 | 0.2439728 | 1 | 3559 tags=30%, list=18%, signal=36% |
| GOBP_ESTABLISHMENT_OF_PIGMENT_GRANULE_LOCALIZATION | 22 0.5762296 | 1.4794651 0.05092593 | 0.2457944 | 1 | 3533 tags=50%, list=18%, signal=61% |
| GOBP_PYRIDINE_CONTAINING_COMPOUND_BIOSYNTHETIC_PROCESS | 21 0.57427484 | 1.4804062 0.05479452 | 0.2458668 | 1 | 2468 tags=48%, list=13%, signal=54% |
| GOBP_SISTER_CHROMATID_SEGREGATION | 226 0.3889394 | 1.479819 0.00332226 | 0.246031 | 1 | 4496 tags=42%, list=23%, signal=54% |
| **GSE36476_CTRL_VS_TSST_ACT_40H_MEMORY_CD4_TCELL_OLD_DN** | 181 0.3920579 | 1.478095 0.00325733 | 0.2464749 | 1 | 4232 tags=42%, list=22%, signal=53% |
| WP_CHOLESTEROL_METABOLISM_WITH_BLOCH_AND_KANDUTSCHRUSSELL_PATHWAYS | 44 0.50003713 | 1.4784665 0.04864865 | 0.2466599 | 1 | 2271 tags=41%, list=12%, signal=46% |
| GOBP_PROTEIN_LOCALIZATION_TO_CILIUM | 78 0.44225544 | 1.4761945 0.01457726 | 0.249119 | 1 | 3591 tags=36%, list=18%, signal=44% |
| WP_CILIOPATHIES | 149 0.40472993 | 1.4736277 0.0031348 | 0.2493135 | 1 | 2971 tags=29%, list=15%, signal=34% |

**Supp. Table 2.** Taxa table of 1025-NR microbiome at visit 1 (V1)

| **ptid_visit** | **family** | **phylum** | **class** | **order** | **genus** | **species** |
| --- | --- | --- | --- | --- | --- | --- |
| 840001025-V1 | Bacteroidaceae | Bacteroidetes | Bacteroidia | Bacteroidales | Bacteroides |  |
| 840001025-V1 | Lachnospiraceae | Firmicutes | Clostridia | Clostridiales | Blautia |  |
| 840001025-V1 | Bacteroidaceae | Bacteroidetes | Bacteroidia | Bacteroidales | Bacteroides |  |
| 840001025-V1 | Bacteroidaceae | Bacteroidetes | Bacteroidia | Bacteroidales | Bacteroides |  |
| 840001025-V1 | Bacteroidaceae | Bacteroidetes | Bacteroidia | Bacteroidales | Bacteroides | Bacteroides_eggerthii_DSM_20697 |
| 840001025-V1 | Burkholderiaceae | Proteobacteria | Gammaproteobacteria | Betaproteobacteriales | Parasutterella |  |
| 840001025-V1 | Lachnospiraceae | Firmicutes | Clostridia | Clostridiales | Blautia |  |
| 840001025-V1 | Rikenellaceae | Bacteroidetes | Bacteroidia | Bacteroidales | Alistipes |  |
| 840001025-V1 | Rikenellaceae | Bacteroidetes | Bacteroidia | Bacteroidales | Alistipes |  |
| 840001025-V1 | Ruminococcaceae | Firmicutes | Clostridia | Clostridiales | Faecalibacterium |  |
| 840001025-V1 | Coriobacteriaceae | Actinobacteria | Coriobacteriia | Coriobacteriales | Collinsella |  |
| 840001025-V1 | Bacteroidaceae | Bacteroidetes | Bacteroidia | Bacteroidales | Bacteroides | Bacteroides_massiliensis_B84634_=_Timone_84634_=_DSM_17679_=_JCM_13223 |
| 840001025-V1 | Lachnospiraceae | Firmicutes | Clostridia | Clostridiales | Dorea |  |
| 840001025-V1 | Acidaminococcaceae | Firmicutes | Negativicutes | Selenomonadales | Phascolarctobacterium |  |
| 840001025-V1 | Ruminococcaceae | Firmicutes | Clostridia | Clostridiales | Faecalibacterium |  |
| 840001025-V1 | Bacteroidaceae | Bacteroidetes | Bacteroidia | Bacteroidales | Bacteroides | Bacteroides_thetaiotaomicron |
| 840001025-V1 | Lachnospiraceae | Firmicutes | Clostridia | Clostridiales | Lachnoclostridium | uncultured_Firmicutes_bacterium |
| 840001025-V1 | Ruminococcaceae | Firmicutes | Clostridia | Clostridiales | Ruminococcus_2 | uncultured_bacterium |
| 840001025-V1 | Bacteroidaceae | Bacteroidetes | Bacteroidia | Bacteroidales | Bacteroides |  |
| 840001025-V1 | Lachnospiraceae | Firmicutes | Clostridia | Clostridiales | Fusicatenibacter |  |
| 840001025-V1 | Lachnospiraceae | Firmicutes | Clostridia | Clostridiales | Roseburia | uncultured_Roseburia_sp. |
| 840001025-V1 | Lachnospiraceae | Firmicutes | Clostridia | Clostridiales | Blautia |  |
| 840001025-V1 | Bacteroidaceae | Bacteroidetes | Bacteroidia | Bacteroidales | Bacteroides |  |
| 840001025-V1 | Lachnospiraceae | Firmicutes | Clostridia | Clostridiales | Blautia |  |
| 840001025-V1 | Lachnospiraceae | Firmicutes | Clostridia | Clostridiales | Coprococcus_3 |  |
| 840001025-V1 | Ruminococcaceae | Firmicutes | Clostridia | Clostridiales | Oscillibacter | uncultured_organism |
| 840001025-V1 | Bacteroidaceae | Bacteroidetes | Bacteroidia | Bacteroidales | Bacteroides | Bacteroides_eggerthii_DSM_20697 |
| 840001025-V1 | Lachnospiraceae | Firmicutes | Clostridia | Clostridiales | [Ruminococcus]_torques_group | uncultured_organism |
| 840001025-V1 | Enterobacteriaceae | Proteobacteria | Gammaproteobacteria | Enterobacteriales | Escherichia-Shigella |  |
| 840001025-V1 | Erysipelotrichaceae | Firmicutes | Erysipelotrichia | Erysipelotrichales | Catenibacterium |  |
| 840001025-V1 | Ruminococcaceae | Firmicutes | Clostridia | Clostridiales | Ruminococcaceae_UCG-002 | uncultured_organism |
| 840001025-V1 | Lachnospiraceae | Firmicutes | Clostridia | Clostridiales | Blautia |  |
| 840001025-V1 | Ruminococcaceae | Firmicutes | Clostridia | Clostridiales | Subdoligranulum |  |
| 840001025-V1 | Lachnospiraceae | Firmicutes | Clostridia | Clostridiales | Lachnoclostridium |  |
| 840001025-V1 | Ruminococcaceae | Firmicutes | Clostridia | Clostridiales | [Eubacterium]_coprostanoligenes_group | unidentified |
| 840001025-V1 | Tannerellaceae | Bacteroidetes | Bacteroidia | Bacteroidales | Parabacteroides | Parabacteroides_distasonis |
| 840001025-V1 | Bacteroidaceae | Bacteroidetes | Bacteroidia | Bacteroidales | Bacteroides |  |
| 840001025-V1 | Ruminococcaceae | Firmicutes | Clostridia | Clostridiales | Subdoligranulum |  |
| 840001025-V1 | Tannerellaceae | Bacteroidetes | Bacteroidia | Bacteroidales | Parabacteroides |  |
| 840001025-V1 | Lachnospiraceae | Firmicutes | Clostridia | Clostridiales | Anaerostipes |  |
| 840001025-V1 | Ruminococcaceae | Firmicutes | Clostridia | Clostridiales | Faecalibacterium |  |
| 840001025-V1 | Lachnospiraceae | Firmicutes | Clostridia | Clostridiales | Coprococcus_1 |  |
| 840001025-V1 | Ruminococcaceae | Firmicutes | Clostridia | Clostridiales | Faecalibacterium |  |
| 840001025-V1 | Ruminococcaceae | Firmicutes | Clostridia | Clostridiales | Flavonifractor |  |
| 840001025-V1 | Bacteroidaceae | Bacteroidetes | Bacteroidia | Bacteroidales | Bacteroides |  |
| 840001025-V1 | Lachnospiraceae | Firmicutes | Clostridia | Clostridiales | Lachnoclostridium |  |
| 840001025-V1 | Lachnospiraceae | Firmicutes | Clostridia | Clostridiales | Tyzzerella |  |
| 840001025-V1 | Lachnospiraceae | Firmicutes | Clostridia | Clostridiales |  |  |
| 840001025-V1 | Lachnospiraceae | Firmicutes | Clostridia | Clostridiales | [Ruminococcus]_gauvreauii_group |  |
| 840001025-V1 | Lachnospiraceae | Firmicutes | Clostridia | Clostridiales | Blautia |  |
| 840001025-V1 | Lachnospiraceae | Firmicutes | Clostridia | Clostridiales | [Ruminococcus]_torques_group | Ruminococcus_lactaris_CC59_002D |
| 840001025-V1 | Lachnospiraceae | Firmicutes | Clostridia | Clostridiales | Blautia |  |
| 840001025-V1 | Lachnospiraceae | Firmicutes | Clostridia | Clostridiales | uncultured |  |
| 840001025-V1 | Lachnospiraceae | Firmicutes | Clostridia | Clostridiales | Blautia |  |
| 840001025-V1 | Ruminococcaceae | Firmicutes | Clostridia | Clostridiales | Faecalibacterium |  |
| 840001025-V1 | Lachnospiraceae | Firmicutes | Clostridia | Clostridiales |  |  |
| 840001025-V1 | Ruminococcaceae | Firmicutes | Clostridia | Clostridiales | Faecalibacterium |  |
| 840001025-V1 | Lachnospiraceae | Firmicutes | Clostridia | Clostridiales | Blautia |  |
| 840001025-V1 | Marinifilaceae | Bacteroidetes | Bacteroidia | Bacteroidales | Butyricimonas |  |
| 840001025-V1 | Lachnospiraceae | Firmicutes | Clostridia | Clostridiales | Anaerostipes |  |
| 840001025-V1 | Ruminococcaceae | Firmicutes | Clostridia | Clostridiales | Faecalibacterium |  |
| 840001025-V1 | Ruminococcaceae | Firmicutes | Clostridia | Clostridiales | Faecalibacterium |  |
| 840001025-V1 | Bacteroidaceae | Bacteroidetes | Bacteroidia | Bacteroidales | Bacteroides |  |
| 840001025-V1 | Ruminococcaceae | Firmicutes | Clostridia | Clostridiales | Negativibacillus | uncultured_bacterium |
| 840001025-V1 | Lachnospiraceae | Firmicutes | Clostridia | Clostridiales | uncultured | uncultured_organism |
| 840001025-V1 | Rikenellaceae | Bacteroidetes | Bacteroidia | Bacteroidales | Alistipes | Bacteroides_sp._Smarlab_3302398 |
| 840001025-V1 | Lachnospiraceae | Firmicutes | Clostridia | Clostridiales |  |  |
| 840001025-V1 | Eggerthellaceae | Actinobacteria | Coriobacteriia | Coriobacteriales | Slackia |  |
| 840001025-V1 | Bacteroidaceae | Bacteroidetes | Bacteroidia | Bacteroidales | Bacteroides |  |
| 840001025-V1 | Lachnospiraceae | Firmicutes | Clostridia | Clostridiales | Agathobacter |  |
| 840001025-V1 | Lachnospiraceae | Firmicutes | Clostridia | Clostridiales | Blautia | Blautia_hydrogenotrophica |
| 840001025-V1 | Streptococcaceae | Firmicutes | Bacilli | Lactobacillales | Streptococcus | Streptococcus_salivarius_subsp._thermophilus |
| 840001025-V1 | Lachnospiraceae | Firmicutes | Clostridia | Clostridiales |  |  |
| 840001025-V1 | Lachnospiraceae | Firmicutes | Clostridia | Clostridiales | [Eubacterium]_eligens_group |  |
| 840001025-V1 | Bacteroidaceae | Bacteroidetes | Bacteroidia | Bacteroidales | Bacteroides | Bacteroides_sp._HPS0048 |
| 840001025-V1 | Ruminococcaceae | Firmicutes | Clostridia | Clostridiales | Subdoligranulum |  |
| 840001025-V1 | Lachnospiraceae | Firmicutes | Clostridia | Clostridiales | Lachnospiraceae_ND3007_group | metagenome |
| 840001025-V1 | Eubacteriaceae | Firmicutes | Clostridia | Clostridiales | Eubacterium |  |
| 840001025-V1 | Erysipelotrichaceae | Firmicutes | Erysipelotrichia | Erysipelotrichales | Catenibacterium |  |
| 840001025-V1 | Ruminococcaceae | Firmicutes | Clostridia | Clostridiales | Ruminiclostridium_9 |  |
| 840001025-V1 | Bacteroidaceae | Bacteroidetes | Bacteroidia | Bacteroidales | Bacteroides |  |
| 840001025-V1 | Lachnospiraceae | Firmicutes | Clostridia | Clostridiales | Lachnoclostridium |  |
| 840001025-V1 | Ruminococcaceae | Firmicutes | Clostridia | Clostridiales | Ruminiclostridium_5 | uncultured_organism |
| 840001025-V1 | Ruminococcaceae | Firmicutes | Clostridia | Clostridiales | Ruminococcaceae_UCG-003 |  |
| 840001025-V1 | Ruminococcaceae | Firmicutes | Clostridia | Clostridiales | Butyricicoccus |  |
| 840001025-V1 | Ruminococcaceae | Firmicutes | Clostridia | Clostridiales | DTU089 | uncultured_organism |
| 840001025-V1 | Lachnospiraceae | Firmicutes | Clostridia | Clostridiales | Sellimonas | uncultured_bacterium |
| 840001025-V1 | Bacteroidaceae | Bacteroidetes | Bacteroidia | Bacteroidales | Bacteroides | Bacteroides_ovatus |
| 840001025-V1 | Ruminococcaceae | Firmicutes | Clostridia | Clostridiales | Faecalibacterium | metagenome |
| 840001025-V1 | Lachnospiraceae | Firmicutes | Clostridia | Clostridiales | Blautia |  |
| 840001025-V1 | Ruminococcaceae | Firmicutes | Clostridia | Clostridiales | Ruminococcus_1 | uncultured_organism |
| 840001025-V1 | Lachnospiraceae | Firmicutes | Clostridia | Clostridiales | uncultured | uncultured_Eubacterium_sp. |
| 840001025-V1 | Lachnospiraceae | Firmicutes | Clostridia | Clostridiales | [Ruminococcus]_gnavus_group |  |
| 840001025-V1 | Eggerthellaceae | Actinobacteria | Coriobacteriia | Coriobacteriales | Eggerthella |  |
| 840001025-V1 | Lachnospiraceae | Firmicutes | Clostridia | Clostridiales | Roseburia |  |
| 840001025-V1 | Tannerellaceae | Bacteroidetes | Bacteroidia | Bacteroidales | Parabacteroides |  |
| 840001025-V1 | Ruminococcaceae | Firmicutes | Clostridia | Clostridiales | uncultured | Clostridium_phoceensis |
| 840001025-V1 | Lachnospiraceae | Firmicutes | Clostridia | Clostridiales | uncultured |  |
| 840001025-V1 | Lachnospiraceae | Firmicutes | Clostridia | Clostridiales | Blautia |  |
| 840001025-V1 | Lachnospiraceae | Firmicutes | Clostridia | Clostridiales | Lachnospiraceae_UCG-010 | uncultured_organism |
| 840001025-V1 | Ruminococcaceae | Firmicutes | Clostridia | Clostridiales | Oscillibacter | human_gut_metagenome |
| 840001025-V1 | Ruminococcaceae | Firmicutes | Clostridia | Clostridiales | uncultured |  |
| 840001025-V1 | Lachnospiraceae | Firmicutes | Clostridia | Clostridiales | [Eubacterium]_hallii_group | uncultured_bacterium |
| 840001025-V1 | Ruminococcaceae | Firmicutes | Clostridia | Clostridiales | Ruminococcaceae_UCG-013 | uncultured_organism |
| 840001025-V1 | Lachnospiraceae | Firmicutes | Clostridia | Clostridiales | GCA-900066575 |  |
| 840001025-V1 | Bacteroidaceae | Bacteroidetes | Bacteroidia | Bacteroidales | Bacteroides | Bacteroides_stercorirosoris |
| 840001025-V1 | Lachnospiraceae | Firmicutes | Clostridia | Clostridiales |  |  |
| 840001025-V1 | Streptococcaceae | Firmicutes | Bacilli | Lactobacillales | Streptococcus |  |
| 840001025-V1 | Ruminococcaceae | Firmicutes | Clostridia | Clostridiales | UBA1819 | uncultured_organism |
| 840001025-V1 | Christensenellaceae | Firmicutes | Clostridia | Clostridiales | Christensenellaceae_R-7_group | uncultured_marine_bacterium |
| 840001025-V1 | Ruminococcaceae | Firmicutes | Clostridia | Clostridiales | Ruminococcaceae_UCG-013 | gut_metagenome |
| 840001025-V1 | Ruminococcaceae | Firmicutes | Clostridia | Clostridiales | Ruminiclostridium_5 | gut_metagenome |
| 840001025-V1 | Ruminococcaceae | Firmicutes | Clostridia | Clostridiales | Ruminiclostridium_9 | uncultured_organism |
| 840001025-V1 | Erysipelotrichaceae | Firmicutes | Erysipelotrichia | Erysipelotrichales | Holdemania |  |
| 840001025-V1 | Actinomycetaceae | Actinobacteria | Actinobacteria | Actinomycetales | Actinomyces |  |
| 840001025-V1 | Lachnospiraceae | Firmicutes | Clostridia | Clostridiales | [Eubacterium]_ventriosum_group |  |
| 840001025-V1 | Coriobacteriales_Incertae_Sedis | Actinobacteria | Coriobacteriia | Coriobacteriales | uncultured | gut_metagenome |
| 840001025-V1 | Family_XIII | Firmicutes | Clostridia | Clostridiales | Family_XIII_AD3011_group | uncultured_bacterium |
| 840001025-V1 | Peptostreptococcaceae | Firmicutes | Clostridia | Clostridiales | Romboutsia |  |
| 840001025-V1 | Ruminococcaceae | Firmicutes | Clostridia | Clostridiales | Ruminiclostridium | Massilioclostridium_coli |
| 840001025-V1 | Lachnospiraceae | Firmicutes | Clostridia | Clostridiales |  |  |
| 840001025-V1 | Lachnospiraceae | Firmicutes | Clostridia | Clostridiales | Lachnoclostridium |  |
| 840001025-V1 | Ruminococcaceae | Firmicutes | Clostridia | Clostridiales | Ruminiclostridium_5 | uncultured_bacterium |
| 840001025-V1 | Ruminococcaceae | Firmicutes | Clostridia | Clostridiales | Butyricicoccus | uncultured_organism |
| 840001025-V1 | Christensenellaceae | Firmicutes | Clostridia | Clostridiales | uncultured | uncultured_bacterium |
| 840001025-V1 | Eggerthellaceae | Actinobacteria | Coriobacteriia | Coriobacteriales | Gordonibacter | unidentified |
| 840001025-V1 | Lachnospiraceae | Firmicutes | Clostridia | Clostridiales | Blautia |  |
| 840001025-V1 | Clostridiaceae_1 | Firmicutes | Clostridia | Clostridiales | Clostridium_sensu_stricto_1 |  |
| 840001025-V1 | Eubacteriaceae | Firmicutes | Clostridia | Clostridiales | Anaerofustis | unidentified |

**Supp. Table 3.** Taxa table of 1014-R microbiome at visit 1 (V1)

| **ptid_visit** | **family** | **phylum** | **class** | **order** | **genus** | **species** |
| --- | --- | --- | --- | --- | --- | --- |
| 840001014-V1 | Bifidobacteriaceae | Actinobacteria | Actinobacteria | Bifidobacteriales | Bifidobacterium |  |
| 840001014-V1 | Enterobacteriaceae | Proteobacteria | Gammaproteobacteria | Enterobacteriales |  |  |
| 840001014-V1 | Lachnospiraceae | Firmicutes | Clostridia | Clostridiales | Fusicatenibacter |  |
| 840001014-V1 | Ruminococcaceae | Firmicutes | Clostridia | Clostridiales | Faecalibacterium |  |
| 840001014-V1 | Ruminococcaceae | Firmicutes | Clostridia | Clostridiales | Faecalibacterium |  |
| 840001014-V1 | Lachnospiraceae | Firmicutes | Clostridia | Clostridiales | Blautia |  |
| 840001014-V1 | Erysipelotrichaceae | Firmicutes | Erysipelotrichia | Erysipelotrichales | Erysipelotrichaceae_UCG-003 |  |
| 840001014-V1 | Lachnospiraceae | Firmicutes | Clostridia | Clostridiales | Agathobacter |  |
| 840001014-V1 | Streptococcaceae | Firmicutes | Bacilli | Lactobacillales | Streptococcus | Streptococcus_salivarius_subsp._thermophilus |
| 840001014-V1 | Lachnospiraceae | Firmicutes | Clostridia | Clostridiales | [Ruminococcus]_torques_group |  |
| 840001014-V1 | Clostridiaceae_1 | Firmicutes | Clostridia | Clostridiales | Clostridium_sensu_stricto_1 |  |
| 840001014-V1 | Lachnospiraceae | Firmicutes | Clostridia | Clostridiales | Lachnospira |  |
| 840001014-V1 | Lachnospiraceae | Firmicutes | Clostridia | Clostridiales | Dorea |  |
| 840001014-V1 | Lachnospiraceae | Firmicutes | Clostridia | Clostridiales | Lachnospiraceae_ND3007_group | metagenome |
| 840001014-V1 | Clostridiaceae_1 | Firmicutes | Clostridia | Clostridiales | Clostridium_sensu_stricto_1 |  |
| 840001014-V1 | Clostridiaceae_1 | Firmicutes | Clostridia | Clostridiales | Clostridium_sensu_stricto_1 |  |
| 840001014-V1 | Burkholderiaceae | Proteobacteria | Gammaproteobacteria | Betaproteobacteriales | Sutterella | gut_metagenome |
| 840001014-V1 | Veillonellaceae | Firmicutes | Negativicutes | Selenomonadales | Veillonella |  |
| 840001014-V1 | Bifidobacteriaceae | Actinobacteria | Actinobacteria | Bifidobacteriales | Bifidobacterium |  |
| 840001014-V1 | Lachnospiraceae | Firmicutes | Clostridia | Clostridiales | [Eubacterium]_hallii_group |  |
| 840001014-V1 | Lachnospiraceae | Firmicutes | Clostridia | Clostridiales | Lachnospira |  |
| 840001014-V1 | Peptostreptococcaceae | Firmicutes | Clostridia | Clostridiales | Romboutsia |  |
| 840001014-V1 | Lachnospiraceae | Firmicutes | Clostridia | Clostridiales | Lachnoclostridium | human_gut_metagenome |
| 840001014-V1 | Lachnospiraceae | Firmicutes | Clostridia | Clostridiales | Lachnoclostridium | uncultured_Firmicutes_bacterium |
| 840001014-V1 | Lachnospiraceae | Firmicutes | Clostridia | Clostridiales |  |  |
| 840001014-V1 | Lachnospiraceae | Firmicutes | Clostridia | Clostridiales | [Ruminococcus]_torques_group |  |
| 840001014-V1 | Bifidobacteriaceae | Actinobacteria | Actinobacteria | Bifidobacteriales | Bifidobacterium |  |
| 840001014-V1 | Lachnospiraceae | Firmicutes | Clostridia | Clostridiales | Dorea |  |
| 840001014-V1 | Lachnospiraceae | Firmicutes | Clostridia | Clostridiales | Anaerostipes |  |
| 840001014-V1 | Lachnospiraceae | Firmicutes | Clostridia | Clostridiales | Blautia |  |
| 840001014-V1 | Lachnospiraceae | Firmicutes | Clostridia | Clostridiales | Lachnospiraceae_UCG-004 |  |
| 840001014-V1 | Lachnospiraceae | Firmicutes | Clostridia | Clostridiales | [Ruminococcus]_gnavus_group |  |
| 840001014-V1 | Bacteroidaceae | Bacteroidetes | Bacteroidia | Bacteroidales | Bacteroides |  |
| 840001014-V1 | Lachnospiraceae | Firmicutes | Clostridia | Clostridiales | Fusicatenibacter |  |
| 840001014-V1 | Lachnospiraceae | Firmicutes | Clostridia | Clostridiales |  |  |
| 840001014-V1 | Lachnospiraceae | Firmicutes | Clostridia | Clostridiales | Blautia |  |
| 840001014-V1 | Veillonellaceae | Firmicutes | Negativicutes | Selenomonadales | Dialister |  |
| 840001014-V1 | Lachnospiraceae | Firmicutes | Clostridia | Clostridiales | [Eubacterium]_hallii_group |  |
| 840001014-V1 | Lachnospiraceae | Firmicutes | Clostridia | Clostridiales | Lachnospiraceae_UCG-001 | Lachnospiraceae_bacterium_TF01-11 |
| 840001014-V1 | Lachnospiraceae | Firmicutes | Clostridia | Clostridiales | Dorea |  |
| 840001014-V1 | Lachnospiraceae | Firmicutes | Clostridia | Clostridiales | [Eubacterium]_hallii_group |  |
| 840001014-V1 | Tannerellaceae | Bacteroidetes | Bacteroidia | Bacteroidales | Parabacteroides | Parabacteroides_distasonis |
| 840001014-V1 | Lachnospiraceae | Firmicutes | Clostridia | Clostridiales | Blautia |  |
| 840001014-V1 | Lachnospiraceae | Firmicutes | Clostridia | Clostridiales | Lachnospiraceae_UCG-004 | uncultured_organism |
| 840001014-V1 | Streptococcaceae | Firmicutes | Bacilli | Lactobacillales | Streptococcus |  |
| 840001014-V1 | Lachnospiraceae | Firmicutes | Clostridia | Clostridiales | Anaerostipes |  |
| 840001014-V1 | Ruminococcaceae | Firmicutes | Clostridia | Clostridiales | Butyricicoccus |  |
| 840001014-V1 | Bacteroidaceae | Bacteroidetes | Bacteroidia | Bacteroidales | Bacteroides | Bacteroides_acidifaciens |
| 840001014-V1 | Lachnospiraceae | Firmicutes | Clostridia | Clostridiales | Blautia |  |
| 840001014-V1 | Lachnospiraceae | Firmicutes | Clostridia | Clostridiales | Dorea |  |
| 840001014-V1 | Peptostreptococcaceae | Firmicutes | Clostridia | Clostridiales | Intestinibacter |  |
| 840001014-V1 | Lachnospiraceae | Firmicutes | Clostridia | Clostridiales | Lachnospira |  |
| 840001014-V1 | Pasteurellaceae | Proteobacteria | Gammaproteobacteria | Pasteurellales | Haemophilus |  |
| 840001014-V1 | Lachnospiraceae | Firmicutes | Clostridia | Clostridiales | [Ruminococcus]_torques_group |  |
| 840001014-V1 | Lachnospiraceae | Firmicutes | Clostridia | Clostridiales | Agathobacter |  |
| 840001014-V1 | Lachnospiraceae | Firmicutes | Clostridia | Clostridiales | [Eubacterium]_ventriosum_group |  |
| 840001014-V1 | Bacteroidaceae | Bacteroidetes | Bacteroidia | Bacteroidales | Bacteroides |  |
| 840001014-V1 | Ruminococcaceae | Firmicutes | Clostridia | Clostridiales | Butyricicoccus |  |
| 840001014-V1 | Streptococcaceae | Firmicutes | Bacilli | Lactobacillales | Streptococcus |  |
| 840001014-V1 | Lachnospiraceae | Firmicutes | Clostridia | Clostridiales | Blautia |  |
| 840001014-V1 | Erysipelotrichaceae | Firmicutes | Erysipelotrichia | Erysipelotrichales | Erysipelatoclostridium |  |
| 840001014-V1 | Veillonellaceae | Firmicutes | Negativicutes | Selenomonadales | Veillonella |  |
| 840001014-V1 | Lachnospiraceae | Firmicutes | Clostridia | Clostridiales |  |  |
| 840001014-V1 | Lachnospiraceae | Firmicutes | Clostridia | Clostridiales | Tyzzerella_4 | uncultured_organism |
| 840001014-V1 | Lactobacillaceae | Firmicutes | Bacilli | Lactobacillales | Lactobacillus |  |
| 840001014-V1 | Bacteroidaceae | Bacteroidetes | Bacteroidia | Bacteroidales | Bacteroides | Bacteroides_thetaiotaomicron |
| 840001014-V1 | Actinomycetaceae | Actinobacteria | Actinobacteria | Actinomycetales | Actinomyces |  |
| 840001014-V1 | Lachnospiraceae | Firmicutes | Clostridia | Clostridiales | Lachnoclostridium |  |
| 840001014-V1 | Erysipelotrichaceae | Firmicutes | Erysipelotrichia | Erysipelotrichales | [Clostridium]_innocuum_group |  |
| 840001014-V1 | Lactobacillaceae | Firmicutes | Bacilli | Lactobacillales | Lactobacillus |  |
| 840001014-V1 | Lactobacillaceae | Firmicutes | Bacilli | Lactobacillales | Lactobacillus | Lactobacillus_fermentum |
| 840001014-V1 | Ruminococcaceae | Firmicutes | Clostridia | Clostridiales | Ruminiclostridium_5 | uncultured_organism |
| 840001014-V1 | Lactobacillaceae | Firmicutes | Bacilli | Lactobacillales | Lactobacillus |  |
| 840001014-V1 | Micrococcaceae | Actinobacteria | Actinobacteria | Micrococcales | Rothia | uncultured_bacterium |
| 840001014-V1 | Veillonellaceae | Firmicutes | Negativicutes | Selenomonadales | Veillonella | uncultured_methanogenic_archaeon |
| 840001014-V1 | Atopobiaceae | Actinobacteria | Coriobacteriia | Coriobacteriales | Atopobium | uncultured_bacterium |
| 840001014-V1 | Tannerellaceae | Bacteroidetes | Bacteroidia | Bacteroidales | Parabacteroides | Parabacteroides_goldsteinii_CL02T12C30 |
| 840001014-V1 | Erysipelotrichaceae | Firmicutes | Erysipelotrichia | Erysipelotrichales | Erysipelatoclostridium |  |
| 840001014-V1 | Erysipelotrichaceae | Firmicutes | Erysipelotrichia | Erysipelotrichales | Erysipelatoclostridium | Massiliomicrobiota_timonensis |
| 840001014-V1 | Lachnospiraceae | Firmicutes | Clostridia | Clostridiales | Lachnoclostridium | [Clostridium]_scindens |
| 840001014-V1 | Lachnospiraceae | Firmicutes | Clostridia | Clostridiales | uncultured |  |
| 840001014-V1 | Eggerthellaceae | Actinobacteria | Coriobacteriia | Coriobacteriales | Slackia | Slackia_sp._S4-C6 |
